# Signal-induced recruitment and dispatchment in reconfigurable phase-separated protocell networks

**DOI:** 10.1038/s41557-026-02225-3

**Published:** 2026-08-18

**Authors:** Zhuping Yin, Rui Sun, Mei Li, Stephen Mann

**Affiliations:** 1 Centre for Protolife Research and Centre for Organized Matter Chemistry, School of Chemistry, University of Bristol, Bristol, UK; 2 Department of Bio-Organic Chemistry, Institute of Complex Molecular Systems, Eindhoven University of Technology, Eindhoven, the Netherlands; 3 Max Planck-Bristol Centre for Minimal Biology, School of Chemistry, University of Bristol, Bristol, UK

**Keywords:** Origin of life, Surface assembly

## Abstract

The construction of protocell networks with self-regulated spatial dynamics and functions is an important challenge in the emerging field of colloidal systems chemistry. Existing strategies predominantly produce protocell networks with fixed or randomly distributed spatial organization, relying on direct surface interactions or externally imposed conditions, while largely overlooking dynamic interactions with the surrounding environment, thereby limiting the emergence of reconfigurable network behaviours. Here we demonstrate chemical strategies for implementing the spontaneous segregation and selective translocation of binary/ternary populations of enzyme-containing proteinosomes in dextran droplet/polyethylene glycol aqueous phase-separated media. The segregated proteinosomes exhibit tunable membrane wettability, engage in cross-community chemical signalling and undergo signal-induced reversals in phase compatibility to produce reconfigurable networks capable of protocell-mediated recruitment and dispatchment. We exploit the protocell phase dynamics to spatiotemporally modulate DNase I activity in a subpopulation of translocating proteinosomes. Our methodology provides a platform for developing protocell communities with self-regulated spatiotemporal order and offers opportunities in cytomimetic modelling and colloidal systems chemistry.

**Figure:**
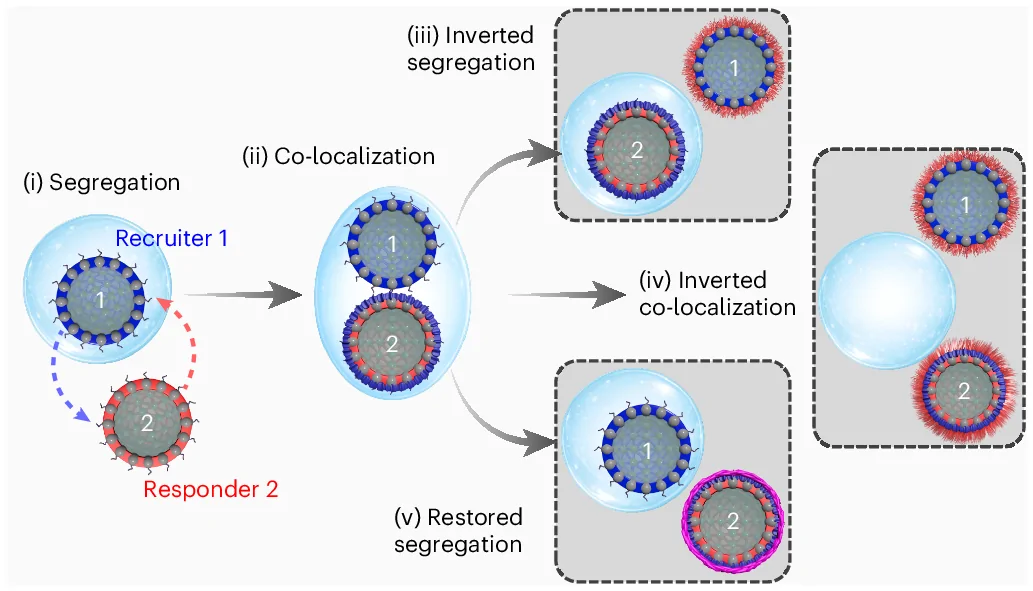

## Abstract (web summary)

Constructing protocell networks with spatial dynamics remains a central challenge in colloidal systems chemistry. Now, binary populations of enzyme-containing protocells with different membrane wettability have been shown to spontaneously segregate in phase-separated media. Enzyme-mediated signals induce reversible phase compatibility, enabling selective protocell recruitment and dispatchment, and the construction of cytomimetic networks with self-regulated spatiotemporal order.

## Main

The spatial segregation of living cells into functionally integrated networks is a hallmark of biological organization, underpinning processes such as tissue self-organization^1,2^ and microbial patterning^3,4^. These spatially structured systems enable coordinated cell–cell communication and dynamic cell–environment interactions, which are essential for the emergence of collective behaviours, adaptive functionality and higher-order spatial organization. Abstracting these biological processes into segregated populations of protocells with signal-responsive cytomimetic properties such as gene expression^5,6^, chemo-mechanical responsiveness^7^, morphological differentiation^8^ and structural hybridization^9–11^ provides an important step in the development of interactive networks of life-like entities and systems capable of chemical communication^12–17^, collective movement^18–20^ and synthetic multicellularity^21–23^.

Mixed and interactive protocell populations can be spatially ordered using acoustic pressure fields^8,16^ and microfluidic traps^12^ or aggregated into clusters (prototissues) by complementary surface interactions^21^, magnetic fields^24,25^, microfluidics^26^ and three-dimensional printing^27^. However, the ensuing networks often exhibit only low levels of differentiation due to the fixed spatial positioning and homogeneous distribution of the constituent protocells. Similarly, multiple populations of protocells distributed within interactive hydrogel matrices are randomly mixed and spatially immobilized^28,29^, restricting their capacity for spatiotemporal reorganization. Thus, it remains challenging to construct dynamical protocell networks in which the subpopulations are spontaneously segregated into spatial arrangements that can be internally reconfigured.

To address this issue, here we present a chemical strategy for establishing programmable self-organization and signal-induced translocation in segregated protocell networks housed in aqueous phase-separated media. So far, protocell sorting mechanisms based on selective membrane adhesion in dispersed populations^30–32^, microfluidic-mediated partitioning in Janus hydrogel microparticles^33^, selective wetting in coacervate droplets^34^ or differential rates of coalescence in assembling chains of coacervate or coacervate/dextran droplet superstructures^35–37^ have been demonstrated. In this Article, we use an aqueous two-phase system (ATPS) consisting of dextran-rich droplets in a continuous polyethylene glycol (PEG) phase as a platform for the segregation and subsequent reconfiguration of phase-separated protocell networks. By activating enzyme-mediated signalling processes, our design seeks to achieve programmable reconfiguration of protocell networks by selective phase translocation of subpopulations, allowing spatiotemporal functional modulation within protocells. Our approach provides a feasible route to the construction of cytomimetic networks with increased complexification and environmental responsiveness and offers opportunities in the study of artificial multicellularity and colloidal systems chemistry.

## Results and discussion

### Spatial segregation of protocell populations in aqueous phase-separated media

**Fig. 1:**
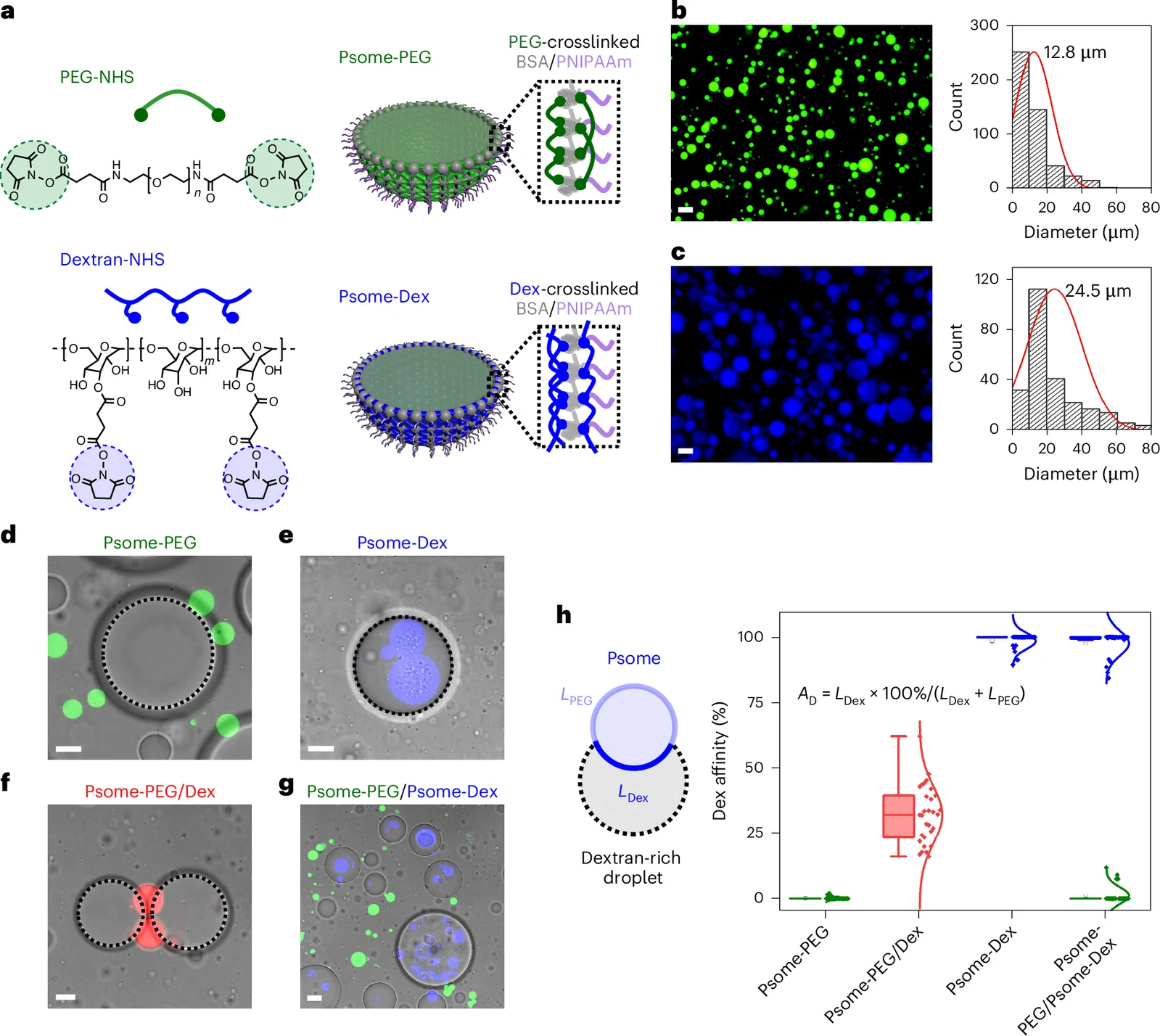
Spatial segregation of protocell populations in aqueous phase-separated media. **a**, Scheme illustrating the construction of proteinosomes with tailored wetting properties. NHS-activated PEG (green line) or NHS-activated dextran (blue line) are used to cross-link BSA (grey dots)/PNIPAAm (wavy light-purple lines) nanoconjugates in the proteinosome membrane to produce Psome-PEG or Psome-Dex protocells with PEG or dextran compatibility, respectively. **b**,**c**, Fluorescence images (left column) of FITC-labelled Psome-PEG (**b**, green fluorescence) and Dylight 405-Psome-Dex (**c**, blue fluorescence) protocells after transfer into water, and corresponding proteinosome size distributions and mean values (right column). Fluorescent labels were attached to the BSA/PNIPAAm nanoconjugates before protocell assembly. Fluorescence is observed throughout the protocells due to excess nanoconjugates within the proteinosome interior. **d**–**f**, CLSM images showing phase localization of single proteinosome populations in a dextran-in-PEG ATPS. FITC-labelled Psome-PEG constructs (green fluorescence) are dispersed in the continuous PEG-enriched phase (**d**), Dylight 405-labelled Psome-Dex (blue fluorescence) protocells are captured within the dextran-enriched droplets (**e**) and RITC-labelled Psome-PEG/Dex (red fluorescence) micro-compartments are located at the dextran droplet/PEG interface (**f**). Black dash circles delineate the edge of the dextran droplets in the ATPS. **g**, CLSM image showing spatial segregation of a binary proteinosome population in a dextran-in-PEG ATPS. Under vortex, a mixture of FITC-Psome-PEG (green fluorescence) and Dylight 405-Psome-Dex (blue) protocells are spatially sorted within 10 s into the dextran droplets and continuous PEG phase, respectively. Protocell number densities; 0.6 × 10^7^ ml^−1^ (Psome-Dex); 1.0 × 10^7^ ml^−1^ (Psome-PEG). **h**, Statistical analysis of the spatial localization of single populations of Psome-PEG, Psome-Dex and Psome-PEG/Dex protocells and a Psome-PEG/Psome-Dex binary population when distributed in an ATPS. The analysis is based on the number of proteinosomes present in the dextran-enriched droplets after 10 min. The percentage dextran affinity (*A*_D_) is given by *A*_D_ = *L*_Dex_/(*L*_Dex_ + *L*_PEG_) × 100**%**, where *L*_Dex_ and *L*_PEG_ are the edge lengths of proteinosomes in contact with the dextran-rich droplets and PEG-rich medium of the ATPS, respectively. Box plots show the median (centre line), the interquartile range (box, 25th–75th percentiles) and the minimum and maximum values (whiskers). Error bars: *n* = 30, s.d. 0.5% (Psome-PEG), 11.0% (Psome-PEG/Dex), 3.0% (Psome-Dex) in the single population of Psome in ATPS. *n* = 30, s.d. 4.7% (Psome-Dex) and 3.2% (Psome-PEG) in the binary population of Psome in ATPS. All scale bars, 20 µm. Source data

An ATPS consisting of membraneless dextran-enriched droplets dispersed in a PEG-enriched continuous phase was prepared by vortexing mixtures of the polysaccharide (dextran; ~35 mg ml^−1^, molecular weight (MW) 35–45 kDa) and polymer (PEG, ~104 mg ml^−1^, MW 35 kDa) at 1,000 rpm for 30 s at room temperature. Typically, the as-prepared dextran-enriched droplets were heterogeneous in size (mean diameter 25.2 ± 19.4 µm; range 10–75 µm) and underwent coalescence within 30 min to produce a bulk phase if not continually stirred. Proteinosomes (Psome) were prepared as previous described^38^ by the spontaneous self-assembly and cross-linking of amphiphilic bovine serum albumin (BSA)/poly(*N*-isopropylacrylamide) (PNIPAAm) nanoconjugates at enzyme-containing water droplet–oil interfaces, followed by transfer of the semi-permeable protocells and entrapped biocatalysts into water. To construct protocells with appropriate wetting compatibility for either the PEG- or dextran-enriched domains of an ATPS, we used respectively a previously employed *N*-hydroxysuccinimide (NHS)-activated PEG polymer^38^ (*O*,*O*′-bis[2-(*N*-succinimidyl-succinylamino)ethyl]PEG; PEG-NHS; MW 2 kDa; Supplementary Fig. 1) or an NHS-activated carboxylated dextran polymer (Dex-NHS; MW 40 kDa; NHS monomer ratio 10%; Supplementary Fig. 1) to cross-link the BSA/PNIPAAm membrane by amination between the primary amino-containing BSA domains of the membrane and NHS ester-containing cross-linkers (Fig. 1a). Both procedures gave rise to aqueous dispersions of intact spherical membrane-enclosed proteinosomes with diameters of 12.8 ± 10.2 µm (Psome-PEG) or 24.5 ± 15.5 µm (Psome-Dex) (Fig. 1b,c and Supplementary Fig. 2). Zeta potential measurements indicated that both types of proteinosome had negatively charged membranes (approximately −20 mV at pH 7 (Supplementary Fig. 3). In both variants, the cross-linked proteinosome membrane was semi-permeable with an effective MW cutoff (MWCO) of ~70 kDa (Supplementary Fig. 4). Consequently, a range of different enzymes (Supplementary Figs. 5 and 6) could be encapsulated within the protocells.

Addition of single populations of Psome-PEG or Psome-Dex to a dextran-in-PEG ATPS confirmed the differences in protocell wettability with confocal laser scanning microscopy (CLSM) images showing that the Psome-PEG constructs were dispersed within the continuous PEG phase while the Psome-Dex protocells were predominantly confined to the dextran-enriched droplets (Fig. 1d,e). Protocells cross-linked by a mixture of PEG-NHS and dextran-NHS were predominantly located at the droplet interfaces in the ATPS (Fig. 1f and Supplementary Fig. 7). Consequently, addition of a binary population of Psome-PEG and Psome-Dex protocells to the ATPS at respective number densities of 1.0 × 10^7^ ml^−1^ and 0.6 × 10^7^ ml^−1^ gave rise to spontaneous sorting of the proteinosome mixture over 10 s or 30 min under vortex or quiescent conditions, respectively, to produce a segregated protocell community (Fig. 1g and Supplementary Fig. 8). Statistical analyses of the localization and segregation of single and binary proteinosome populations, respectively, confirmed the CLSM observations (Fig. 1h). In the absence of stirring, time-dependent confocal microscopy images revealed that the Psome-PEG constructs in the continuous PEG-rich phase exhibited confined Brownian motion, while the dextran-cross-linked proteinosomes moved freely until in contact with a dextran droplet, after which they transferred across the interface within 15 s (Supplementary Fig. 9). Unstirred systems gradually coalesced over 24 h to produce a bulk ATPS with segregated protocell populations (Supplementary Fig. 10).

### Protocell phase transfer by endogenously engineered membrane augmentation

**Fig. 2:**
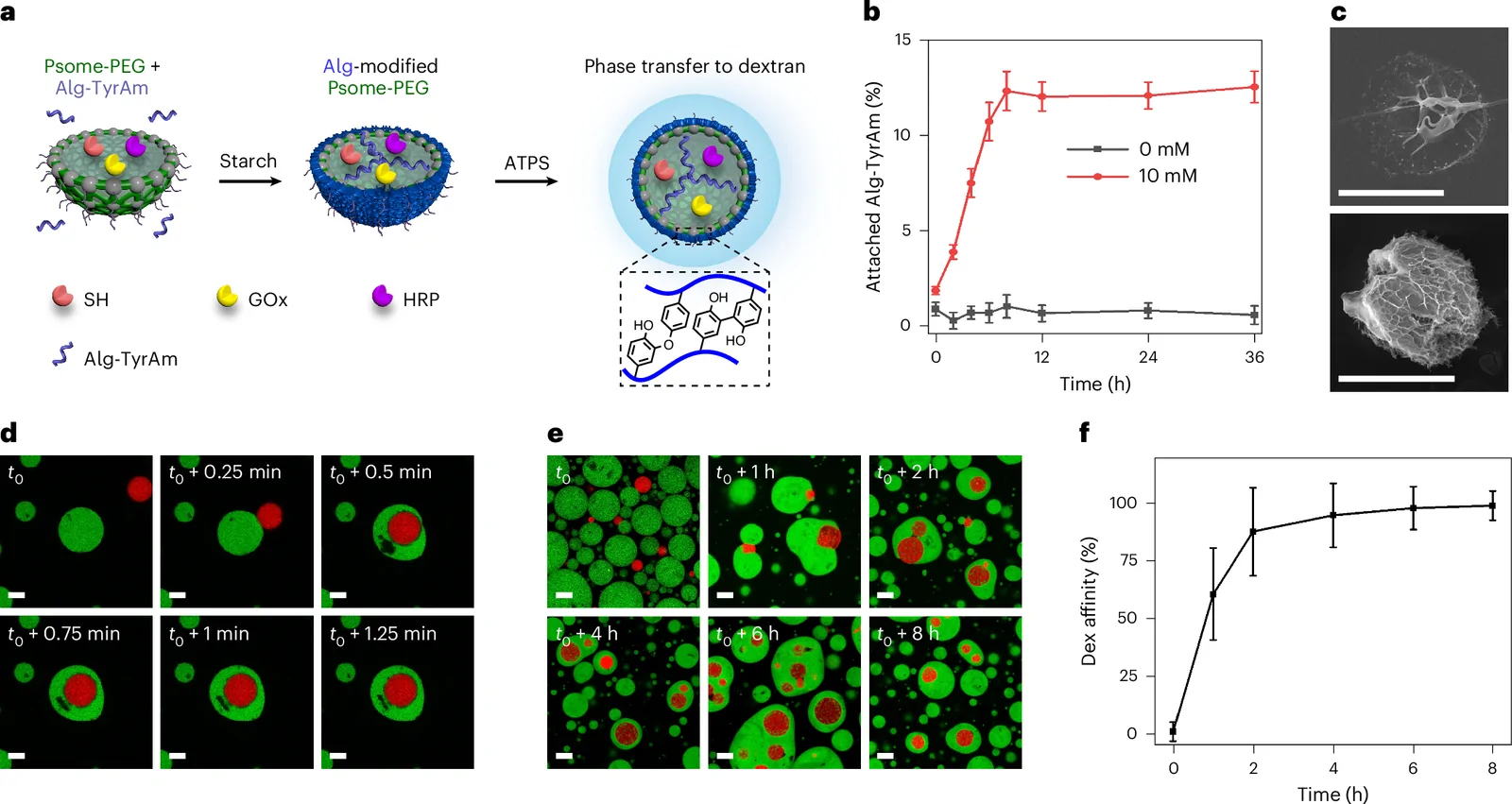
Self-activated PEG-to-dextran protocell phase transfer. **a**, Scheme showing post-assembly attachment of the polysaccharide Alg-TyrAm onto the membrane of SH/GOx/HRP-containing Psome-PEG protocells by addition of starch. The modified protocells translocate from the PEG phase into the dextran droplets (light blue) of an ATPS. **b**, Time-dependent plot of the percentage of Alg-TyrAm grafted onto the Psome-PEG membrane in the absence (0 mM) or after addition of glucose (10 mM). **c**, SEM images of single Psome-PEG protocells before (top) and after (bottom) membrane modification with a Alg-TyrAm. **d***,* Time sequence of CLSM images showing translocation of a single Alg-TyrAm-modified Psome-PEG protocell (red fluorescence, RITC-BSA/PNIPAAm membrane nanoconjugate) from the PEG continuous phase into a dextran droplet (green fluorescence, FITC-labelled dextran). **e**, Time series of CLSM images showing translocation of Psome-PEG protocells (red fluorescence) from the continuous PEG phase into the dextran droplets (green fluorescence) of an ATPS after addition of glucose at t_0_ in the presence of Alg-TyrAm. Fluorescent labels as in **d**. **f**, Time-dependent plot of number of Alg-TyrAm-modified SH/GOx/HRP/Psome-PEG protocells present in the dextran-enriched droplets (dextran affinity) of an ATPS. Protocell number densities; SH/GOx/HRP-Psome-PEG, 1.8 × 10^7^ ml^−1^. Error bars: *n* = 3 (repeated groups of samples), mean values ± s.d. in **b**; *n* (protocells) = 30 from ≥3 CLSM images at each timepoint, mean values ± s.d. in **f**. Scale bars, 20 µm (**c**–**e**). Source data

Having established that the integration of PEG- or dextran-based cross-linkers into the proteinosome membrane during construction facilitates spontaneous spatial segregation of the binary protocell populations in aqueous phase-separated media, we used a protocell-encapsulated three-enzyme cascade consisting of starch hydrolase (SH, α-amylase/amyloglucosidase, MW 50/97 kDa), glucose oxidase (GOx, MW 160 kDa) and horseradish peroxidase (HRP, MW 40 kDa)^10^ to implement in situ changes in protocell membrane wettability (Supplementary Fig. 11). SH was used to convert a starch input into glucose, which in turn initiated GOx activity within the protocells to produce fluxes of H_2_O_2_ and gluconic acid that triggered different in situ changes in protocell phase compatibility in an ATPS. Specifically, Psome-PEG constructs preloaded with SH, GOx and HRP were rendered dextran-compatible by addition of dissolved starch to initiate the endogenous generation of H_2_O_2_ in the presence of tyramine-derivatized alginate (Alg-TyrAm, <1 mg ml^−1^; TyrAm content 7% (n/n), MW 140–160 kDa; Supplementary Fig. 12) located within the external aqueous phase (Fig. 2a). Consequently, HRP/H_2_O_2_-mediated oxidative cross-linking of Alg-TyrAm produced a polysaccharide coating on the outer surface of the Psome-PEG membrane as well as within the protocells over a period of 8 h under mild vortex (600 rpm) (Fig. 2b,c and Supplementary Fig. 13). Notably, polymerization in the bulk phase of the protocell dispersion was minimal, indicating that the peroxidation mechanism was strongly influenced by spatial confinement of HRP. One possibility is that diffusional constraints imposed by rapid quenching of short-lived tyrosine radicals ensure that polymerization occurs near to the encapsulated HRP molecules rather than at a distance in bulk solution. Increasing the Alg-TyrAm concentration to 2 mg ml^−1^ resulted in clustering of the surface-modified proteinosomes (Supplementary Fig. 14). When added to an ATPS, single and clustered Alg-TyrAm-modified Psome-PEG protocells transferred freely into the dextran droplets typically within 15–30 s once contact with the phase boundary occurred (Fig. 2d and Supplementary Fig. 15).

Based on the above observations, we established endogenous enzyme-mediated mechanisms for spontaneous protocell phase transfer in a dextran-PEG ATPS. Translocation of SH/GOx/HRP-containing Psome-PEG protocells from the PEG continuous phase to the dextran droplets was achieved by activating Alg-TyrAm polymerization within the ATPS. Although starch, glucose and Alg-TyrAm were preferentially or equally distributed in the dextran droplets (partition coefficients (*K* (Dex/PEG); 11.5 (starch, Supplementary Fig. 16); 1.1 (glucose, Supplementary Fig. 16); 5.0 (alginate, Supplementary Fig. 17)), the level of oxidative cross-linking in the PEG phase was sufficient to change the wettability of the proteinosome membrane such that Alg-TyrAm-modified Psome-PEG protocells were progressively transferred into the dextran droplets (Fig. 2e,f and Supplementary Fig. 18).

To allow a direct assessment of the influence of glucose levels on H_2_O_2_ and gluconic acid production, we removed the co-encapsulated SH to bypass the initial SH-mediated step. The results indicated that the time required to complete the translocation of the entire Psome-PEG protocell population decreased progressively from 6 to 2 h as the concentration of added glucose increased under constant Alg-TyrAm concentrations (1 mg ml^−1^) (Supplementary Fig. 19). Propagation of H_2_O_2_ was effective at glucose concentrations as low as 3 mM, although the percentage of Alg-TyrAm-modified Psome-PEG protocells transferred into the dextran droplets (dextran affinity) after 2 h was ~32% lower than at 50 mM glucose. The dextran affinity was also dependent on the Alg-Tyr concentration with levels below or above 0.2 mg ml^−1^ associated with incomplete or complete transfer after 5 h, respectively, at a constant glucose level of 10 mM (Supplementary Fig. 20). Measurements of the coproduction of gluconic acid associated with GOx activity within the GOx/HRP-loaded Psome-PEG protocells indicated that the rate of decrease in pH was strongly dependent on glucose concentration and that mild acidification (pH 5) of the ATPS was achieved at glucose levels of 25 mM after 12 h (Supplementary Fig. 21).

**Fig. 3:**
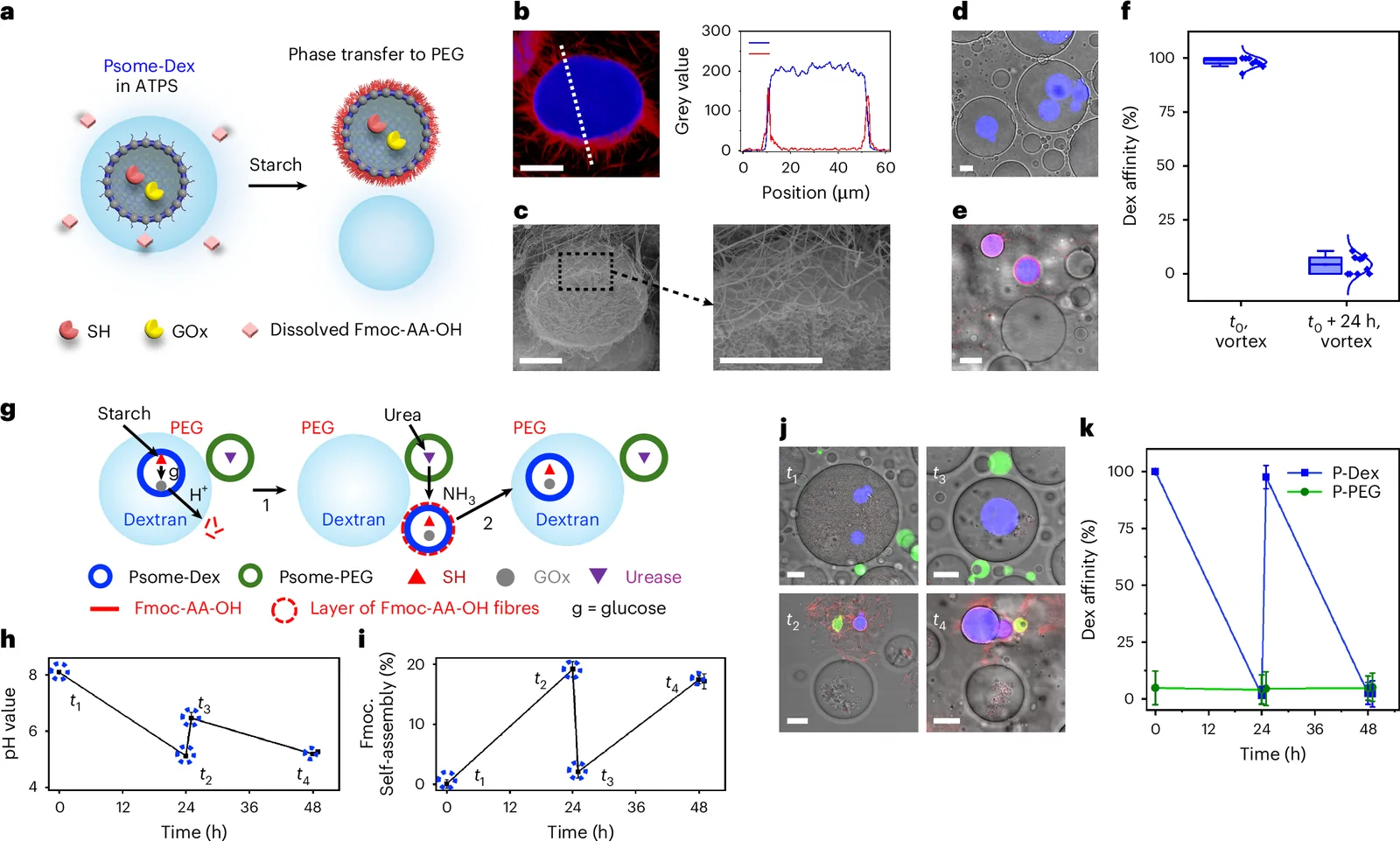
Self-mediated dextran-to-PEG protocell phase transfer. **a**, Scheme showing post-assembly acid-induced attachment of Fmoc-AA-OH peptide fibres onto the membrane of SH/GOx-containing Psome-Dex protocells by addition of starch, followed by transfer from the dextran droplets (light-blue filled circles) to the PEG phase of an ATPS. **b**, left**;** three-dimensional stacked CLSM image of a Psome-Dex protocell (blue fluorescence, Dylight 405) coated in peptide fibres (red fluorescence, Nile red), and corresponding fluorescence profile (right) recorded along the displayed white dotted line. **c**, SEM images of a peptide fibril-coated Psome-Dex protocell recorded at low (left) and high (right) magnification. **d–f**, CLSM images of an ATPS containing SH/GOx/Psome-Dex protocells (blue fluorescence) before (**d**, pH 8.0) and after (**e**, pH 5.0) addition of glucose showing translocation from the dextran droplets to the PEG continuous phase due to peptide fibre-induced changes in membrane wettability. The corresponding plots of the percentage of dextran-affinity of the Psome-Dex protocells with time are shown (**f**). Box plots show the median (centre line), the interquartile range (box, 25th–75th percentiles) and the minimum and maximum values (whiskers). **g**, Schematic showing cyclic phase transfer of SH/GOx-loaded Psome-Dex protocells (blue open circle). Step 1, starch/GOx-Psome-Dex-mediated peptide fibril assembly, protocell membrane modification and translocation into PEG; step 2, urea/urease-mediated peptide fibril disassembly, membrane remodelling and transfer back to dextran. Step 2 is mediated by a population of urease-loaded Psome-PEG protocells (green open circle) present in the PEG continuous phase. **h**,**i**, Time-dependent plots of pH (**h**) and Fmoc-AA-OH fibril assembly (**i**) in an ATPS containing an initially segregated binary population of SH/GOx-containing Psome-Dex and urease-loaded Psome-PEG protocells. Starch (50 mM) was added at *t*_1_. Urea (5 mM) was added at *t*_2_ and *t*_4_. **j**,**k**, Corresponding CLSM images (**j**) recorded at different times in the protocell phase transfer process and plots of the percentages of dextran droplet-located Psome-Dex and Psome-PEG protocells (**k**). Protocell number densities; SH/GOx-Psome-Dex, 0.6 × 107 ml^−1^; urease/Psome-PEG, 1.8 × 10^7^ ml^−1^. Peptide fibres in **j** are stained using Nile red. Error bars: *n* = 10, s.d. 2.3% (*t*_0_) and 4.2% (*t*_0_ + 24 h) in **f**; *n* = 3, mean values ± s.d. in **h** and **i**; *n* = 10, mean values ± s.d. in **k**. Scale bars, 20 µm (**b**, **c** (left), **d**, **e** and **j**) and 10 µm (**c**, right). Source data

Using a similar strategy, SH/GOx-containing Psome-Dex protocells, initially confined within the dextran droplets of an ATPS at pH 8, were rendered PEG-compatible by enzyme-mediated peptide fibre formation on the surface of the proteinosome membrane (Fig. 3a and Supplementary Fig. 11). Addition of starch (or glucose) to the ATPS in the presence of a pH-responsive dipeptide (Fmoc-AA-OH monomer) distributed preferentially in the PEG continuous phase (*K* (Dex/PEG) = 0.4 (pH 9)); Supplementary Fig. 22) activated the production of gluconic acid, subsequently acidified the media and triggered peptide supramolecular assembly at pH values below 5.3 (Supplementary Fig. 23). This threshold pH required starch concentrations above 25 mM, and incubation times of 18 h and Fmoc-AA-OH concentrations above 2 mg ml^−1^ to reverse the wettability of the Psome-Dex protocells in the ATPS (Supplementary Fig. 24). CLSM images of individual Psome-Dex protocells showed the presence of a dense layer of dipeptide fibrils on the outer surface of the proteinosome membrane after GOx activity (Fig. 3b). Scanning electron microscopy (SEM) images confirmed the presence of an interconnected radiating network of self-assembled fibrils with thicknesses of 50–250 nm (Fig. 3c and Supplementary Fig. 25). By contrast, negligible amounts of the dipeptide fibrils were observed inside the modified Psome-Dex protocells. Interestingly, although Fmoc-AA-OH fibres were also observed in the continuous PEG phase, changes in the membrane wettability associated with the peptide coating surface were sufficient to enable translocation of the membrane-modified proteinosomes from the dextran droplets into the PEG-enriched continuous phase under vortex (Fig. 3d,e and Supplementary Fig. 26). This was statistically confirmed by measuring the decrease in dextran compatibility of the protocell population before and 24 h after surface modification (Fig. 3f).

Based on these observations, we used a segregated binary population of enzyme-containing proteinosomes to achieve cyclic transfer of a single protocell population between the dextran and PEG phases of the ATPS (Fig. 3g). SH/GOx-loaded Psome-Dex protocells, initially located within the dextran droplets in the presence of Fmoc-AA-OH, were spontaneously translocated into the continuous PEG phase by starch-induced pH-mediated fibre assembly (pH <5.3) and then transferred back to the dextran droplets by subsequent disassembly of the peptide coating under close-to-neutral pH conditions (Fig. 3h). The latter was achieved by adding urea to initiate urease activity and an increase in pH within a co-located population of urease-containing Psome-PEG protocells (Fig. 3i). Typically, it took approximately 24 h to cycle the SH/GOx-loaded proteinosome population between the dextran and PEG phases of the ATPS (Fig. 3j,k). The overall reversibility of the system was constrained to approximately 1.5 cycles by the gradual loss of urease activity when encapsulated within the proteinosomes under acidic conditions. However, increasing the bulk urease concentration in the ATPS gave three complete transfer cycles by repeated additions of urea and increasing the starch concentration 75 mM (Supplementary Fig. 27). At least six transfer cycles could be achieved when NaOH was used in place of urease as the pH-restoring agent (Supplementary Fig. 27).

### Signal-induced recruitment in phase-separated protocell networks

**Fig. 4:**
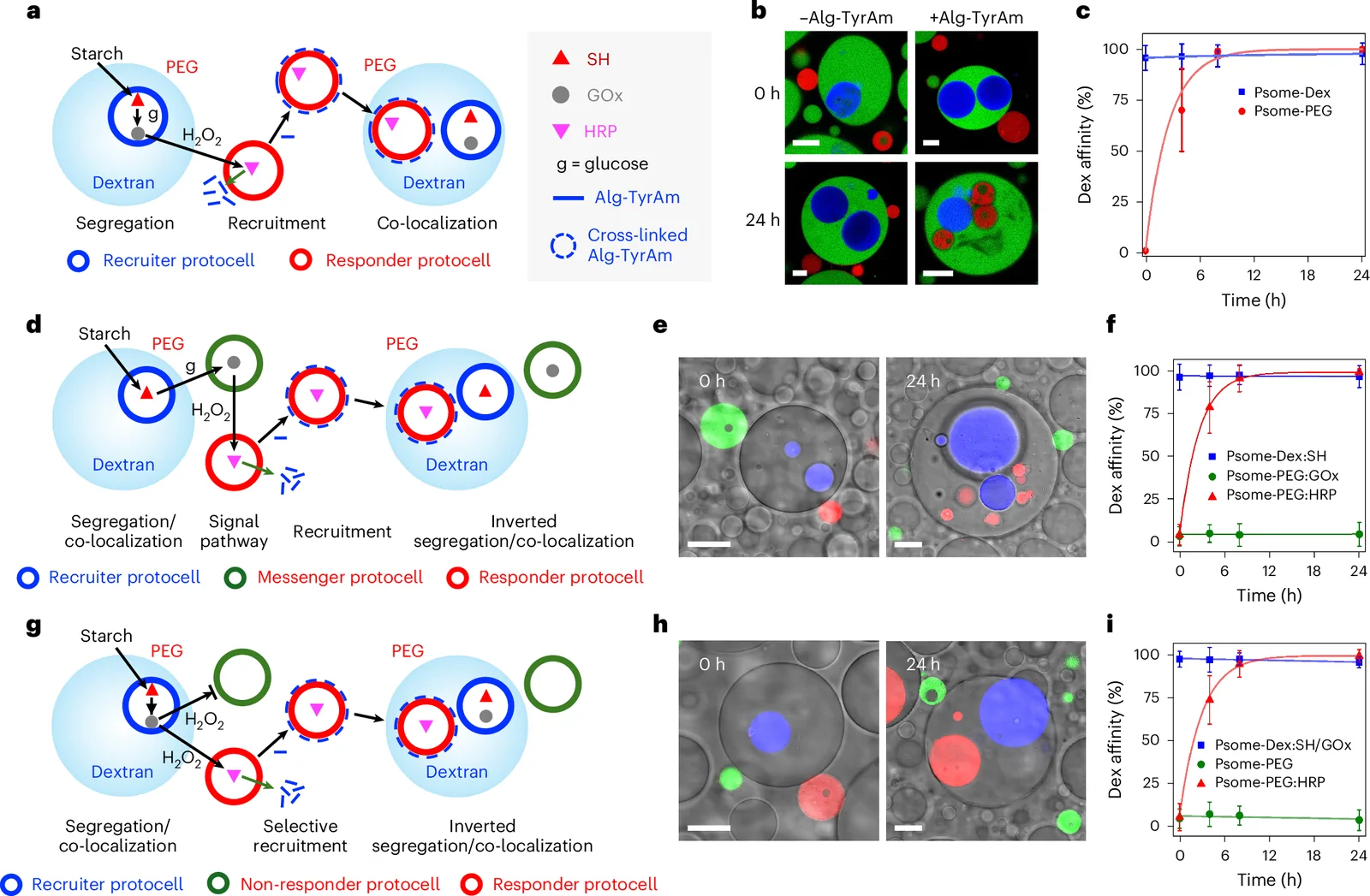
Signal-induced recruitment in phase-separated protocell networks. **a**–**i**, Three interrelated enzyme-mediated strategies are shown: direct recruitment in a segregated binary proteinosome population to produce a co-located network (**a**–**c**), or indirect recruitment (**d**–**f**) and selective recruitment (**g**–**i**) in ternary populations showing spatial inversion of the segregated ternary network. Schematics (**a**, **d** and **g**): Psome-Dex recruiter (blue open circles) and Psome-PEG responder (red open circles) protocells are initially located respectively in the dextran droplets (light-blue filled circles) and PEG continuous phase of an ATPS. A H_2_O_2_ diffusive signal is used to communicate with the responders. Coating of the responder membrane with dextran-compatible Alg-TyrAm fibres (blue lines) gives rise to their recruitment into the dextran droplets. SH and GOx are co-encapsulated within the recruiter protocells in **a**; GOx-containing Psome-PEG protocells are used as messengers in the PEG phase (green open circle) in **d**; and a subpopulation of non-enzymatic proteinosomes in the PEG phase is used as non-responder protocells (green open circle) in **g**. CLSM data images (**b**, **e** and **h**) of single dextran droplets recorded before (0 h) and 24 h after addition of starch (10 mM) in the presence of Alg-TyrAm (and absence of Alg-TyrAm in **b**). Fluorescence labels: dextran droplets (FITC, green, **b**); Dylight 405-Psome-Dex recruiters (blue); RITC-Psome-PEG responders (red); Psome-PEG messengers (FITC, green, **e**); Psome-PEG non-responders (FITC, green, **h**). Plots (**c**, **f** and **i**) of the percentage of dextran droplet-located recruiter and recruited protocells (dextran affinity) at different times after addition of starch (10 mM) showing co-localization of the binary population (**c**) and spatial inversion of the segregated ternary community (**f** and **i**). Protocell number densities: Psome-Dex (recruiter) 0.6 × 10^7^ ml^−1^, Psome-PEG (responder) 1.2 × 10^7^ ml^−1^ (**a**–**c**); 0.45 × 10^7^ ml^−1^ (recruiters), 0.6 × 10^7^ ml^−1^ (responders), 0.6 × 10^7^ ml^−1^ (messengers), 0.6 × 10^7^ ml^−1^ (non-responders) (**d**–**i**). Error bars: *n* (protocells) = 30 from ≥3 CLSM images at each timepoint, mean values ± s.d. in (**c**, **f** and **i**). All scale bars, 20 µm. Source data

Given that changes in the wettability of the proteinosome membrane could be established during and after protocell construction by the judicious choice of cross-linker and membrane coating agent, respectively, we designed experimental protocols for implementing the protocell-mediated signal-induced recruitment of targeted proteinosomes in a liquid–liquid phase-separated binary protocell population. To initiate interprotocell recruitment into the dextran droplets of an ATPS, SH/GOx-containing Psome-Dex constructs (signal-generating recruiter protocells) were segregated into the dextran phase and activated by addition of starch under constant stirring. Consequently, the resulting production of H_2_O_2_ served as a diffusive signal for directly triggering the enzyme-mediated assembly of a dextran-compatible Alg-TyrAm layer on the surface of HRP-containing Psome-PEG constructs (signal-receiving responder protocells) initially located in the PEG continuous phase (Fig. 4a and Supplementary Fig. 28). The corresponding change in membrane wettability resulted in progressive transfer of the modified responder protocells into the dextran droplets to produce a co-localized binary population within ~9 h for a starch input of 10 mM (Fig. 4b,c and Supplementary Fig. 29). Notably, only a small decrease in pH from 8 to 7.5 was observed due to the relatively low concentration (10 mM) of the starch input (Supplementary Fig. 30). Control experiments undertaken in the absence of Alg-TyrAm indicated that the GOx-mediated H_2_O_2_ flux increased with increasing starch concentration and implied that the signal threshold was below 0.35 mM (Supplementary Fig. 31).

The above strategy was further elaborated by using a ternary population of protocells to generate a multistep signalling pathway to targeted recruitment in phase-separated media containing a spatially distributed protocell network (Fig. 4d). The protocell community consisted of a single SH-containing recruiter population housed in the dextran droplets and a binary population of GOx-containing messenger proteinosomes and HRP-loaded responder protocells co-located in the continuous PEG phase containing Alg-TyrAm. Addition of starch resulted in phase transfer of glucose from the recruiter to messenger protocells and a two-step signalling cascade that gave rise to the selective recruitment of the HRP-loaded Psome-PEG constructs (Fig. 4e and Supplementary Fig. 32). As the messenger protocells remained in the PEG phase, the reconfiguration produced a segregated/co-located proteinosome network with inverted organization (Fig. 4f). Similar observations were made for a network consisting of SH/GOx-containing Psome-Dex recruiters and a mixture of Psome-PEG protocells prepared with or without encapsulated HRP and activated with Alg-TyrAm and starch (Fig. 4g,i and Supplementary Fig. 32). Consequently, the HRP-loaded protocells were selectively recruited into the dextran droplets owing to changes in membrane wettability, while the enzymatically non-active Psome-PEG population were unresponsive to the H_2_O_2_ recruitment signal and remained in the PEG phase.

### Selective dispatchment from phase-localized binary protocell populations

**Fig. 5:**
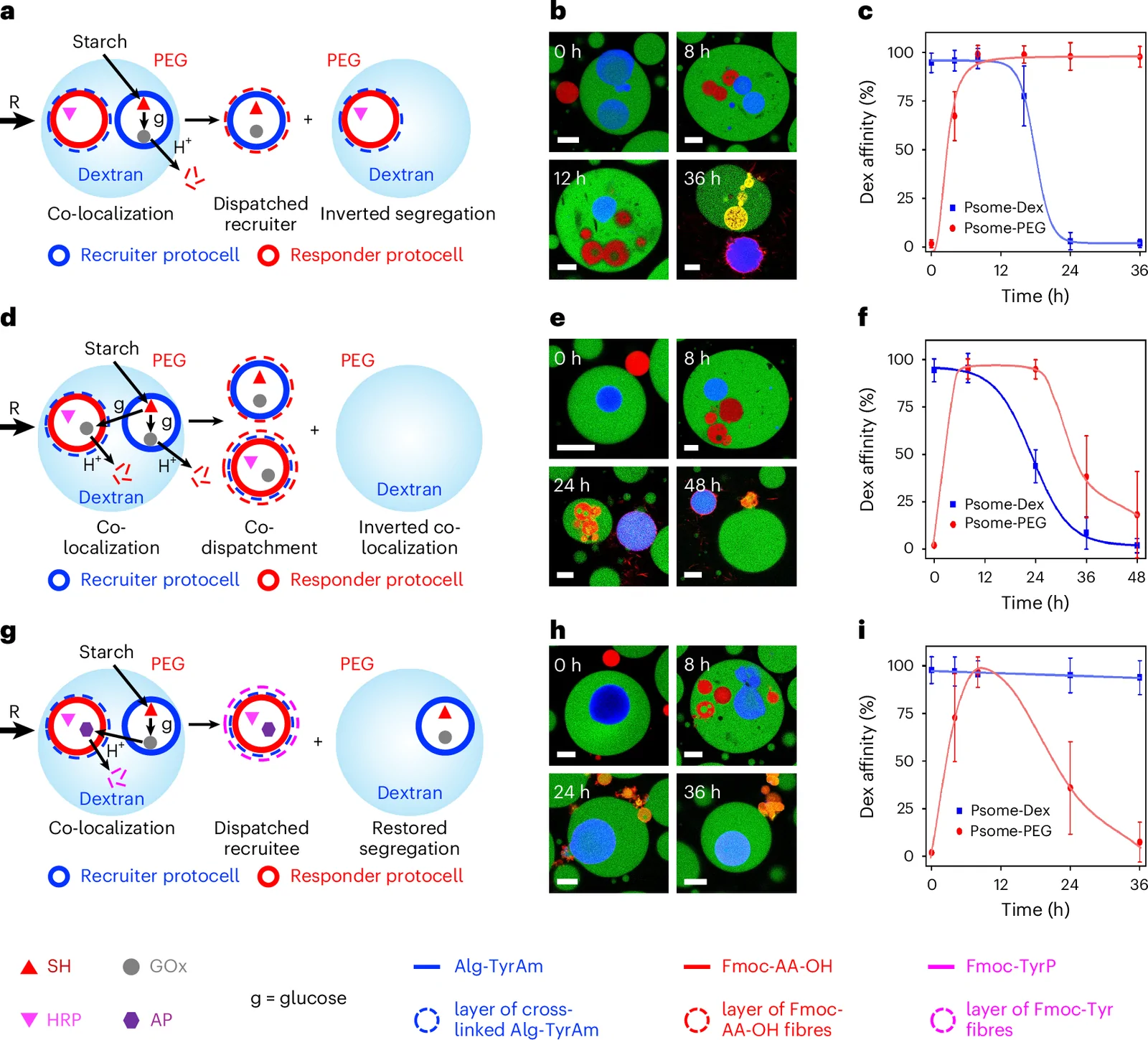
Selective dispatchment from phase-localized binary protocell populations. **a**–**i**, Three modes of post-recruitment selective dispatchment from dextran-droplet co-located binary proteinosome networks are shown: self-induced expulsion of recruiter protocells into the PEG phase (**a**–**c**); expulsion of both recruiter and responder protocells (**d**–**f**); and self-induced dispatchment of recruited protocells (**g**–**i**). Schematics (**a**, **d** and g): in each case, starch-initiated recruitment (R) of Psome-PEG responders (red open circles) into the dextran droplets (light-blue filled circles) by Psome-Dex protocells (blue open circles) is followed by acidification over prolonged periods and subsequent coating of targeted protocells with PEG-compatible peptide (**a** and **d**) or amino acid (**g**) fibrils. Images (**b**, **e** and **h**) of individual dextran droplets recorded before (0 h) and various times after a single addition of starch (50 mM) in the presence of Alg-TyrAm (1 mg ml^−1^) to initiate recruitment and Fmoc-AA-OH (2 mg ml^−1^ (**b** and **e**)) or Fmoc-TyrP (2 mg ml^−1^) (**h**)) fibre self-assembly to initiate dispatchment. The spatially segregated binary population (*t* = 0 h, pH 8.0 (**b**, **e** and **h**)) becomes co-localized in the dextran droplets by recruitment of the responders (*t* = 8 h, pH 7.0 (**b**, **e** and **h**); *t* = 12 h, pH 6.0 (**c**)), followed by selective dispatchment of the recruiter (*t* = 36 h, pH 5 (**c**)) or recruited protocells (*t* = 24, 36 h (**h**)), or sequential dispatchment of both the recruiter (*t* = 24 h) and recruited (*t* = 48 h) protocells (**e**). Fluorescence labels: dextran-in-PEG droplets (FITC, green); Dylight 405-Psome-Dex (blue); RITC-Psome-PEG (red). Yellow fluorescence in **b** arises from merging of the green (FITC-dextran) and red (RITC-responder protocells) fluorescence channels. Plots (**c**, **f** and **i**) of the percentage of dextran droplet-located recruiter and recruited protocells (dextran affinity) at different times after addition of starch; the resulting networks display inverted segregation (**c**), inverted co-localization (**f**) or restored segregation (**i**). Protocell number densities: SH/GOx-Psome-Dex, 0.6 × 10^7^ ml^−1^; HRP-, GOx/HRP- and HRP/AP-Psome-Dex, 1.2 × 10^7^ ml^−1^. Error bars: *n* (protocells) = 30 from ≥3 CLSM images at each timepoint, mean values ± s.d. in **c**, **f** and **i**. All scale bars, 20 µm. Source data

Having constructed protocell networks capable of actuating the spatial repositioning of selected proteinosomes, we developed three time-dependent enzyme-mediated strategies for the post-recruitment dispatchment of targeted protocells from phase-separated dextran-enriched droplets. In each case, endogenous enzyme activity within the protocells gave rise to co-localization of an initially spatially segregated binary population within the droplets, followed by selective protocell transfers to produce networks with inverted or restored segregation, or co-localization in the PEG phase (Supplementary Fig. 33). For example, selective dispatchment of SH/GOx/Psome-Dex recruiter protocells from dextran droplets containing recruited Alg-TyrAm-modified HRP/Psome-PEG constructs was achieved in the presence of Fmoc-AA-OH by prolonged SH/GOx production of gluconic acid and H_2_O_2_ after the single addition of an elevated starch concentration (50 mM) (Fig. 5a). The corresponding pH profiles showed a slow decrease from pH 8 to near-neutral values over 8 h, followed by continued acidification to pH 5 after 24 h (Supplementary Fig. 30), which were associated respectively with H_2_O_2_-induced Alg-TyrAm-modification of the responder Psome-PEG protocells and attachment of Fmoc-AA-OH fibres to the recruiter protocells. Consequently, recruitment of the responder protocells gave rise to a transient co-localized binary population that became progressively segregated over 12 h due to the selective dispatchment of peptide-modified recruiter proteinosomes into the PEG-enriched continuous phase (Fig. 5b and Supplementary Fig. 34). Plots of the corresponding time-dependent changes in protocell distribution indicated that the GOx-inactive polysaccharide-modified recruited protocells remained associated with the dextran droplets throughout the process of dipeptide assembly and that a segregated protocell network with an inverted spatial arrangement was established in the ATPS after 36 h (Fig. 5c).

Expulsion of both the co-localized recruiter and responder protocells from the dextran droplets to generate a co-localized network in the PEG phase was achieved by supplementing the HRP/Psome-PEG constructs with encapsulated GOx before Alg-TyrAm-modification and recruitment (Fig. 5d). Consequently, both populations in the dextran droplets were responsive to PEG-compatible changes in wettability when exposed to a GOx-induced acidic medium containing Fmoc-AA-OH. A time series of CLSM images and corresponding plots of the time-dependent changes in the percentage of the different protocell populations located within the dextran droplets indicated that recruitment in the presence of starch (50 mM), Alg-TyrAm and Fmoc-AA-OH occurred over 8 h at close-to-neutral pH and was followed by dispatchment of both protocell types as the pH progressively decreased over a further 36 h (Fig. 5e and Supplementary Fig. 35). Interestingly, the expulsion process was staggered in time with the peptide fibre-modified SH/GOx/Psome-Dex protocells being mostly dispatched before the peptide-modified Alg-TyrAm/HRP/GOx/Psome-PEG constructs (Fig. 5f). We attributed the relatively slow release of the recruited protocells to the presence of a pre-established dextran-compatible polysaccharide barrier layer, the possibility of further Alg-TyrAm cross-linking by on-going HRP activity, and the potential attenuation of the recruiter-derived diffusive glucose signal by primary consumption within the SH-containing proteinosomes.

Time-dependent signal-induced recruitment and subsequent dispatchment of the recruited responder protocells was achieved by supplementing the HRP/Psome-PEG constructs with acid phosphatase (AP) and exploiting the proton efflux arising from the starch-initiated activity of the SH/GOx/Psome-Dex recruiter protocells to switch on AP activity in the presence of the derivatized phosphorylated amino acid Fmoc-TyrP (Fig. 5g and Supplementary Fig. 11). A single input of starch (50 mM) in the presence of Alg-TyrAm and Fmoc-TyrP was used to recruit the Alg-TyrAm-coated responder protocells within 12 h, followed by their dispatchment over a period of 24 h to restore the spatially segregated protocell network (Fig. 5h,i and Supplementary Fig. 36). The results were consistent with the spatially confined AP-mediated dephosphorylation of Fmoc-TyrP and concomitant formation of a PEG-compatible layer of Fmoc-Tyr amino acid fibrils on the surface of the polysaccharide-coated recruited proteinosomes as the pH decreased below 6.0 (Supplementary Fig. 37).

**Fig. 6:**
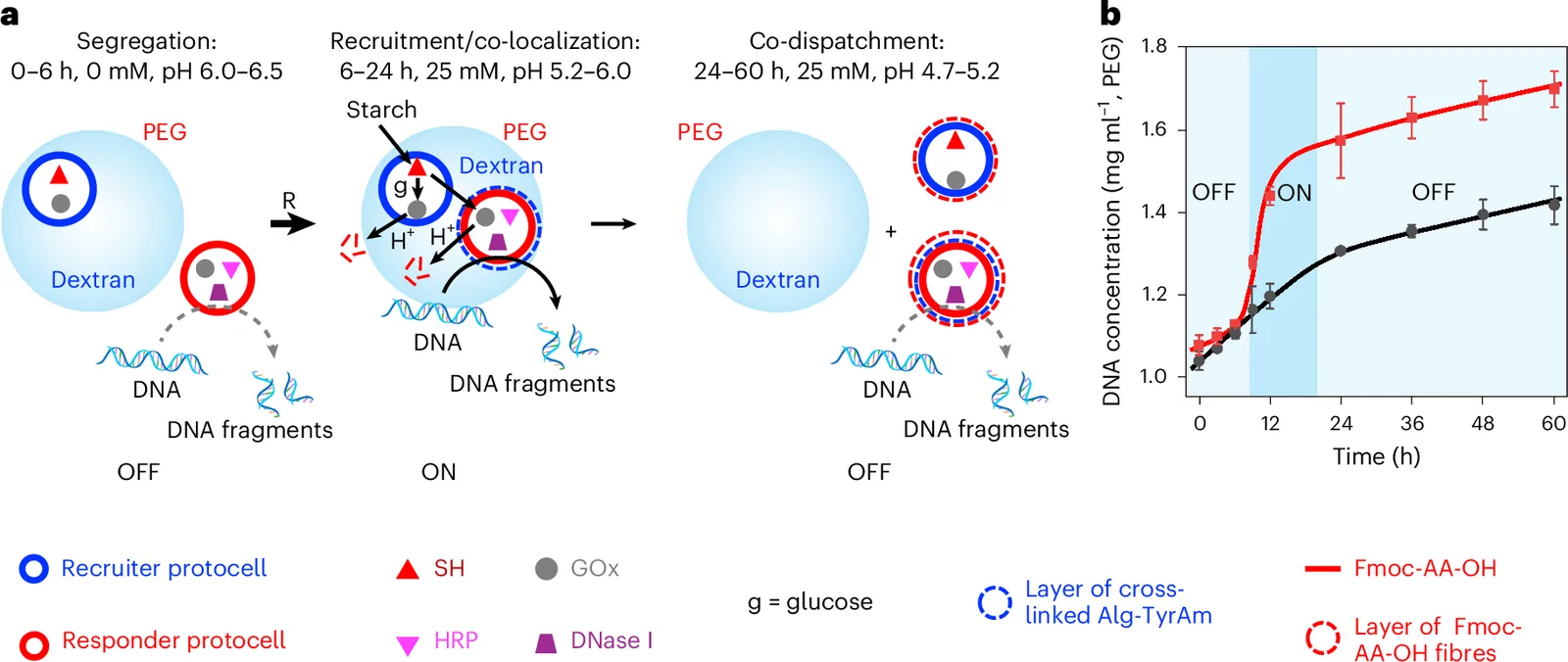
Recruitment/dispatchment-modulated DNase activity in phase-localized protocell populations. **a**, Schematic of a time-dependent signal-induced recruitment/dispatchment strategy for the spatiotemporal modulation of responder protocell-mediated DNase activity in an ATPS containing membrane-permeable dsDNA and a binary population of proteinosomes. Monitoring the changes in DNA concentration in the PEG phase over time was used as a proxy for DNase activity in the dextran droplets, which gave rise to PEG-compatible cleavage products. The rate of dsDNA cleavage is low (‘OFF’) before recruitment and after dispatchment of the responder protocells, and increases markedly (‘ON’) after recruitment into the dextran droplets (light-blue filled circles) and co-localization with the recruiter protocells. Starch (25 mM; *t* = 6 h) was added to initiate the recruitment process and subsequently drive the dispatchment mechanism. **b**, Corresponding time-dependent plots of dsDNA cleavage in the presence of Alg-TyrAm (1 mg ml^−1^) and Fmoc-AA-OH (2 mg ml^−1^) (red plot) or without Alg-TyrAm and Fmoc-AA-OH (black plot, control). The DNase activities associated with the successive time domains corresponding to initial segregation, recruitment-induced co-location and co-dispatchment of the binary population are labelled as OFF, ON and OFF, respectively. Protocell number densities: SH/GOx-Psome-Dex, 0.6 × 10^7^ ml^−1^; GOx/HRP/DNase-Psome-PEG, 1.2 × 10^7^ ml^−1^. MgCl_2_ (6 mM) in the ATPS was used to provide optimal DNase activities. Error bars: *n* = 3 (repeated groups of samples), mean values ± s.d. in **b**. Source data

We used a combination of signal-induced recruitment and dispatchment strategies in phase-separated media to spatiotemporally modulate the reaction kinetics associated with non-signalling enzyme activity within the responder protocells. As a proof of concept, we prepared DNase-loaded HRP-Psome-PEG protocells (Supplementary Fig. 38), mixed them with SH/GOx-Psome-Dex protocells and added the binary population to an ATPS containing Alg-TyrAm, Fmoc-AA-OH, MgCl_2_ and double-stranded DNA (dsDNA) (100–200 bp, MW 66–132 kDa). The dsDNA was distributed between the two phases with a Dex:PEG partition constant of 6.5 (Supplementary Fig. 39) and was spontaneously imported into the protocells (Supplementary Fig. 40). Addition of starch to the segregated protocell network gave rise to the endogenous production of H_2_O_2_ and gluconic acid within the dextran droplets; the former induced the coating of the Psome-PEG protocells over 6 h with Alg-TyrAm, while the latter resulted in a slow decrease in the pH to 5.3 over at least 12 h (Fig. 6a). Consequently, the Alg-TyrAm-coated Psome-PEG constructs were progressively recruited into the dextran droplets without loss of DNase activity (Supplementary Fig. 41). As higher levels of membrane-permeable DNA were present in the dextran phase, the DNase activity increased over a period of ~12 h compared with control experiments undertaken in the absence of Alg-TyrAm and Fmoc-AA-OH (Fig. 6b and Supplementary Fig. 42). Simultaneously, the slow acidification of the dextran droplets promoted the coating of the co-located recruiter and responder protocells with a peptide overlayer such that dispatchment of both protocell populations into the PEG phase gave rise to a concomitant decrease in DNA cleavage typically 18 h after the addition of starch (Fig. 6b). Thus, by endogenously orchestrating the proteinosome membrane modification processes at different time periods, it was possible to augment or attenuate DNase activity within a subpopulation of the protocell network via a self-promoted sequence of recruitment and dispatchment processes.

## Conclusions

Tuning the membrane wettability of different populations of enzyme-containing proteinosomes using dextran- or PEG-based membrane cross-linking agents provides a mechanism for implementing spontaneous spatial sorting and organization in segregated protocell networks distributed within aqueous dextran/PEG phase-separated media. Phase transfer and reversal of the segregated binary/ternary populations is subsequently achieved by in situ changes in dextran/PEG membrane wettability. Augmentation of the membrane is initiated by enzyme-mediated signalling from recruiter to responder protocells and involves fluxes of H_2_O_2_ and H^+^_._ The H_2_O_2_ signal induces Alg-TyrAm polymerization on the surface of the PEG-compatible responders, which renders the protocells dextran-compatible, while increasing acidification promotes coating of the dextran-compatible recruiters and Alg-Tyr-Am-modified responders with a surface layer of PEG-compatible Fmoc-AA-OH or Fmoc-Tyr fibrils. Consequently, direct, messenger-mediated and selective recruitment pathways can be implemented in segregated binary/ternary protocell populations to produce co-located communities within the dextran droplets that are subsequently resorted into reconfigured networks by selective dispatchment to the PEG continuous phase. In each case, the different induction times required to reach the signal threshold concentrations enables orthogonal recruitment and dispatchment pathways to be implemented. Unlike the peroxide signal, the acidification process is much slower and requires higher starch concentrations to attain the threshold pH values (<5.3) required for peptide fibre assembly. Thus, at low starch inputs (10 mM), the decrease in pH is below the threshold level, but the coproduced H_2_O_2_ signal is sufficient to activate HRP-mediated polymerization of Alg-TyrAm. Moreover, although both peptide-fibre and polysaccharide assembly occur at higher levels of starch (50 mM), the induction times are 6 and 12 h, respectively, enabling the recruitment and dispatchment pathways to be decoupled under the same input conditions.

Although a range of protocell-mediated recruitment and dispatchment strategies are possible, the intrinsic phase transfer dynamics are slow primarily due to the low rate of enzyme-mediated membrane coverage by Alg-TyrAm or dipeptide filaments. Other factors include the dependence on diffusive signalling and relatively infrequent droplet–protocell contact events especially under quiescent conditions. Currently, we rely on mechanical vortexing to increase the collision frequency, noting that mechanical agitation is not the driving force for protocell phase transfer, which occurs independent of droplet size or polydispersity within ~15 s once physical contact between the dextran droplets and coated proteinosomes is established. Moreover, although the vortexed dextran droplets typically range from 5 to 20 μm or 7 to 25 µm at 1,000 or 600 rpm (vortex time ~2 min), respectively, the size distributions are reproducible across different experiments, thereby facilitating the uniform distribution of enzyme-derived signalling molecules and reproducible time-dependent changes in membrane wettability and protocell phase transfer. Vortexing is also required when using higher concentrations of Fmoc-AA-OH because the peptide fibril-induced transfer events are attenuated by hydrogel formation in the continuous PEG phase that physically impedes protocell migration. In principle, microfluidic techniques could be used to generate monodisperse dextran droplets in PEG to increase the precision, reproducibility and generalizability of the methodology; however, given the extended time periods, coalescence of the as-produced dextran droplets could lead to comparable heterogeneity with a directly mixed system. Future work will seek to increase the robustness and long-term programmability of the methodology and establish increased levels of autonomous motion in the minimal networks. In the latter case, it may be feasible to use protocells capable of gas bubble-induced changes in buoyancy^19,39^, or self-propulsion using light-responsive gold nanoparticles^40^ or enzymes such as lipase^41^, which could induce thermal- or surfactant-driven Marangoni effects^42,43^. It should be possible to increase the predictive power of the methodology by quantification of the phase-interconnected reaction-diffusion gradients, and enhance the sensitivity and responsiveness of the protocell network by elevating enzyme, substrate and surface-modifier concentrations, shortening the signalling cascade by replacing starch with glucose and increasing the stability of the dextran droplets by addition of amphiphilic agents such as denatured BSA^44^.

Taken together, our work presents a chemical strategy for implementing programmable self-organization and signal-induced translocation in segregated protocell networks established in aqueous phase-separated media. The protocell phase dynamics can be spatiotemporally coupled to DNase I activity in a subpopulation of proteinosomes that undergo translocation in an ATPS containing partitioned dsDNA. Consequently, our approach highlights the potential to create protocell communities with self-regulated spatiotemporal order and offers a proof of concept towards the construction of cytomimetic networks with a degree of self-regulated autonomy and higher-order structure. In the longer term, the ability to construct protocell communities capable of ‘habitat’ selection, signal-induced migration and time-dependent relocation within alternative ‘niches’ by endogenous mechanisms of programmable chemical communication opens a pathway to the development of artificial ecosystems in which networks of protocells are intrinsically coupled to the dynamics of their local environment.

## Methods

### Preparation of NHS-activated dextran

NHS-activated dextran (Dex-NHS) was prepared by following a two-step procedure: (1) carboxylation of dextran^45^, and (2) 1-ethyl-3-(3-dimethylaminopropyl) carbodiimide (EDC)/NHS activation of the carboxylated dextran^46^. In step 1, dextran (2.5 g, 15.4 mmol; MW 40 kDa) and succinic anhydride (0.2 g, 2.0 mmol; MW 100.07 Da) were dissolved in 15 ml of dimethyl sulfoxide (DMSO) under stirring at 60 °C for 15 min, followed by addition of 300 µl of triethylamine to initiate an esterification reaction. The reaction mixture was then incubated at 60 °C and under stirring for 24 h. The generated carboxylated dextran (Dex-COOH) was purified by precipitating the cooled reaction mixture in 300 ml ethanol, storing the sample at −20 °C for 15 min and discarding supernatant after a centrifugation process (5,283*g*, 10 min). The collected pellet was then redissolved in 10 ml of DMSO, followed by repeated precipitation and removal of supernatant after centrifugation. Residual solvent was removed in a vacuum condition for over 6 h. In step 2, the carboxylated dextran (500 mg, ~1.2 mmol) was dissolved in 12 ml of DMSO by stirring at room temperature for 15 min, followed by an addition of EDC/NHS (100/100 mg) and an incubation process under stirring and at room temperature for over 24 h. The reaction product was collected by precipitating the reaction mixture in 200 ml of ethanol, storing the sample at −20 °C for 15 min and discarding supernatant after a centrifugation process (5,283*g*, 10 min). The sample was further purified by redissolving the collected pellet in 6 ml DMSO and repeating the procedures above. The purified NHS-activated dextran (Dex-NHS) was stored at 4 °C for usage.^1^H nuclear magnetic resonance spectra were collected to evaluate the generated carboxylated dextran and NHS-activated dextran. Samples were dissolved in DMSO-d_6_ at 15 mg ml^−1^.

### Preparation of tyramine-derivatized alginate

Tyramine-functionalized alginate (Alg-TyrAm) was prepared using an EDC/NHS-activated amination reaction^47^. In brief, 200 mg of sodium alginate and 500 mg of tyramine∙HCl were mixed and dissolved in 10 ml of MES buffer (0.2 M, pH 4.7). The reaction was then carried out by addition of 160/120 mg of EDC/NHS and incubation at room temperature and under stirring for 24 h. The generated Alg-TyrAm was purified by dialysis against deionized (DI) water for 3–4 days using a cellulose dialysis tube (Sigma, MWCO 12–14 kDa) and then lyophilization. The product was characterized using fourier transform infrared spectroscopy (4,000–550 cm^−1^, PerkinElmer) and ultraviolet–visible (UV–vis) spectroscopy (250–500 nm, Lambda 750, PerkinElmer). The content of TyrAm group (monomer ratio) was determined by using standard plots obtained from known concentrations of tyramine (0–0.6 mM).

As a test, the HRP-catalysed covalent cross-linking of the prepared Alg-TyrAm was implemented by addition of glucose (1,000 mM, 0 or 5 µl) to an aqueous solution (500 µl) containing dissolved Alg-TyrAm (1 mg ml^−1^), GOx (50 IU ml^−1^) and HRP (50 IU ml^−1^) and incubation under vortex for 12 h. The reaction mixtures were then characterized by recording the absorption at 250–500 nm using UV–vis spectroscopy.

### Selective partitioning of solutes in dextran/PEG phase-separated systems

An ATPS containing starch (Remazol Brilliant Blue R-stained, soluble Azure starch, monomer concentration 12.5, 25 or 50 mM) was prepared by adding different volumes of dissolved starch (500 mM; 10, 20 or 40 µl) to an aqueous mixture comprising dextran (250 mg ml^−1^, 51.2 µl) and PEG (250 mg ml^−1^, 153.6 µl), followed by addition of different amount of DI water and vortexing at 1,000 rpm for 30 s to establish a total volume of 400 µl. The PEG-rich and dextran-rich phases were separated by centrifuging the ATPS at 845*g* for 5 min and then separately diluted ten times using DI water. The concentrations of starch in the dextran and PEG phases were then quantitively determined by recording the absorption at 590 nm using UV–vis absorption spectroscopy (Lambda 750, PerkinElmer). Standard plots obtained from known Azure starch concentrations (0–10 mM) were used to calibrate the assay. Similar procedures were used to determine the partition constants for fluorescein isothiocyanate (FITC)-labelled alginate (140–160 kDa, 5 mg ml^−1^, 20 µl), dsDNA (salmon sperm, low MW, 100–200 bp, 5 mg ml^−1^, 20 µl), Fmoc-AA-OH (25 mg ml^−1^, 20 µl)) and glucose (500 mM, 5–20 µl) in dextran/PEG ATPSs (250 mg ml^−1^, 25.6 µl), PEG (250 mg ml^−1^, 76.8 µl). The mixtures were made up to 200 µl using DI water, vortexed and then centrifuged to separate the PEG and dextran phases. Solute concentrations were determined by UV–vis spectroscopy using absorption values recorded at 491 nm (alginate), 260 nm (dsDNA) and 299 nm (Fmoc-AA-OH). Glucose concentrations were determined using a GOx/HRP/*o*-PD assay^10^. In brief, the separated PEG-rich and dextran-rich solutions (10 µl) were diluted to 100 µl using DI water, followed by addition of 100 µl *o*-PD (100 mM), 100 µl GOx (10,000 IU ml^−1^), 100 µl HRP (200 IU ml^−1^) and 400 µl PBS buffer (pH 6.0) and incubation at 37 °C for 30 min. The production of 2,3-diaminophenazine (2,3-DAP) from the GOx/HRP enzyme cascade was stopped by addition of 200 µl HCl solution (5 M), followed by fivefold dilution. The 2,3-DAP absorbance at 490 nm was used to determine the glucose concentrations. Standard plots obtained from known glucose concentrations (0–0.2 mM) were used to calibrate the GOx/HRP assay.

### Preparation of PEG/dextran-grafted proteinosomes

Populations of proteinosomes were prepared as reported previously^38^ by interfacial self-assembly and cross-linking of BSA/PNIPAAm nanoconjugates at an oil–water droplet interface. Two different cross-linkers, NHS-activated PEG (PEG-NHS) or NHS-activated dextran (Dex-NHS), were used to modify the membrane wettability to produce Psome-PEG or Psome-Dex protocells, respectively. Typically, the Psome-PEG protocells were generated by blending an aqueous mixture (60 µl) comprising BSA/PNIPAAm nanoconjugates (FITC-labelled, 30 mg ml^−1^, 10 µl), carbonate buffer (Na_2_CO_3_/NaHCO_3_, 100/100 mM, 10 µl), H_2_O (20 µl) and dissolved PEG-NHS (cross-linker, 2.5 mg, 20 µl) with 1000 µl of 2-ethyl-1-hexanol (oil phase) under mechanical stirring to generate a water droplet-in-oil emulsion. After incubation at 4 °C for 2 days, the proteinosomes were transferred into water by stepwise dialysis against 1000 ml of 70%, 40%, 20%, 0% ethanol/water solutions (v/v) using cellulose dialysis tubes (Sigma, MWCO 12–14 kDa). The purified aqueous suspension of Psome-PEG (~3 ml) was collected and stored at 4 °C. Centrifugation at 2,348*g* for 5 min was used to produce concentrated populations of the PEG-grafted proteinosomes. Similarly, Psome-Dex protocells were prepared by mixing dissolved Dex-NHS (cross-linker, 2.5 mg, 20 μl) with an aqueous solution (40 µl) comprising BSA/PNIPAAm nanoconjugates (Dylight 405-labelled, 30 mg ml^−1^, 15 µl), carbonate buffer (Na_2_CO_3_/NaHCO_3_, 100/100 mM, 10 µl), H_2_O (15 µl), followed by addition of 1000 µl of 2-ethyl-1-hexanol (oil phase) and emulsification under vortex. The generated proteinosomes were transferred into water and purified as described above. Aqueous suspensions of Psome-Dex were ~4 ml in volume and stored at 4 °C overnight, during which sedimentation occurred. Concentrated populations of Psome-Dex were obtained by removal of the supernatant. Psome-PEG/Dex protocells were prepared using both PEG-NHS and Dex-NHS (1.25 mg PEG-NHS + 1.25 mg Dex-NHS, 20 µl) cross-linkers at the same time to cross-link the self-assembled BSA/PNIPAAm nanoconjugates (rhodamine isothiocyanate (RITC)-labelled).

The surface charge on the proteinosomes was determined by Zeta potential measurements (Malvern Instruments). Protocells were imaged using a Leica DMI3000 B fluorescence microscope (Leica) and/or a SP5-II confocal laser scanning microscope (Leica). The obtained images were analysed using ImageJ software.

### Selective localization of protocells in phase-separated media

Psome-PEG, Psome-PEG/Dex and Psome-Dex constructs were separately transferred into a dextran-in-PEG ATPS by blending a stocked suspension of proteinosomes (50 µl) with an aqueous mixture consisting of dextran (250 mg ml^−1^, 32 µl), PEG (250 mg ml^−1^, 96 µl) and DI water (52 µl) under mechanical vortex (1,000 rpm, 5–10 s). The resulting mixtures were characterized using a SP5-II confocal laser scanning microscope (Leica) and analysed with Image J software. The spatial organization of proteinosomes in the ATPS was statistically evaluated by defining an index, termed dextran affinity, which was calculated by using the following equation:

$${A}_{D}({\rm{dextran}}\,{\rm{affinity}},\, \% )=\frac{{L}_{{Dex}}}{{L}_{{Dex}}+\,{L}_{{PEG}}}\,\times 100 \%$$

where *L*_Dex_ and *L*_PEG_ are lengths of perimeter of a proteinosome contacting with the dextran-rich droplets and continuous PEG-rich phase, respectively, which were measured using ImageJ software.

### Segregation of binary populations of proteinosomes in aqueous phase-separated media

A mixed population of Psome-PEG (number density, 9.2 × 10^7^ ml^−1^; 25 µl) and Psome-Dex (5.6 × 10^7^ ml^−1^; 25 µl) was transferred into an aqueous solution comprising dextran (250 mg ml^−1^, 32 µl), PEG (250 mg ml^−1^, 96 µl) and DI water (52 µl) and mechanically vortexed (1,000 rpm, 5–10 s) to produce an ATPS (final dextran concentration, 34.8 mg ml^−1^, final PEG concentration, 104.3 mg ml^−1^). Spontaneous segregation of the Psome-PEG/Psome-Dex mixture under quiescent conditions was monitored by recording CLSM images over time. Samples were characterized using a CLSM and analysed with ImageJ software.

### Enzyme-mediated surface modification of Psome-PEG in aqueous solution

Aqueous suspensions of RITC-labelled Psome-PEG protocells containing SH (amylase/amyloglucosidase), GOx and HRP (2/2/2/2 mg ml^−1^) were prepared as described above. Gelatin (6 mg ml^−1^) was sometimes included in the experiments to increase the robustness of the protocells. Proteinosomes (number density 13.8 × 10^7^ ml^−1^, 30 µl) were then transferred into aqueous solutions of Alg-TyrAm (40 mg ml^−1^, 3–12 µl; FITC-labelled or non-labelled) and carbonate buffer (Na_2_CO_3_/NaHCO_3_, 100/100 mM, 5 µl), and then glucose (500 mM, 4 µl) or dissolved starch (500 mM, 10 µl) added. The reaction mixtures were made up to 230 µl by adding DI water. The amount of Alg-TyrAm covalently grafted to the proteinosomes was evaluated by recording the absorption at 275 nm of residual dissolved Alg-TyrAm present in the supernatant obtained after centrifugation (13,523*g*, 2 min) of the reaction mixture. Standard plots obtained from the known concentrations of Alg-TyrAm (0–2 mg ml^−1^) were used to calibrate the assay. The surface-modified Psome-PEG (1.8 × 10^7^ ml^−1^, 10 µl) was then added to a dextran-in-PEG ATPS (50 µl, dextran 34.7 mg ml^−1^, PEG 104.3 mg ml^−1^), sealed in a glass channel and monitored in situ using CLSM.

### Enzyme-mediated membrane modification of protocells in aqueous phase-separated media

#### Alg-TyrAm coating of Psome-PEG

RITC-labelled Psome-PEG protocells containing SH (amylase/amyloglucosidase)/GOx/HRP/gelatin (0/0/2/2/6 or 2/2/2/2/6 mg ml^−1^; number density 13.8 × 10^7^ ml^−1^; 30 µl) were transferred into an ATPS comprising dextran (250 mg ml^−1^, 32 µl), PEG (250 mg ml^−1^, 96 µl), dissolved Alg-TyrAm (40 mg ml^−1^, 0–12 µl) and carbonate buffer (Na_2_CO_3_/NaHCO_3_, 100/100 mM, 5 µl) and activated by adding glucose (500 mM, 0–23 µl). The reaction mixtures were made up to 230 µl by adding DI water, followed by vortexing at room temperature for 24 h. Images of the coated protocells were recorded using CLSM. The dextran affinity was statistically calculated by analysing the recorded images using Image J software. Changes in pH were recorded using a pH meter (METTLER TOLEDO).

#### Fmoc-AA-OH coating of Psome-Dex

Dylight 405-labelled Psome-Dex containing SH (amylase/amyloglucosidase)/GOx (2/2/2 mg ml^−1^); number density (3.5 × 10^7^ ml^−1^, 40 µl) were transferred into an ATPS comprising dextran (250 mg ml^−1^, 32 µl), PEG (250 mg ml^−1^, 96 µl), carbonate buffer 8.5 (Na_2_CO_3_/NaHCO_3_, 100/100 mM, 5 µl), dissolved Fmoc-AA-OH (50 mg ml^−1^, 0–14 µl) and Nile red (0.02 mg ml^−1^, 3 µl), and then activated by adding glucose (500 mM, 0–20 µl) or starch (500 mM, 20 µl). The reaction mixtures were then processed as described above. Changes in pH were recorded using a pH meter (METTLER TOLEDO).

### Cyclic phase transfer of Psome-Dex in aqueous phase-separated media

A binary population of proteinosomes (SH/GOx-loaded Psome-Dex, density 3.5 × 10^7^ ml^−1^, 40 µl; urease-loaded Psome-PEG, density 13.8 × 10^7^ ml^−1^, 30 µl) were produced and transferred into an aqueous mixture comprising dextran (250 mg ml^−1^, 32 µl), PEG (250 mg ml^−1^, 96 µl), carbonate buffer 8.5 (Na_2_CO_3_/NaHCO_3_, 100/100 mM, 5 µl) and dissolved Fmoc-AA-OH (50 mg ml^−1^, 10 µl). The reaction mixture was then activated by adding starch (500 mM, 23 µl) and incubated under vortex for over 48 h, followed by addition of urea (500 mM, 2.5 µl) at 24 and 48 h. Changes in pH were monitored using a pH meter. The self-assembly of Fmoc-AA-OH was quantitatively evaluated by recording the absorption at 299 nm using UV–vis spectroscopy. The spatial organization of protocells in ATPS was recorded over time by CLSM and analysed using ImageJ software.

### Protocell-mediated signal-induced recruitment of responder protocells in aqueous phase-separated media

Three interrelated strategies were designed to recruit the Psome-PEG protocells from the PEG phase by enzyme activity in Psome-Dex protocells housed in the dextran-rich droplets of an ATPS. A binary population of SH/GOx-containing Psome-Dex (recruiter, density 3.5 × 10^7^ ml^−1^, 40 µl) and HRP-Psome-PEG (responder, 13.8 × 10^7^ ml^−1^, 20 µl) (mode 1); a ternary population of SH-loaded Psome-Dex (recruiter, density 3.5 × 10^7^ ml^−1^, 30 µl), GOx-loaded Psome-PEG (messenger, density 13.8 × 10^7^ ml^−1^, 10 µl) and HRP-loaded Psome-PEG (responder, density 13.8 × 10^7^ ml^−1^, 10 µl) (mode 2); or a ternary population of protocells comprising SH/GOx-loaded Psome-Dex (recruiter, density 3.5 × 10^7^ ml^−1^, 30 µl), enzyme-inactive Psome-PEG (non-responder, density 13.8 × 10^7^ ml^−1^, 10 µl) and HRP-loaded Psome-PEG (responder, density 13.8 × 10^7 ^ml^−1^, 10 µl) (mode 3) were separately transferred into a mixture comprising dextran (250 mg ml^−1^, 32 µl), PEG (250 mg ml^−1^, 96 µl), carbonate buffer 8.0 (Na_2_CO_3_/NaHCO_3_, 100/100 mM, 2 µl), Nile red (0.02 mg ml^−1^, 3 µl), dissolved Alg-TyrAm (40 mg ml^−1^, 6 µl), Fmoc-AA-OH (50 mg ml^−1^, 10 µl) and starch (500 mM, 4.6 µl). The reaction mixtures were made up to 230 µl by adding DI water, followed by incubation at room temperature under vortex for 24 h. Changes in pH were recorded using a pH meter (METTLER TOLEDO). Fluorescence images were recorded at different times (for example, 0, 4, 8, 12 and 24 h) by CLSM and then analysed using ImageJ software. Surface modifications of proteinosomes were quantitatively evaluated by calculating the percentage of Alg-TyrAm or Fmoc-AA-OH deposited on the Psome-PEG or Psome-Dex protocells as described above.

### Protocell-mediated signal-induced dispatchment of selective protocells in aqueous phase-separated media

Three modes of post-recruitment selective dispatchment of protocells were implemented.

#### Expulsion of recruiter protocells into the PEG phase (mode 1)

A binary protocell population consisting of Dylight 405-labelled Psome-Dex containing SH (amylase/amyloglucosidase)/GOx (2/2/2 mg ml^−1^) and RITC-labelled Psome-PEG loaded with HRP/gelatin (2/6 mg ml^−1^) were transferred (Psome-Dex, 3.5 × 10^7^ ml^−1^, 40 µl; Psome-PEG, 13.8 × 10^7 ^ml^−1^, 20 µl) into an aqueous solution comprising dextran (250 mg ml^−1^, 32 µl), PEG (250 mg ml^−1^, 96 µl), carbonate buffer 8.0 (Na_2_CO_3_/NaHCO_3_, 100/100 mM, 2 µl), Nile red (0.02 mg ml^−1^, 3 µl), dissolved Alg-TyrAm (40 mg ml^−1^, 6 µl), Fmoc-AA-OH (50 mg ml^−1^, 10 µl) and dissolved starch (500 mM, 23 µl). The reaction mixture was made up to a final volume of 230 µl by adding DI water and then incubated at room temperature under vortex for 36 h. The pH was monitored and CLSM images recorded over time to determine the changes in dextran affinity of the different protocells.

#### Expulsion of both recruiter and responder protocells (mode 2)

The above procedures were used to prepare a binary population consisting of SH (amylase/amyloglucosidase)/GOx (2/2/2 mg ml^−1^)-containing Psome-Dex and GOx/HRP/gelatin-loaded RITC-labelled Psome-PEG (2/2/6 mg ml^−1^). Reaction mixtures were activated, processed and analysed following the steps in mode 1 (vortex, 48 h).

#### Expulsion of recruited protocells (mode 3)

The above procedures were used to prepare a binary population consisting of SH (amylase/amyloglucosidase)/GOx (2/2/2 mg ml^−1^)-containing Psome-Dex and AP/HRP/gelatin-loaded RITC-labelled Psome-PEG (2/2/6 mg ml^−1^). Fmoc-TyrP (50 mg ml^−1^, 25 µl) was used in place of Fmoc-AA-OH and starch volumes were 5 or 25 µl. Reaction mixtures were activated, processed and analysed following the steps in mode 1.

AP-induced dephosphorylation of soluble Fmoc-TyP was monitored by UV–vis spectroscopy (Lambda 750, PerkinElmer). Ten microlitres of the reaction mixtures were collected and then diluted to 200 µl using MES buffer 4.5 (0.2 M). Fmoc-Tyr fibres were then removed by centrifugation (13,523*g*, 2 min) and the supernatants (50 µl) collected and further diluted to 500 µl using DI water. The concentrations of soluble Fmoc-TyrP at different time intervals were quantitatively determined by recording the absorption values at 299 nm. Standard plots obtained from known concentrations of Fmoc-TyrP (0–0.04 mg ml^−1^) were used to calibrate the assay.

### Protocell recruitment/dispatchment-mediated modulation of DNase activity in aqueous phase-separated media

Dylight 405-labelled Psome-Dex containing SH (amylase/amyloglucosidase) and GOx (3.5 × 10^7^ ml^−1^, 40 µl, 2/2/2 mg ml^−1^) and RITC-labelled Psome-PEG loaded with GOx, HRP and DNase I (13.8 × 10^7^ ml^−1^, 20 µl, 2/2/2 mg ml^−1^) were added to an aqueous solution comprising dextran (250 mg ml^−1^, 32 µl), PEG (250 mg ml^−1^, 96 µl), Bis-Tris buffer (0.4 M, pH 6.5, 20 µl), Alg-TyrAm (40 mg ml^−1^, 0 or 6 µl), Fmoc-AA-OH (50 mg ml^−1^, 0 or 10 µl) and MgCl_2_ (0.4 M, 3.5 µl). Dissolved dsDNA (50 mg ml^−1^, 10 µl) was then added to the ATPS, and the final volume made up to 240 µl with DI water. The reaction mixtures were incubated under vortex (600 rpm) at room temperature for 6 h in the absence of starch. Starch (500 mM, 12.5 µl) was then added and the ATPS stirred at room temperature under vortex for 2–3 days to induce phase transfer of the Psome-PEG protocells into and out of the dextran droplets. Changes in pH value were recorded and dsDNA cleavage quantitatively evaluated by recording the time-dependent increases in DNA concentration (UV–vis absorption values at 260 nm and 299 nm) in the continuous PEG-rich phase associated with the increase in PEG-compatible cleavage products. Given the different linear ratios of absorption values (A260/A299) of dsDNA (17.5 ± 0.4) and Fmoc-AA-OH peptide (3.1 ± 0.03), the UV absorption of DNA at 260 nm (A260, DNA) was determined by removing the absorption value of dissolved Fmoc-AA-OH according to: A260, DNA = 1.24 × (A260 – 4.61 × A299), where A260, DNA is the UV absorption value of DNA at 260 nm, and A260 and A299 are the UV absorption values of the whole reaction mixture at 260 nm and 299 nm, respectively. Standard plots obtained from known concentrations (0–0.03 mg ml^−1^) of dsDNA were used to calibrate the assay.

### Reporting summary

Further information on research design is available in the Nature Portfolio Reporting Summary linked to this article.

## Online content

Any methods, additional references, Nature Portfolio reporting summaries, source data, extended data, supplementary information, acknowledgements, peer review information; details of author contributions and competing interests; and statements of data and code availability are available at https://doi.org/10.1038/s41557-026-02225-3.

## Supplementary information

Supplementary Information Supplementary Materials and Methods, Refs. 1–3 and Figs. 1–42.

Reporting Summary

## Source data

Source Data Fig. 1 Statistical source data.

Source Data Fig. 2 Statistical source data.

Source Data Fig. 3 Statistical source data.

Source Data Fig. 4 Statistical source data.

Source Data Fig. 5 Statistical source data.

Source Data Fig. 6 Statistical source data.

## Data Availability

All data needed to evaluate the conclusions in the study are present in the Article and/or its Supplementary Information. Source data are provided with this paper.
