# Supplementary material for: Signal-induced recruitment and dispatchment in reconfigurable phase-separated protocell networks
Source: Nat Chem. 2026 Aug 18;18(10):1828–37. doi: 10.1038/s41557-026-02225-3 (PMC13623635; doi:10.1038/s41557-026-02225-3)
Supplement: Supplementary file 1 [file 41557_2026_2225_MOESM1_ESM.pdf]

nature chemistry

Supplementary information

https://doi.org/10.1038/s41557-026-02225-3

Signal-induced recruitment and dispatchment in reconfigurable phase-separated protocell networks

In the format provided by the authors and unedited

Contents

1. Supplementary Materials and Methods 2
2. References 4
3. Supplementary Figures 5

1. Supplementary Materials and Methods

Materials

The following chemicals were used as received: dextran (Mw = 40 kD, Sigma-Aldrich), FITC-labelled dextran (Mw = 40 kD, Sigma-Aldrich), PEG (Mw = 35 kD, Sigma-Aldrich), sodium alginate (Mw = 140-160 kD, Sigma-Aldrich), deoxyribonucleic acid (dsDNA, low molecular weight, ca. 100-200 bp, from salmon sperm, Sigma-Aldrich), starch (soluble, from potato, Sigma-Aldrich), succinic anhydride (Sigma-Aldrich), triethylamine (Sigma-Aldrich), 1-ethyl-3-(3-dimethylaminopropyl) carbodiimide (EDC, Thermo Fisher Scientific), N-Hydroxysuccinimide (NHS, Sigma-Aldrich), Bovine Serum Albumin (BSA, Sigma-Aldrich), 2-ethyl-1-hexanol (Sigma-Aldrich), O,O′-bis[2-(N-succinimidyl-succinylamino)ethyl]polyethylene glycol (PEG-NHS, Mw = 2000, Sigma-Aldrich), tyramine hydrochloride (Acros Organics, Thermo Fisher Scientific), fluorescein isothiocyanate isomer I (FITC, ≥ 90%, Sigma-Aldrich), rhodamine B isothiocyanate mixed isomers (RITC, Sigma-Aldrich), DyLight 405 NHS Ester (Thermo Fisher Scientific), D-(+)-glucose (≥ 99.5%, Sigma-Aldrich), urea (BioReagent, Sigma-Aldrich), N-[(9H-fluoren-9-ylmethoxy)carbonyl]-L-alanyl-(2S)-2-[[(2S)-2-(9H-fluoren-9-ylmethoxy carbonylamino)propanoyl]amino]propanoic acid (Fmoc-Ala-Ala-OH; Insight Biotechnology), N-alpha-(9-Fluorenylmethyloxycarbonyl)-L-phosphotyrosine (Fmoc-TyrP, Sigma-Aldrich), α-amylase (from Aspergillus oryzae, Sigma-Aldrich), amyloglucosidase (ca. 120 U/mg, from Aspergillus niger, Sigma-Aldrich), glucose oxidase (GOx, 100 - 250 U/mg, from Aspergillus niger, Sigma-Aldrich), peroxidase from horseradish (HRP, ca. 150 U/mg, Sigma-Aldrich), urease (ca. 8 U/mg, from Canavalia ensiformis, Sigma-Aldrich), deoxyribonuclease I (≥ 2000 U/mg protein, from bovine pancreas, Sigma-Aldrich), acid phosphatase (≥ 3 U/mg, from potato, Sigma-Aldrich).

Fmoc-Ala-Ala-OH self-assembly

Fmoc-Ala-Ala-OH self-assembly into supramolecular nanofilaments in acidic conditions by hydrophobic interactions and π-π stacking[1]. 10 µl of mixtures containing Fmoc-AA-OH (initial concentration 2.5 mg/ml, pH value = 4 - 8) were collected 24 h after pH adjustment and diluted to 100 µl using DI water, followed by centrifugation (12000 rpm, 2 min) to remove the peptide fibrils. The supernatants (50 µl) were diluted to 500 µl using PBS 6.5 (0.2 M), and the concentrations of residual Fmoc-AA-OH monomers determined by measuring the UV/Vis absorption at values at 299 nm. Standard plots obtained from known peptide concentrations (0-0.04 mg/ml) were used to calibrate the assay.

DNA cleavage in aqueous phase-separated media

Deoxyribonuclease I (DNase I)[2] was used to degrade dsDNA (ca. 100-200 bp, salmon sperm) within an ATPS. Typically, aqueous mixtures comprising dextran (250 mg/ml, 32 µl), PEG (250 mg/ml, 96 µl), PBS 7.0 (0.2 M, 20 µl), $MgCl_2$ (200 mM, 6 µl) and dsDNA (20 mg/ml, 23 µl) were prepared and then different amount of DNase I (2000 U/ml, 0 or 6 µl) added. The reaction mixtures were made up to 230 µl in volume by adding DI water and then incubated at room temperature and under vortex for 24 hours. The

dextran and PEG phases were isolated by centrifuging at 3000 rpm for 5 min. The collected PEG and dextran phases (10 µl) were diluted to 1000 µl using DI water and the concentration of DNA in each phase determined by recording the absorption at 260 nm using UV/VIS spectroscopy (Lambda 750, PerkinElmer, USA). Standard plots obtained from known concentrations (0 - 0.03 mg/ml) of dsDNA were used to calibrate the assay.

Labelling of BSA/PNIPAAm nanoconjugates (proteinosome membrane)

BSA/PNIPAAm nanoconjugates were produced according to a previous report[3] (ref. 38: Huang, X. et al. Nat. Commun. 4, 2239 (2013)) by reaction between 1,6-hexanediamine-cationized BSA (Mw: ca. 66 kD) with end-capped mercaptothiazoline-activated PNIPAAm (Mw: ca. 8 kD) in alkaline conditions. The obtained BSA/PNIPAAm nanoconjugates were fluorescently labelled by introducing amine-reactive dyes containing either isothiocyanate or N-hydroxysuccinimide (NHS) ester functional groups, including fluorescein isothiocyanate isomer I (FITC), rhodamine B isothiocyanate mixed isomers (RITC) or DyLight 405 NHS ester. Typically, a fluorescence dye solution (FITC, RITC or Dylight 405 NHS ester; 2 mg/ml in DMSO; 50 µl) was added to an aqueous mixture comprising BSA/PNIPAAm nanoconjugates (2 mg/ml, 5 ml) and carbonate buffer ($NaHCO_3/Na_2CO_3$, 100/100 mM, pH 8.5, 5 ml). The reaction was performed under stirring and at 4 ^oC for > 12 h. The reaction products were purified by ultrafiltration against DI water (4 ^oC) using a cellulose membrane filter (MWCO: ~ 50 kD) for 3-4 times, lyophilized and stored at -20 ^oC until usage.

Preparation of enzyme-loaded proteinosomes

An aqueous suspension of RITC-labelled Psome-PEG protocells containing starch hydrolase (α-amylase/amyloglucosidase), glucose oxidase (GOx), horseradish peroxidase (HRP), and gelatin (final concentrations: 2/2/2/2/6 mg/ml) was prepared by mixing an aqueous solution comprising BSA/PNIPAAm nanoconjugates (RITC-labelled, 30 mg/ml, 10 µl), carbonate buffer ($Na_2CO_3/NaHCO_3$, 100/100 mM, 10 µl), α-amylase (40 mg/ml, 3 µl), amyloglucosidase (40 mg/ml, 3 µl), GOx (40 mg/ml, 3 µl), HRP (40 mg/ml, 3 µl), gelatin (60 mg/ml, 10 µl), and dissolved PEG-NHS (cross-linker, 2.5 mg in 15 µl H_2O). The mixture was combined with 1000 µl of 2-ethyl-1-hexanol (oil phase) under mechanical stirring to generate a water-in-oil emulsion. After quiescent incubation at 4 °C for 2 days, the sedimented emulsion droplets were transferred into aqueous media by stepwise dialysis against ethanol/water solutions (70%, 40%, 20%, and 0%) and subsequently concentrated by centrifugation (5000 rpm, 5 min), as described above."

An aqueous dispersion of DyLight 405-labelled Psome-Dex protocells containing starch hydrolase (α-amylase/amyloglucosidase) and GOx (final concentrations: 2/2/2 mg/ml) was prepared by mixing an aqueous solution comprising BSA/PNIPAAm nanoconjugates (RITC-labelled, 30 mg/ml, 15 µl), carbonate buffer ($Na_2CO_3/NaHCO_3$, 100/100 mM, 10 µl), α-amylase (40 mg/ml, 3 µl), amyloglucosidase (40 mg/ml, 3 µl), HRP (40 mg/ml, 3 µl), and dissolved Dex-NHS (cross-linker, 2.5 mg in 26 µl H_2O). The mixture was emulsified with 1000 µl of 2-ethyl-1-hexanol under mechanical stirring to form a water-in-oil emulsion. After quiescent incubation at 4 °C for 2 days, the emulsion droplets were transferred into aqueous media

by stepwise dialysis against ethanol/water solutions (70%, 40%, 20%, and 0%) and concentrated by static sedimentation to remove the supernatant.

Hydrogen peroxide assays

Starch hydrolase/GOx-loaded Psome-Dex + HRP-loaded Psome-PEG: A binary population of DyLight-405-Psome-Dex (SH/GOx, 2/2/2 mg/ml; 3.5 × 10^7/ml, 40 µL) and RITC-Psome-PEG (HRP/gelatin, 2/6 mg/ml; 13.8 × 10^7/ml, 20 µl) was added to dextran (250 mg/mL, 32 µl), PEG (250 mg/ml, 96 µl), carbonate buffer pH 8.0 (Na_2CO_3/$NaHCO_3$, 100/100 mM, 2 µl) and dissolved starch (500 mM, 4.6 or 23 µl). DI water was added to 230 µl and samples were vortex-incubated at RT for 36 h. H_2O_2 concentration were then determined by a HRP/ABTS assay. Aliquots (10 µl) were taken at 0, 2, 4, 8 and 12 h, diluted to 100 µl with DI water and centrifuged (12,000 rpm, 2 min). H_2O_2 levels were determined by HRP/ABTS assay (A_{420}). Supernatants (50 µl) were added to HRP (~ 20 U/ml)/ABTS (~ 20 mM) solution (850 µl), incubated at 37 °C for 2 h and quenched with SDS (100 µl, 10%, w/v). Standard plots obtained from known concentrations of H_2O_2 (0-0.02 mM) were used to calibrate the assay.

2. References

1. Rodon Fores, J. et al. Localized supramolecular peptide self-assembly directed by enzyme-induced proton gradients. Angewandte Chemie 129, 16200-16204 (2017).
2. Fojta, M., Kubičárová, T. & Paleček, E. Cleavage of supercoiled DNA by deoxyribonuclease I in solution and at the electrode surface. Electroanalysis: An International Journal Devoted to Fundamental and Practical Aspects of Electroanalysis 11, 1005-1012 (1999).
3. Huang, X. et al. Interfacial assembly of protein–polymer nano-conjugates into stimulus-responsive biomimetic protocells. Nature Communications 4, 2239 (2013).

3. Supplementary Figures

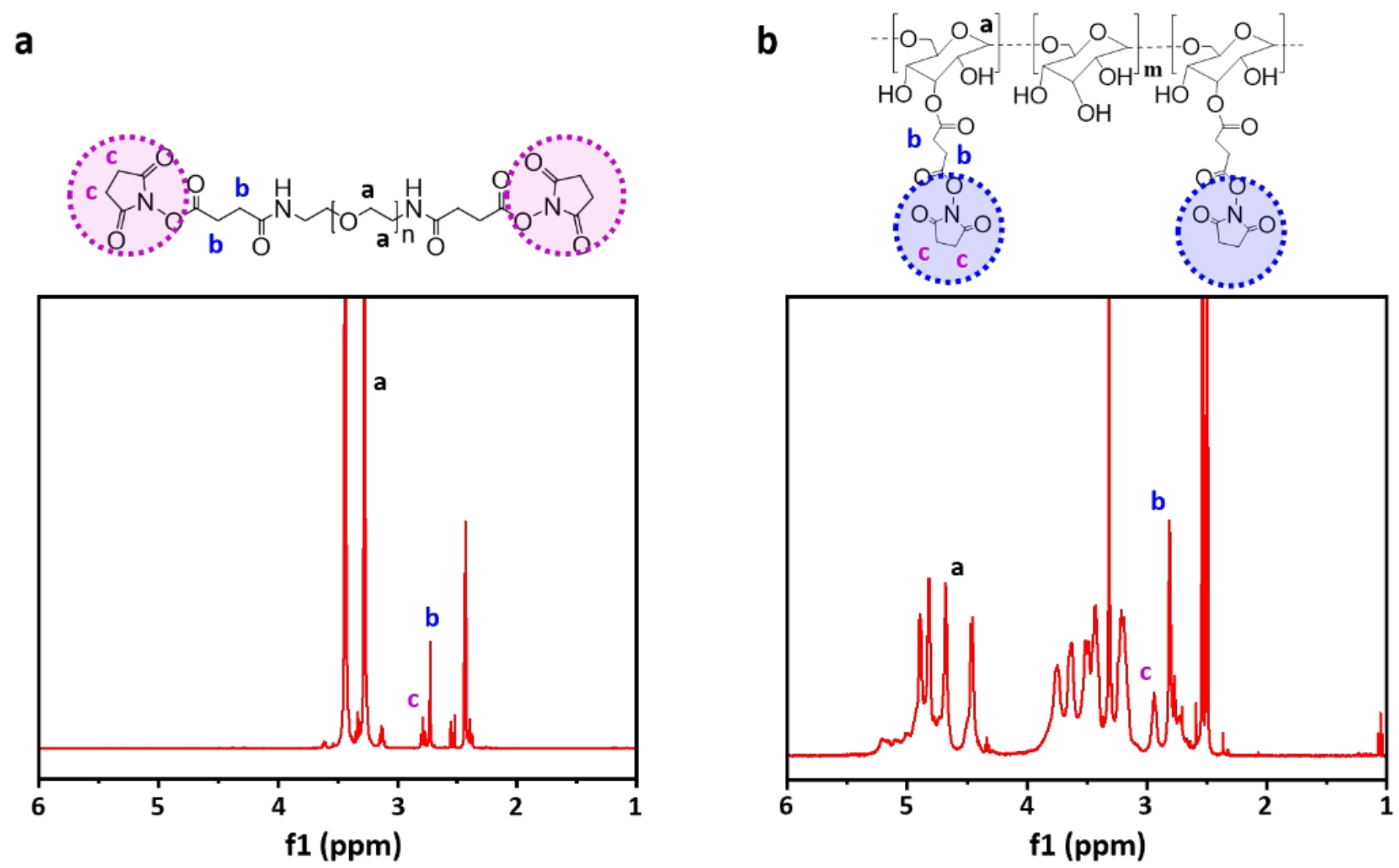

Supplementary Figure 1. ^{1}H NMR spectra of a purchased NHS-activated PEG (Mw = 2 kD, Sigma-Aldrich) (**a**) and synthesized NHS-activated dextran (Mw = 40 kD) (**b**). Polymers were dissolved in DMSO-d_6. The monomer ratio of NHS in dextran was *ca.* 10 %.

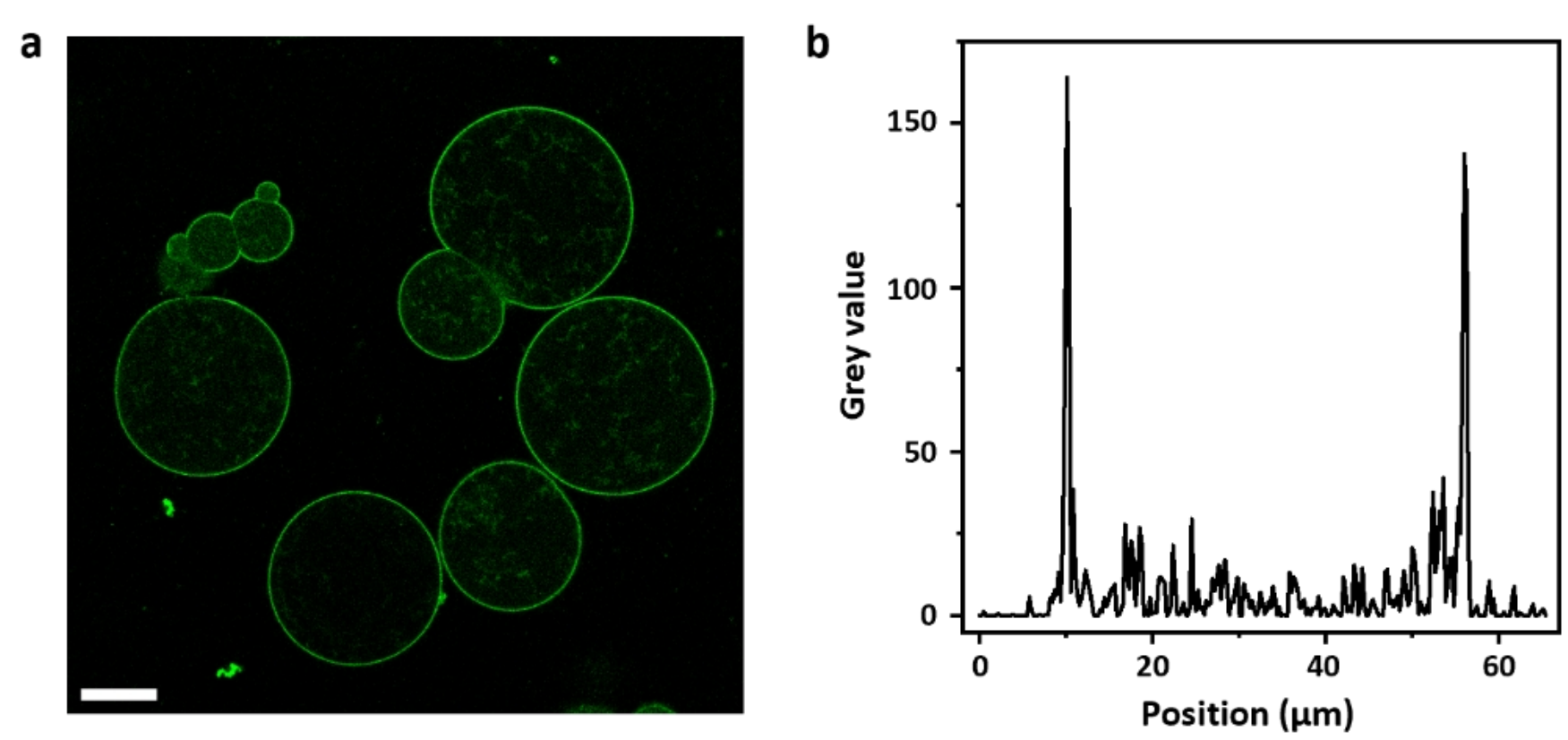

Supplementary Figure 2. (**a**) CLSM image of FITC-labelled Psome-PEG protocells prepared without encapsulated enzymes. A continuous membrane-like structure is evident on the protocell surface. (**b**) Line profile across an individual proteinosome showing the distribution of FITC-labelled nanoconjugates within the protocell. Scale bar, 20 μm. The protocells were formed using a low concentration (ca. 3 mg/ml) of FITC-labelled BSA/PNIPAAm nanoconjugates. The amphiphilic nanoconjugates are used to construct the proteinosome membrane by spontaneous interfacial self-assembly within a water-in-oil Pickering emulsion. The resulting water droplet/oil interface is stabilized using crosslinking agents, and the resulting microstructures transferred into an aqueous phase to give semi-permeable proteinosomes. See Ref 38 in the main text for further details.

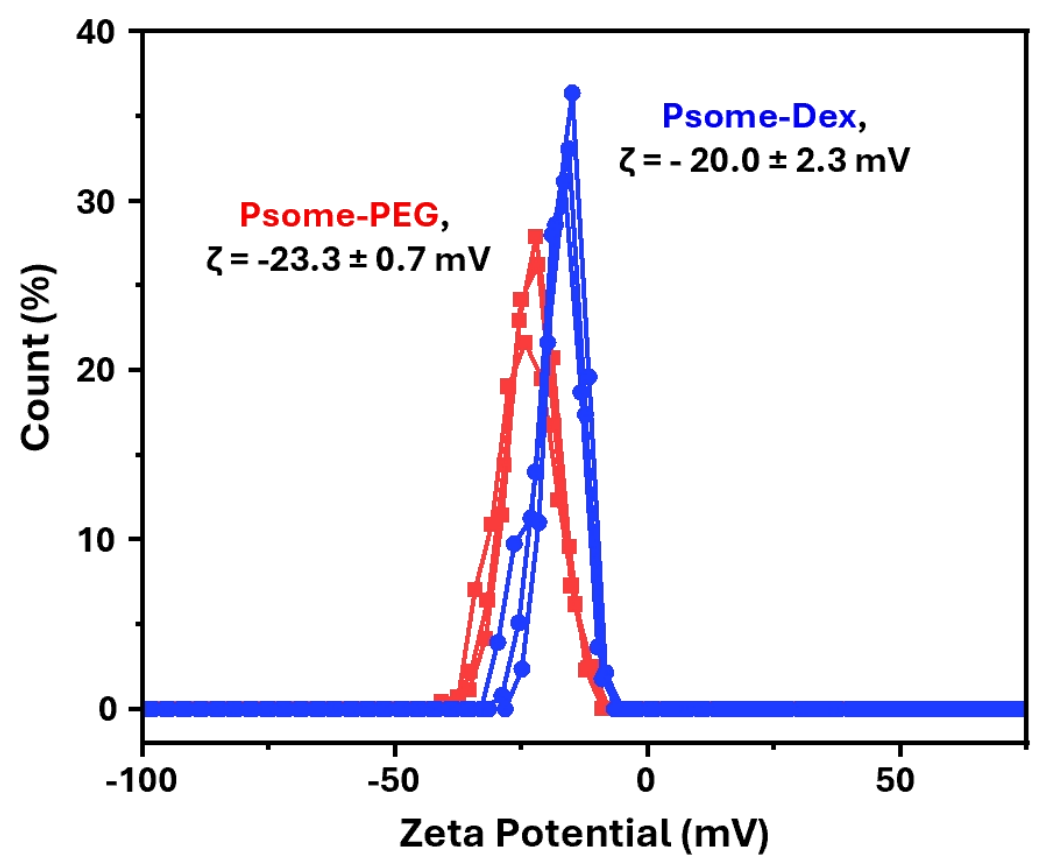

Supplementary Figure 3. Zeta potential of aqueous suspensions of Psome-Dex and Psome-PEG. pH values were *ca*. 7.0 in both cases.

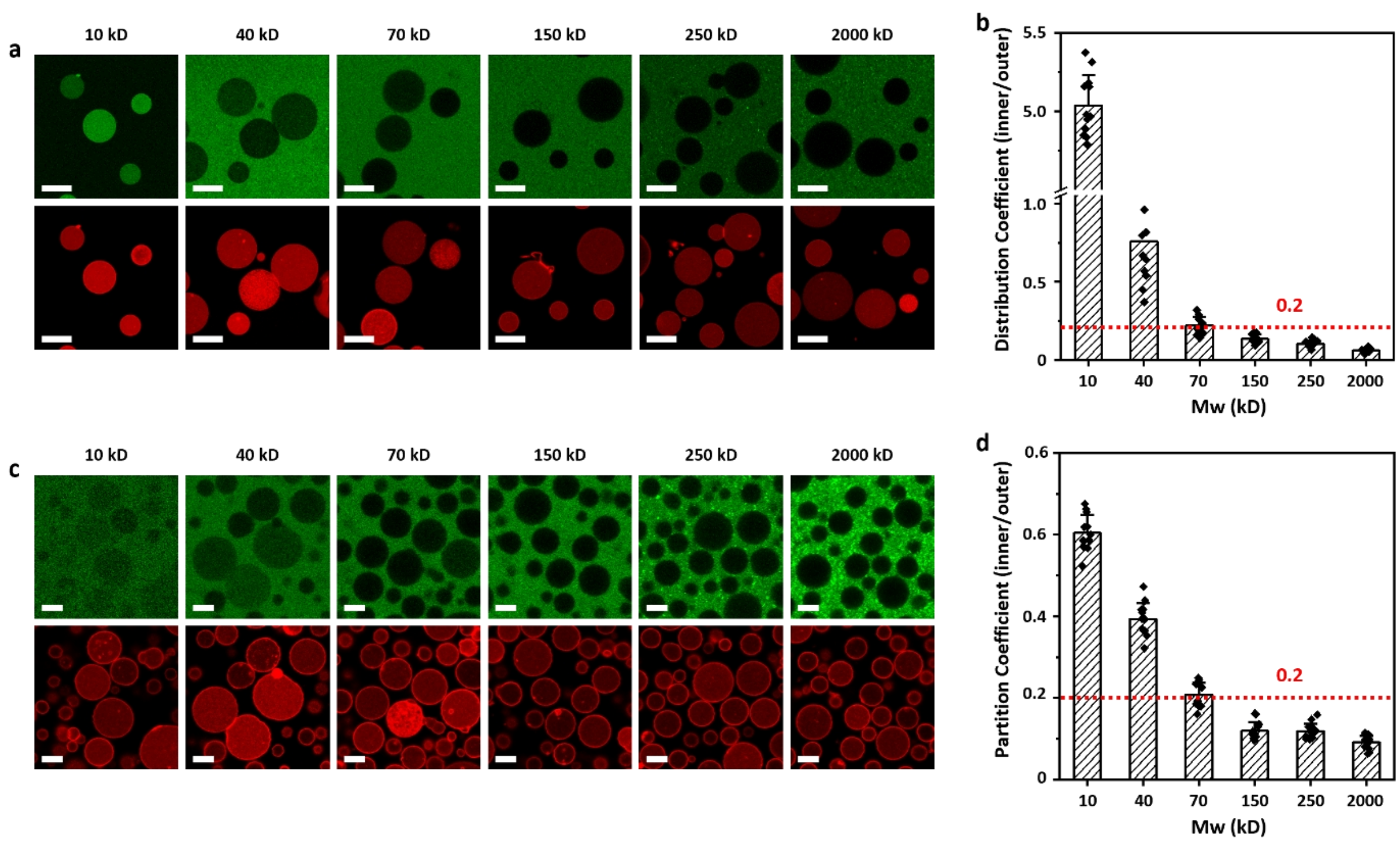

Supplementary Figure 4. (**a**,**b**) CLSM images (**a**) of dispersions of RITC-labelled Psome-PEG red fluorescence) incubated with FITC-dextran (green fluorescence) of different molecular weights (Mw: 10-2000 kDa) and corresponding bar charts (**b**) showing the effective partition coefficients of FITC-dextran. (**c,d**) CLSM images (**c**) of dispersions of RITC-labelled Psome-Dex incubated with FITC–dextran of different molecular weights (Mw: 10-2000 kDa) and corresponding bar charts of the partition coefficients (**d**). CLSM images were acquired after 24 h incubation. Partition coefficients were determined by determining the ratio of green fluorescence intensity (grey value) inside : outside the proteinosomes using ImageJ software. Error bars: n (protocells) = 10 from ≥ 3 CLSM images obtained at different dextran molecular weights, mean values ± SD in (**b,d**).

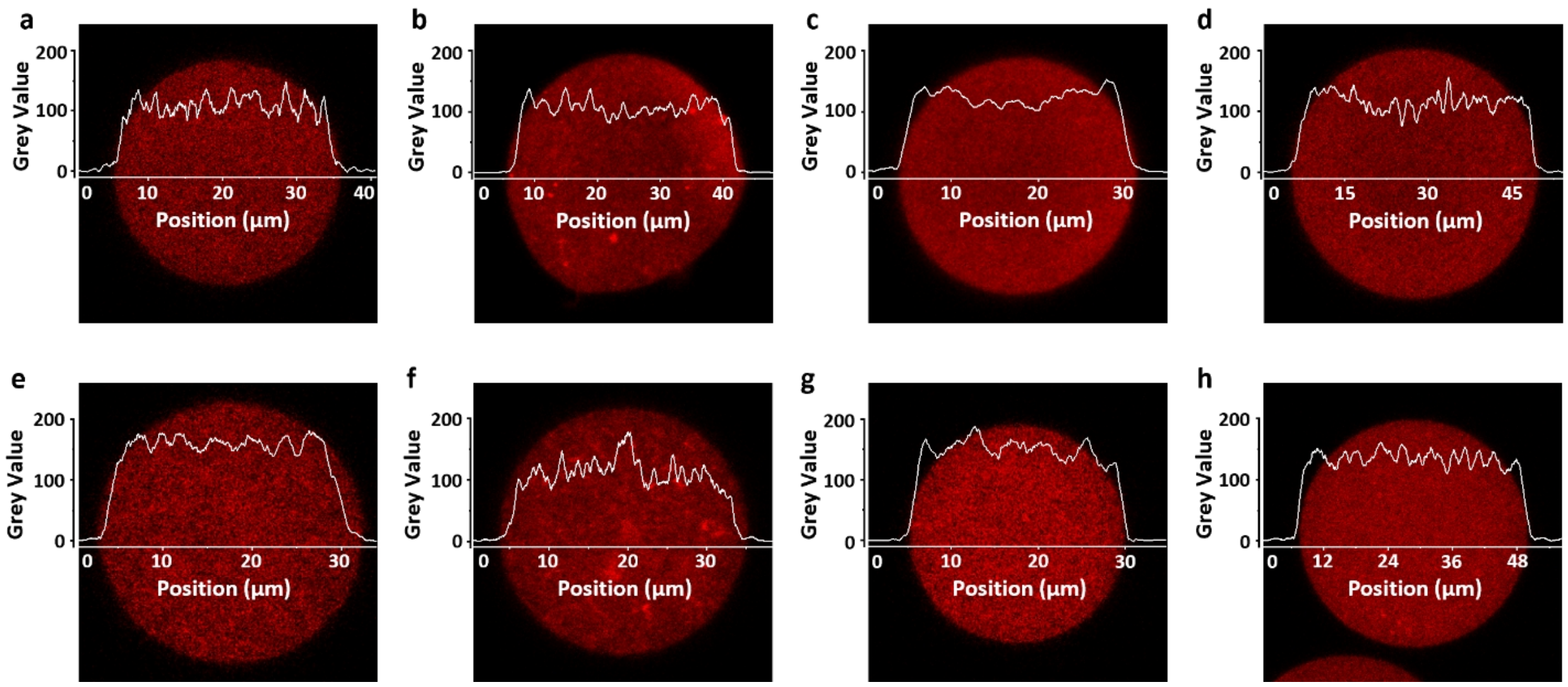

Supplementary Figure 5. (**a**-**d**) CLSM images of Psome-Dex containing RITC-labelled amylase (**a**), GOx (**b**), HRP (**c**), and acid phosphatase (AP) (**d**). (**e**-**h**) CLSM images of Psome-PEG containing RITC-labelled amylase (**e**), GOx (**f**), HRP (**g**), and AP (**h**). Inserts are line profiles showing the distribution of the RITC-labelled payloads inside the proteinosomes. Molecular weights; amylase (Mw, *ca.* 50 kD), GOx (Mw, *ca.* 160 kD), HRP (Mw, *ca.* 40 kD), ALP (Mw, *ca.* 140 kD).

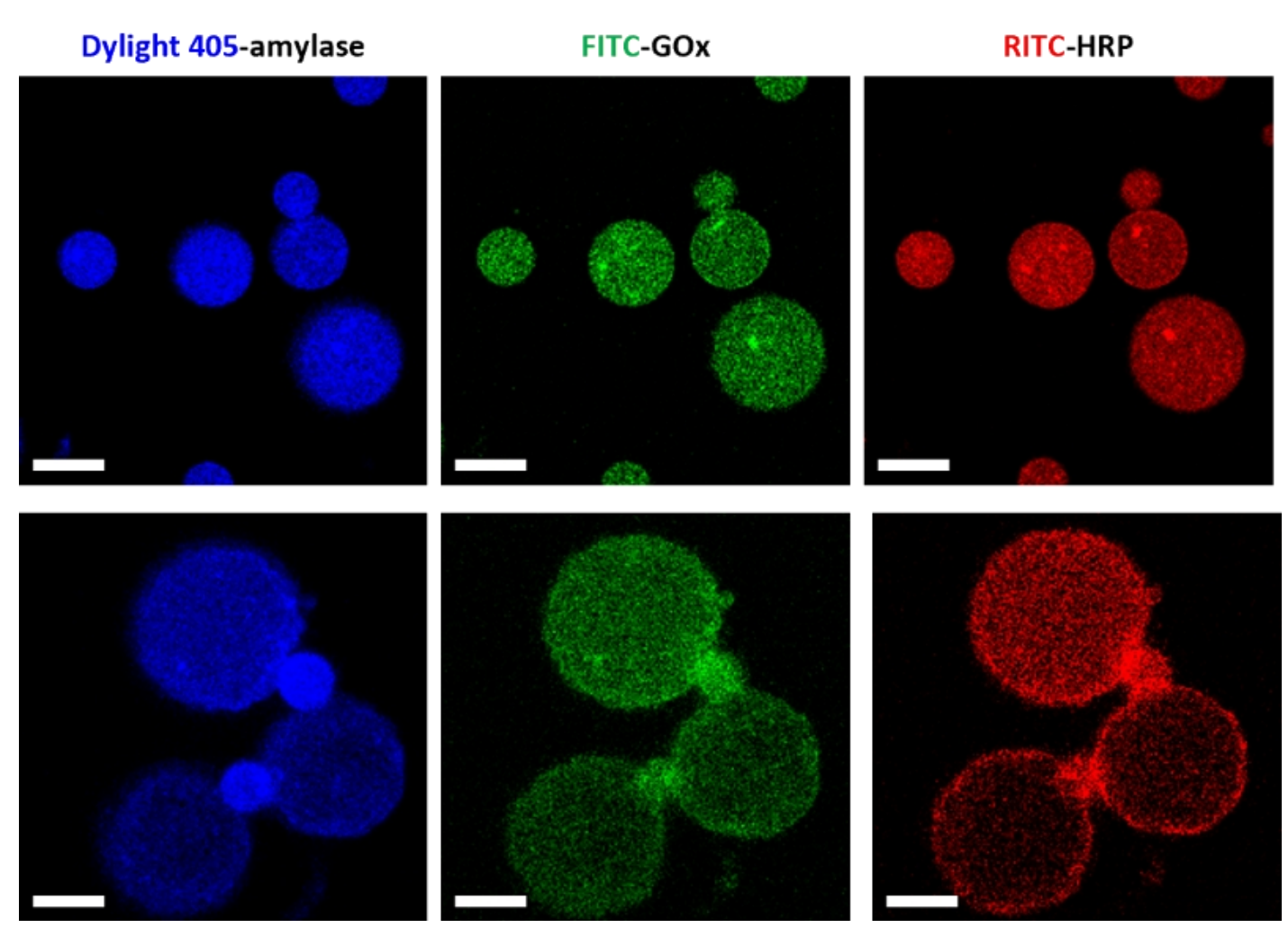

Supplementary Figure 6 CLSM images of Psome-PEG (upper row) and Psome-Dex (bottom row) protocells co-encapsulated with Dylight 405-labelled α-amylase (left), FITC-GOx (middle) and RITC-HRP (right).

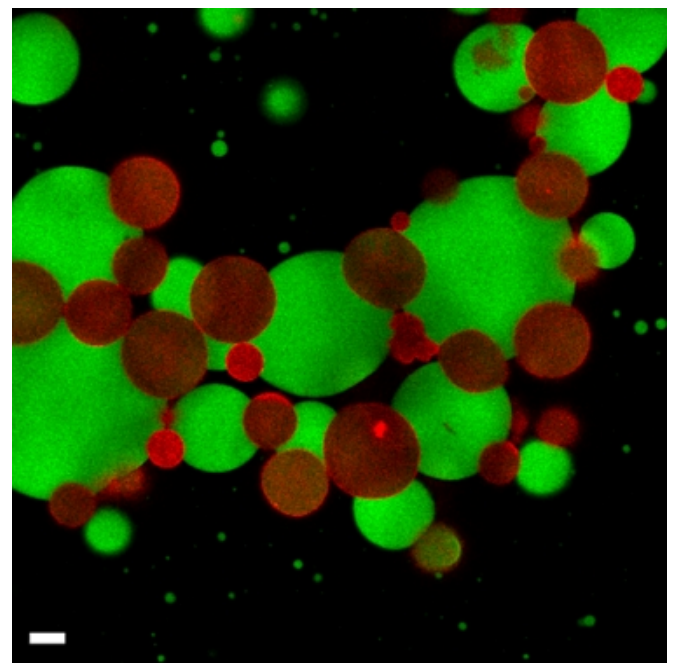

Supplementary Figure 7. CLSM image of a dextran-in-PEG ATPS containing RITC-labelled proteinosomes (red) cross-linked by both PEG-NHS and Dex-NHS (ratio = 1:1, w/w). ATPS composition: dextran (35-45 kD), 35 mg/ml; PEG (35 kD), 104 mg/ml. Dextran is labelled with FITC (green). Proteinosome density: 1.0 x 10^7/mL. Scale bar, 20 µm.

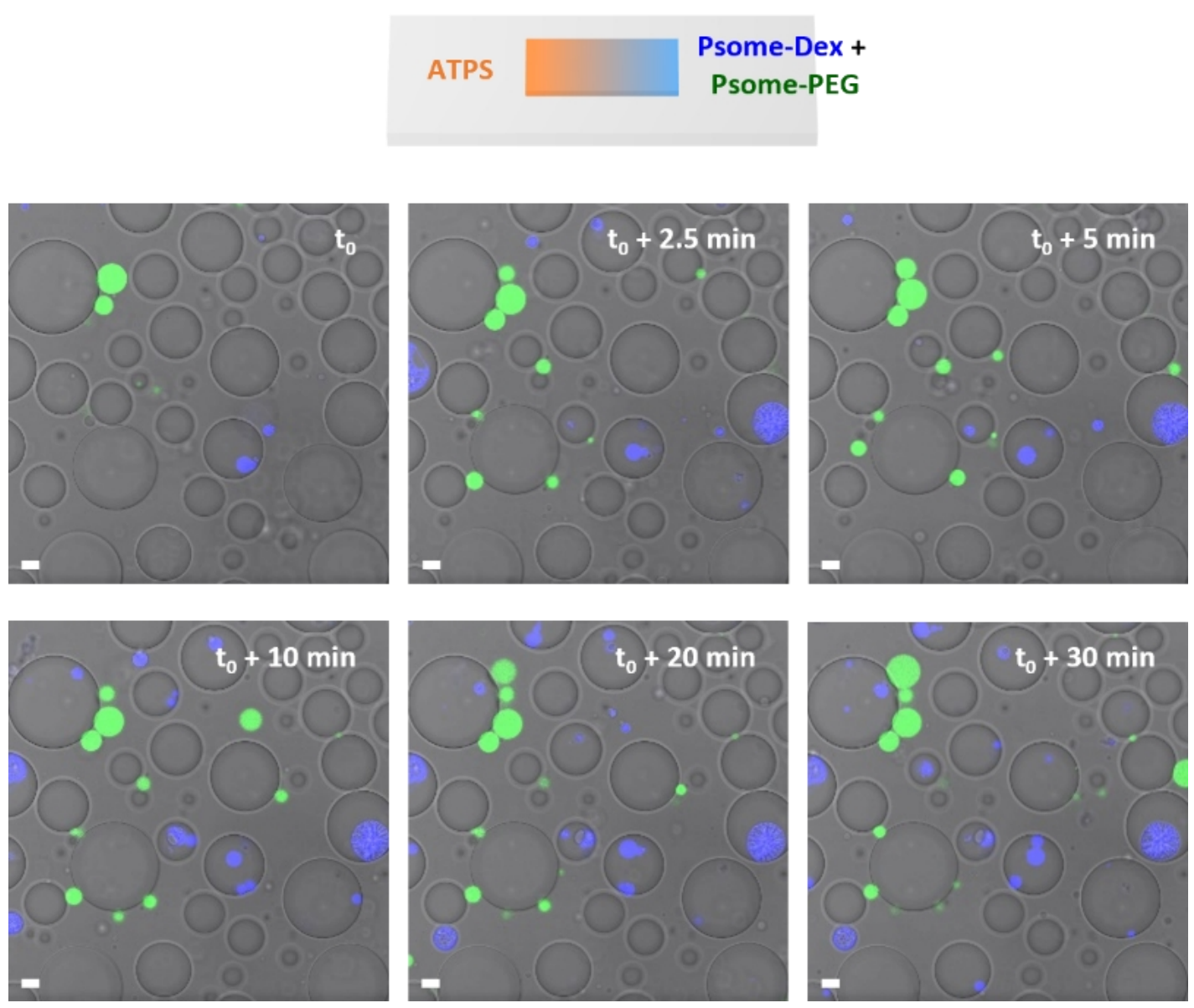

Supplementary Figure 8. Time-sequence of CLSM images showing the spontaneous segregation of mixed populations of Psome-Dex (blue) and Psome-PEG (green) protocells in ATPS. The mixed protocells are added to the ATPS housed within a glass channel and diffuse into the ATPS. Protocell density: Psome-Dex, 0.6 x 107; Psome-PEG, 1.0 x 107/ml, respectively. Scale bars. 20 µm.

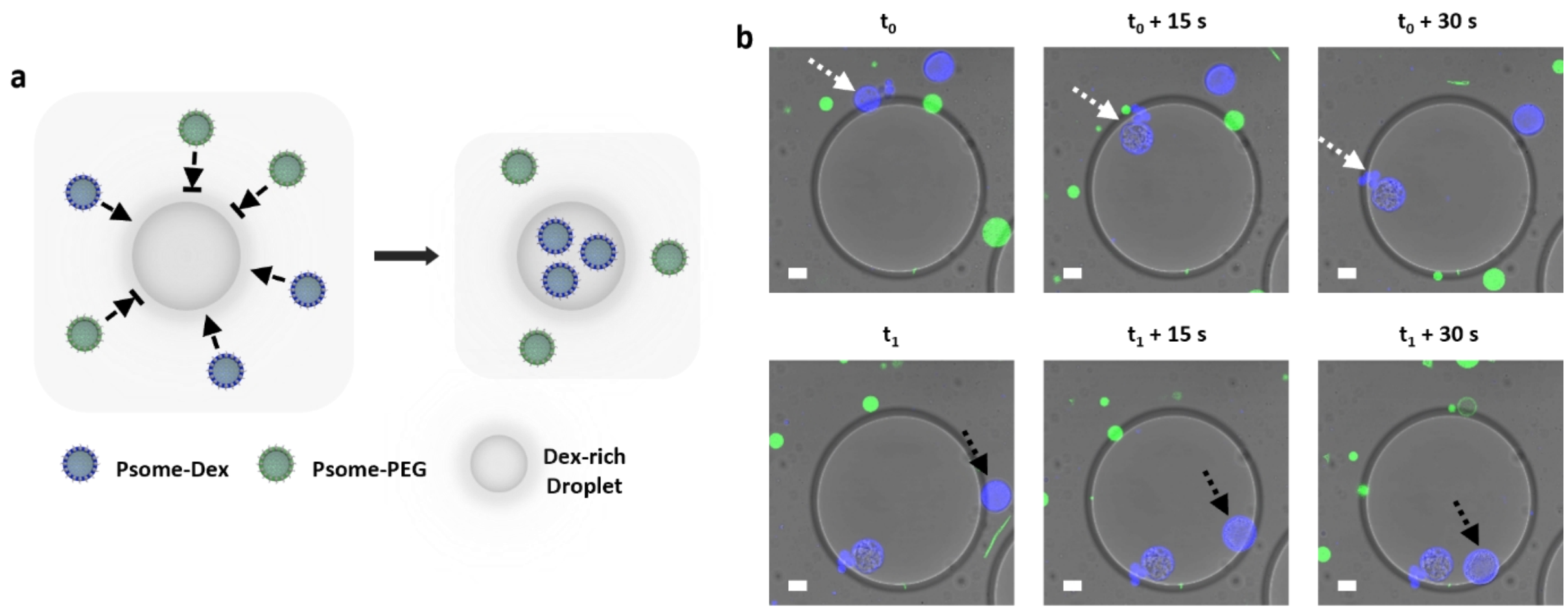

Supplementary Figure 9. (**a**) Diagram showing the different motions of Psome-Dex (blue) and Psome-PEG (green) after transferring into an ATPS to generate self-sorting. (**b**) Time-sequence of CLSM images showing protocell translocation and segregation in the ATPS. Upper and lower images record the unidirectional translocation of two dextran-functionalized Psomes from the continuous PEG phase into the dextran droplets, as well the confined motion of Psome-PEG in the continuous PEG-rich phase. Scale bar, 20 µm.

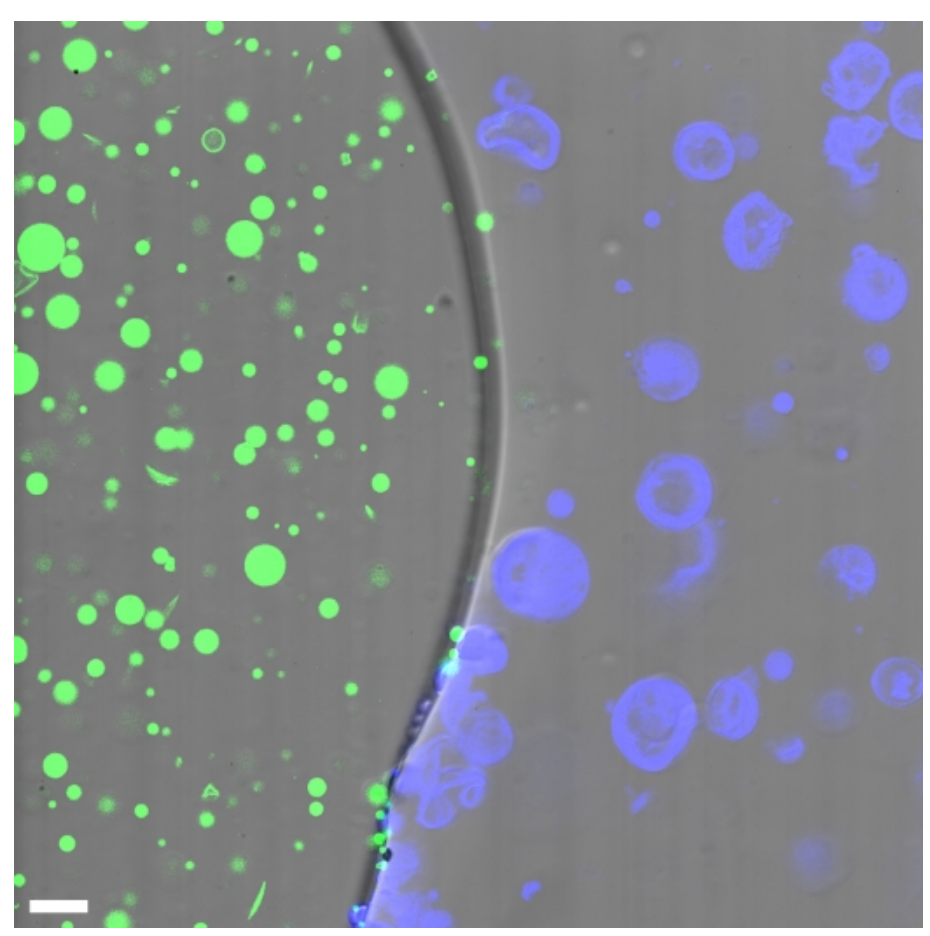

Supplementary Figure 10. CLSM image showing bulk segregation of a binary proteinosome population in an unstirred dextran-in-PEG ATPS. Image was recorded after 24 h. Protocell number densities; 0.6 x 10^7/ml (Psomes-Dex); 1.0 x 10^7/ml (Psomes-PEG). Scale bar, 50 µm.

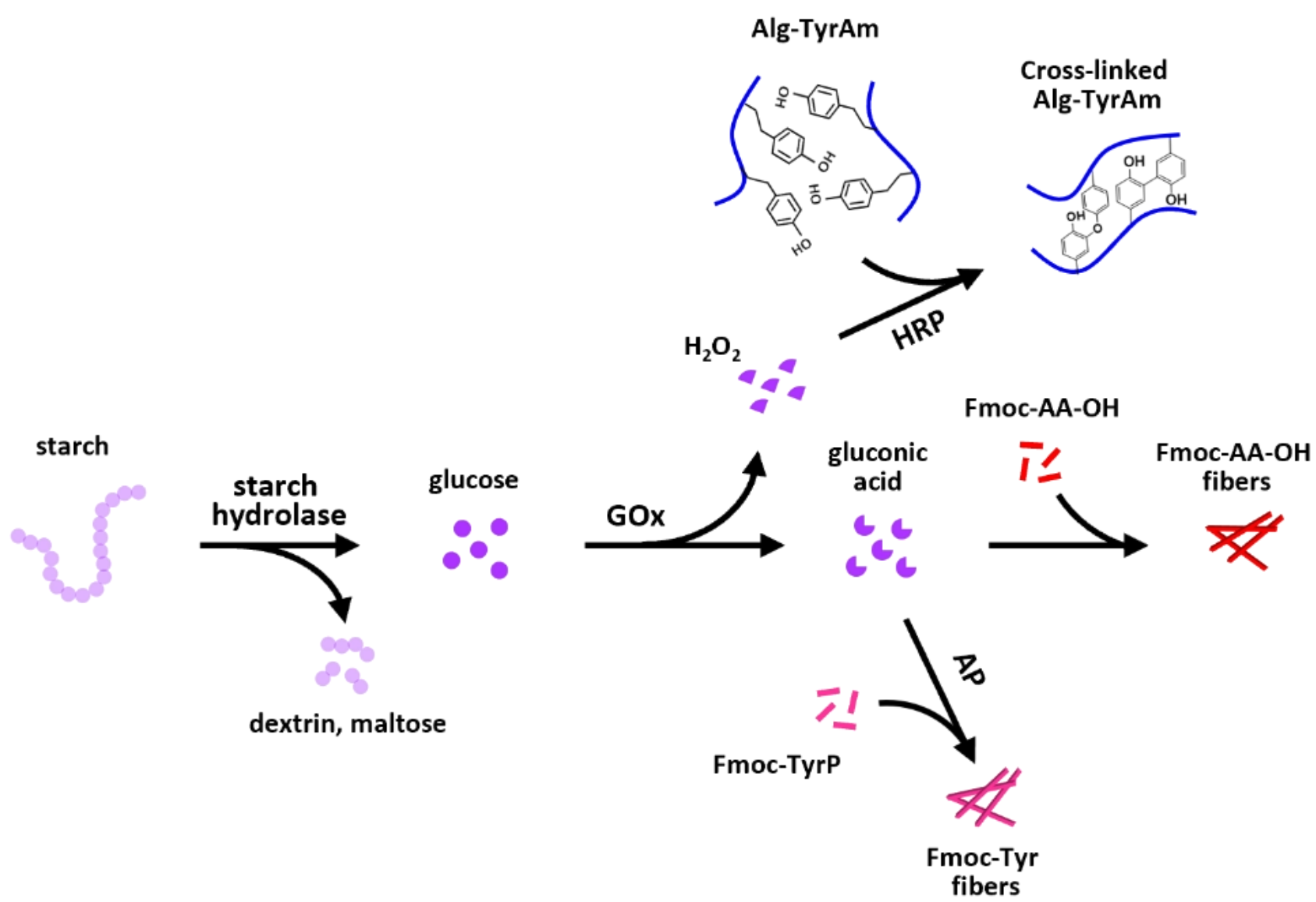

Supplementary Figure 11. Role of proteinosome-encapsulated enzyme cascades in the implementation of in situ changes in protocell membrane wettability (phase compatibility). A three-step enzyme cascade consisting of starch hydrolase (SH, α-amylase and amyloglucosidase (w/w = 1)), glucose oxidase (GOx) and horseradish peroxidase (HRP) is used to initiate the coating of the proteinosomes with a dextran-compatible crosslinked polysaccharide shell (tyramine-derivatized alginate, Alg-TyrAm), or a shell of PEG-compatible dipeptide (Fmoc-AA-OH) or amino acid (Fmoc-TyrP) fibres. In all cases, SH catalyses the hydrolysis of a starch input, thereby generating glucose (along with by-products such as dextrin and maltose), which initiates a signalling cascade within individual proteinosomes (single populations) or between protocells (binary populations). The released glucose is subsequently oxidized by GOx to yield hydrogen peroxide (H_2O_2) and gluconolactone (GL). H_2O_2 activates HRP, leading to the oxidative crosslinking of Alg-TyrAm. GL slowly hydrolyses to D-gluconic acid, which results in acidification of the medium that induces peptide fibre self-assembly or activates acid phosphatase (AP)-mediated formation of amino acid fibres by dephosphorylation of Fmoc-TyrP.

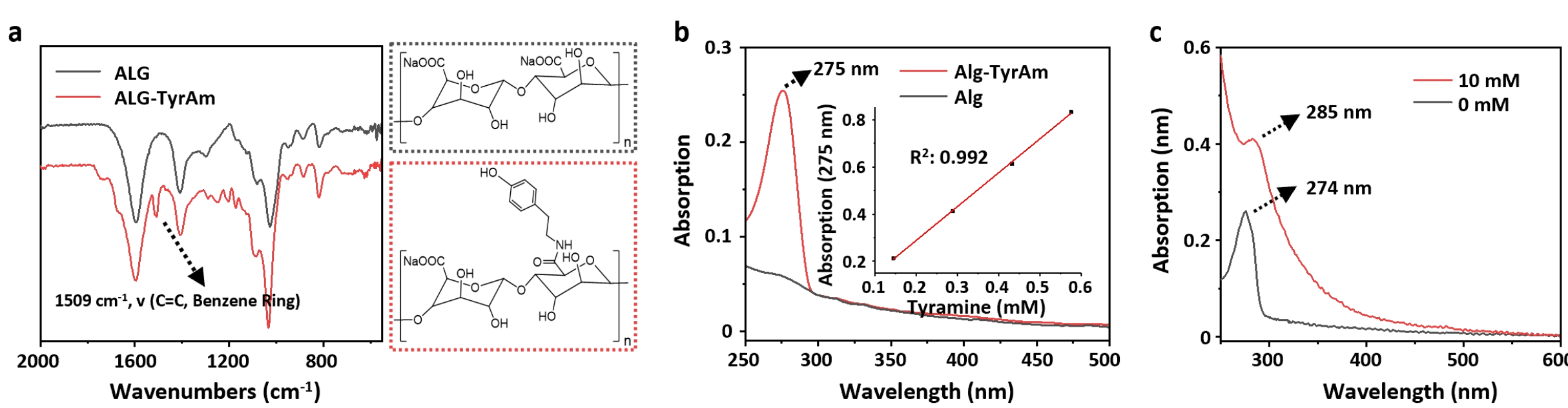

Supplementary Figure 12. Characterization of tyramine-functionalized alginate (Alg-TyrAm). (**a**) FTIR spectra of alginate (black) and Alg-TyrAm (red) and corresponding molecular structures. (**b**) Absorption profiles of alginate (black) and Alg-TyrAm (red); the absorption peak at 275 nm confirms the presence of the tyramine group. Inset is a calibration curve used to evaluate TyrAm content in Alg-TyrAm (*ca*. 7 % (n/n)). Polysaccharide concentration, *ca*. 1 mg/ml. (**c**) Absorption profiles of aqueous Alg-TyrAm solutions before (0 mM glucose) and after GOx/HRP-induced

covalent cross-linking (10 mM glucose). GOx/HRP concentration, *ca*. 50/50 IU/ml. Alg-TyrAm concentration, ca. 1 mg/ml. Profiles were recorded 12 h after glucose addition.

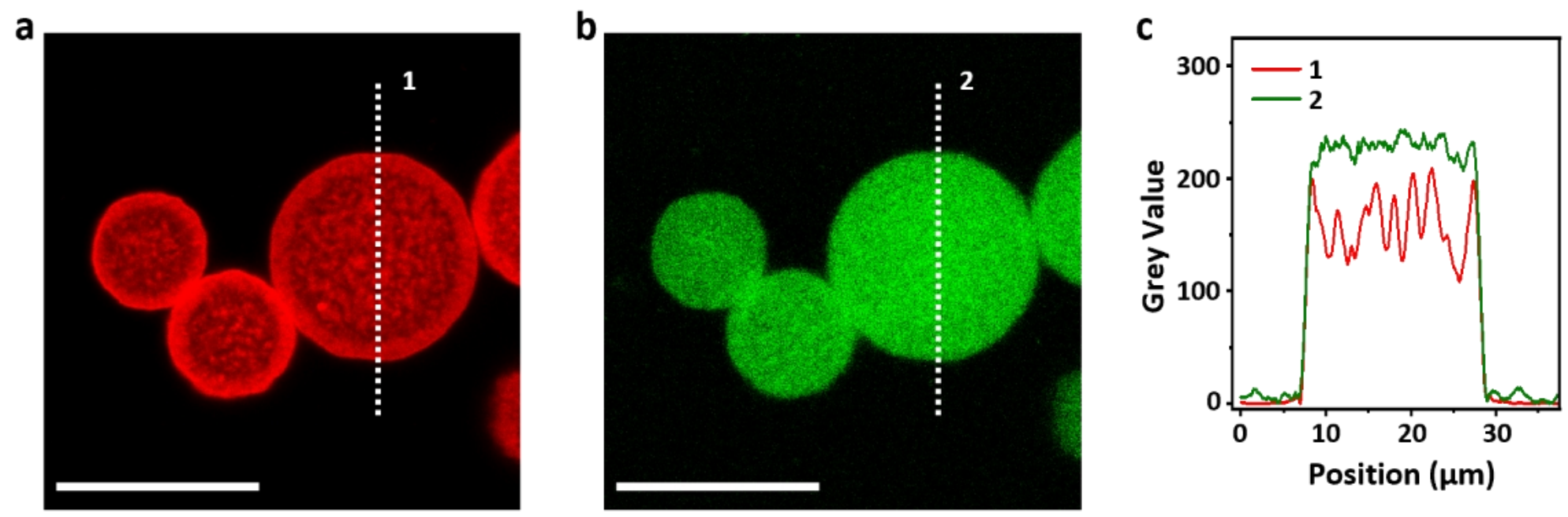

Supplementary Figure 13. CLSM filtered images of Alg-TyrAm-modified SH/GOx/HRP-containing Psome-PEG constructs showing native RITC-labelled BSA/PNIPAAm nanoconjugates in the proteinosome membrane and interior (red) (**a**) and presence of FITC-labelled Alg-TyrAm (Mw: 140-160 kD) (**b**). (**c**) Corresponding line profiles. The CLSM image was recorded after removal of dissolved components in the background. The presence of Alg-TyrAm on both the surface and interior of the protocells indicates penetration of the polysaccharide through the protocell membrane. Alg-TyrAm concentration, 2 mg/ml; Psome-PEG number density, 1.8 x 10^7/ml. Scale bars, 20 μm.

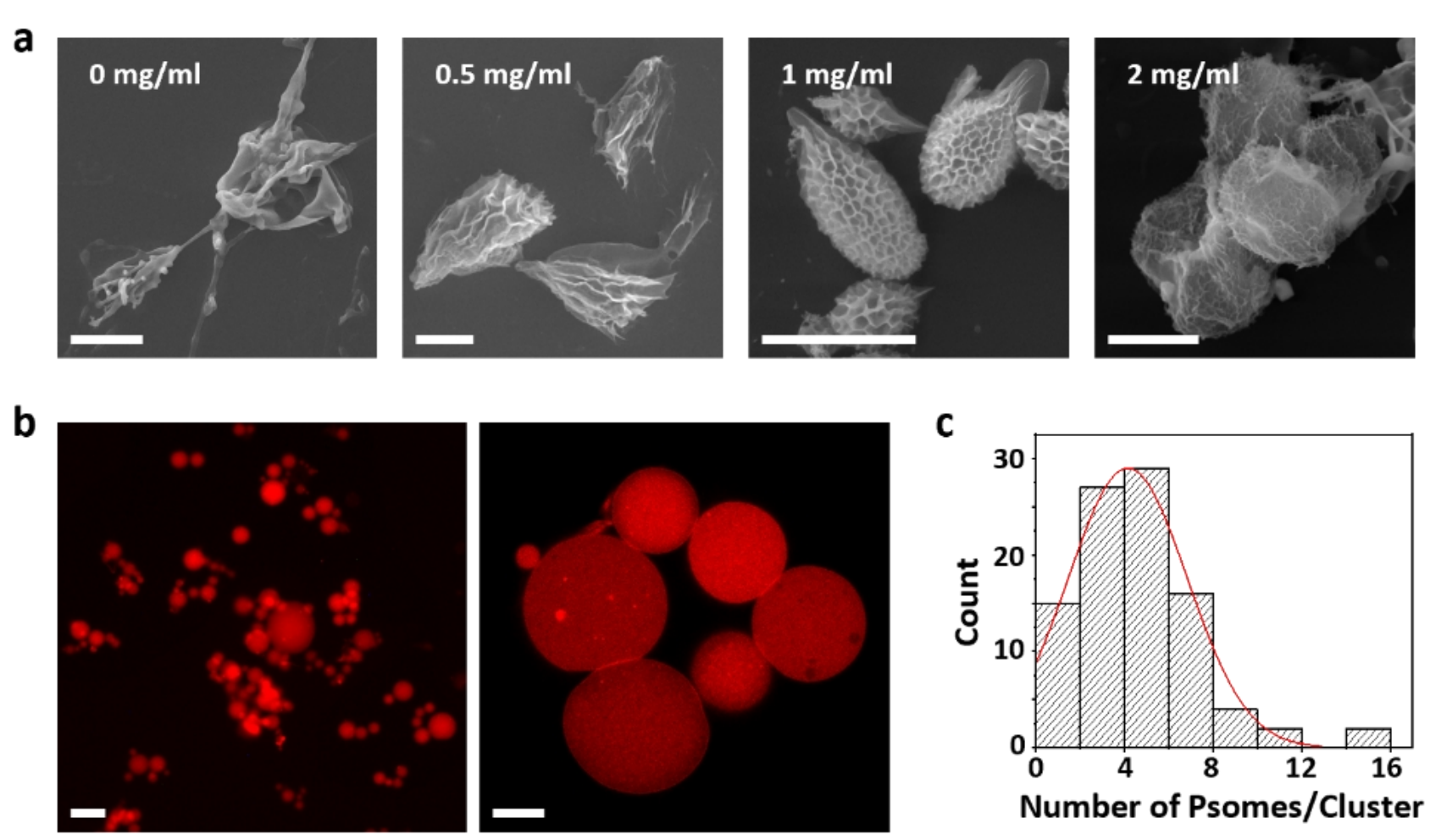

Supplementary Figure 14. (**a**) SEM images of SH/GOx/HRP-containing Psome-PEG protocells before (0 mg/ml) and after modification with Alg-TyrAm (Mw: 140-160 kD; (0.5, 1 and 2 mg/ml)). (**b**) Fluorescence microscopy (left) and CLSM (right) images of clusters of Alg-TyrAm-modified SH/GOx/HRP-loaded Psome-PEG produced at a Alg-TyrAm concentration of 2 mg/ml. Glucose concentration, 10 mM. (**c**) Number distribution of proteinosomes (Psomes) in the clusters. n = 95, mean value = 4.2. Scale bar, 20 μm.

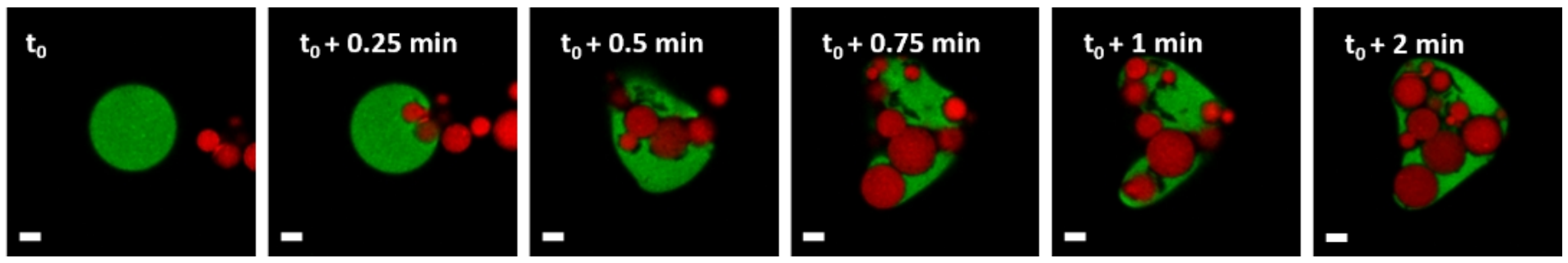

Supplementary Figure 15. Time-series of CLSM images showing progressive translocation of a cluster of Alg-TyrAm-modified SH/GOx/HRP/Psome-PEG protocells (red fluorescence) from a PEG continuous phase into a single dextran droplet (green fluorescence) of an ATPS. Modification of the proteinosome membrane was undertaken in an aqueous solution of Alg-TyrAm and then transferred into the PEG continuous phase. Fluorescent labels; red (RITC-BSA/PNIPAAm membrane nanoconjugate); green (FITC-labelled dextran). Alg-TyrAm, 2 mg/ml; glucose, 10 mM; scale bar, 20 µm.

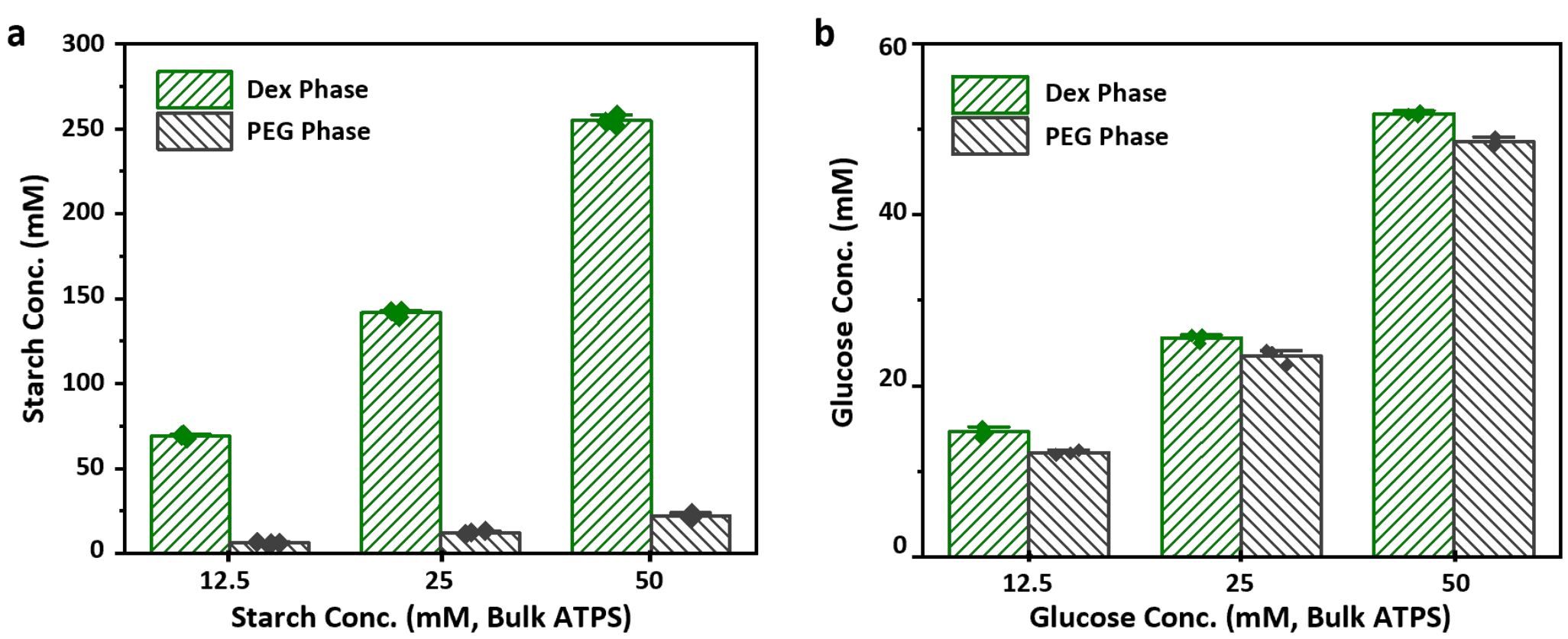

Supplementary Figure 16. Bar charts showing the distribution of dissolved Azure starch (**a**) and glucose (**b**) in a Dex-in-PEG ATPS. Corresponding Dex : PEG partition constants are approximately 11.5 (starch) and 1.1 (glucose). Error bars: n = 3 (repeated groups of samples), mean values ± SD in (**a,b**).

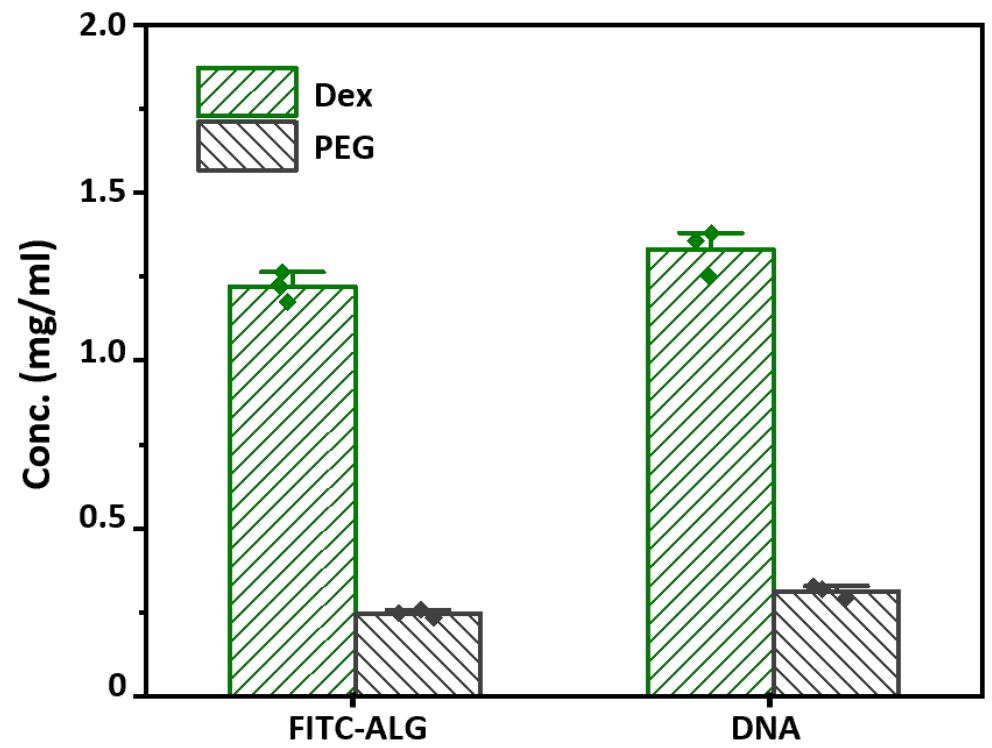

Supplementary Figure 17. Bar charts showing the distribution of alginate and dsDNA in a Dex-in-PEG ATPS. Corresponding Dex : PEG partition constants are approximately 5.0 (alginate) and 4.3 (dsDNA). Bulk concentrations of alginate and DNA in the ATPS are ca. 0.5 mg/ml. Error bars: n = 3 (repeated groups of samples), mean values ± SD in (**a**,**b**).

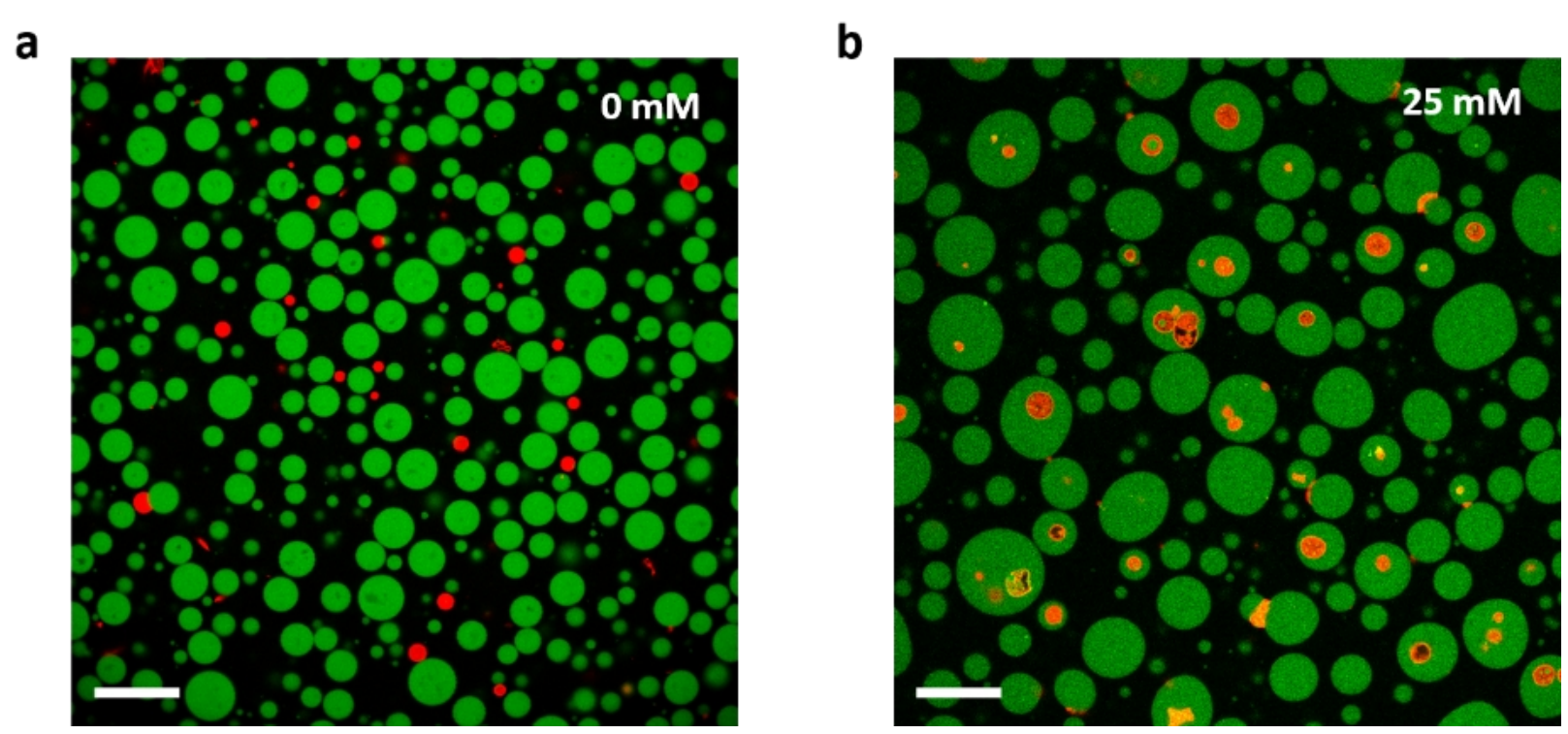

Supplementary Figure 18. CLSM images recorded in the absence (**a**) or 24 h after the addition of (**b**) starch to an ATPS containing SH/GOx/HRP/Psome-PEG protocells (red fluorescence) showing translocation of the proteinosomes from the PEG continuous phase into the dextran droplets (green fluorescence). Modification of the proteinosome membrane was undertaken *in situ* within the PEG continuous phase. Alg-TyrAm, 1 mg/ml; starch, 0 or 25 mM; scale bar, 50 µm.

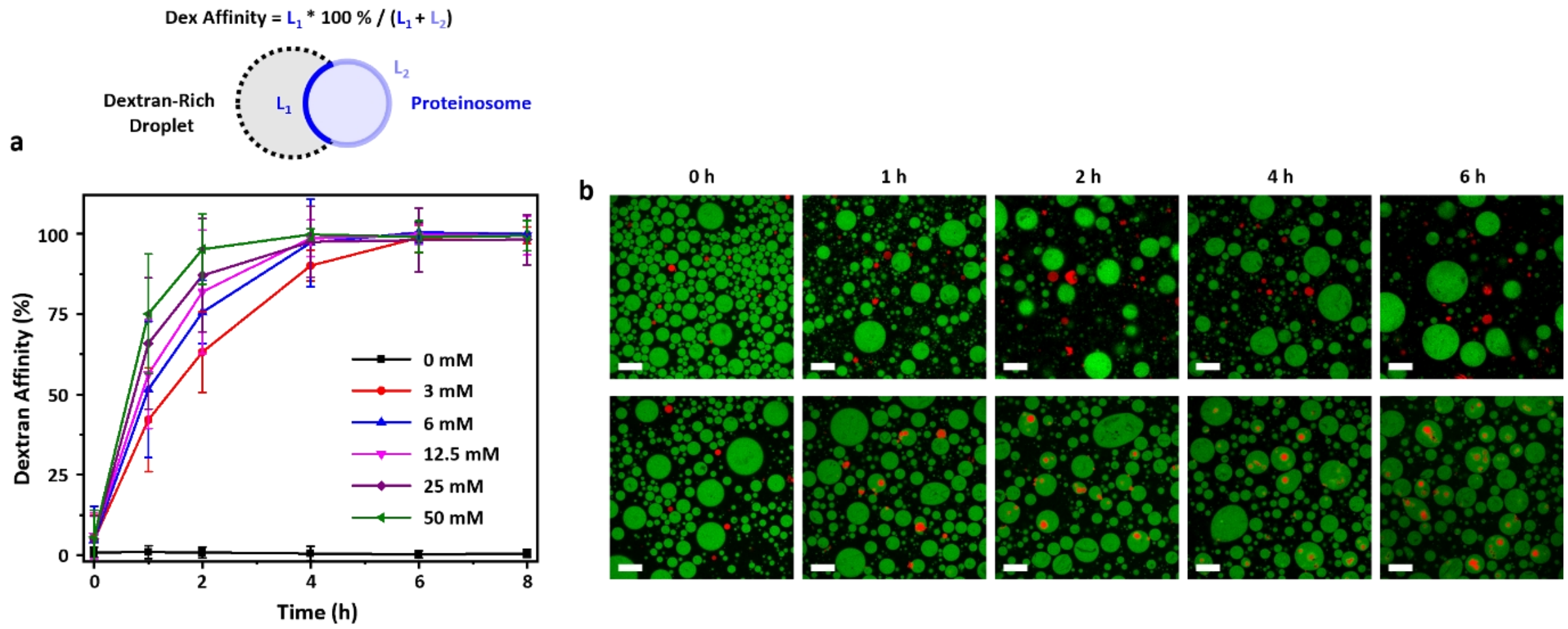

Supplementary Figure 19.(**a**) Time-dependent plots of number of Alg-TyrAm-modified GOx/HRP/Psome-PEG protocells present in the dextran-enriched droplets (dextran affinity) of an ATPS after addition of glucose (t = 0). Modification of the protocells was undertaken in the PEG continuous phase at different glucose concentrations and numbers of transferred proteinosomes obtained from CLSM images. No phase transfer is observed in the absence of membrane modification (0 mM glucose). Error bars: n = 3 (repeated groups of samples), mean values ± SD. (**b**) CLSM images showing the time-dependent transfer of Psome-PEG protocells (red) in the ATPS after glucose addition. Glucose concentration: 0 (upper) and 12.5 mM (bottom). Alg-TyrAm, 1 mg/ml. Dextran was FITC-labelled. Psome-PEG was RITC-labelled. Scale bars, 50 µm.

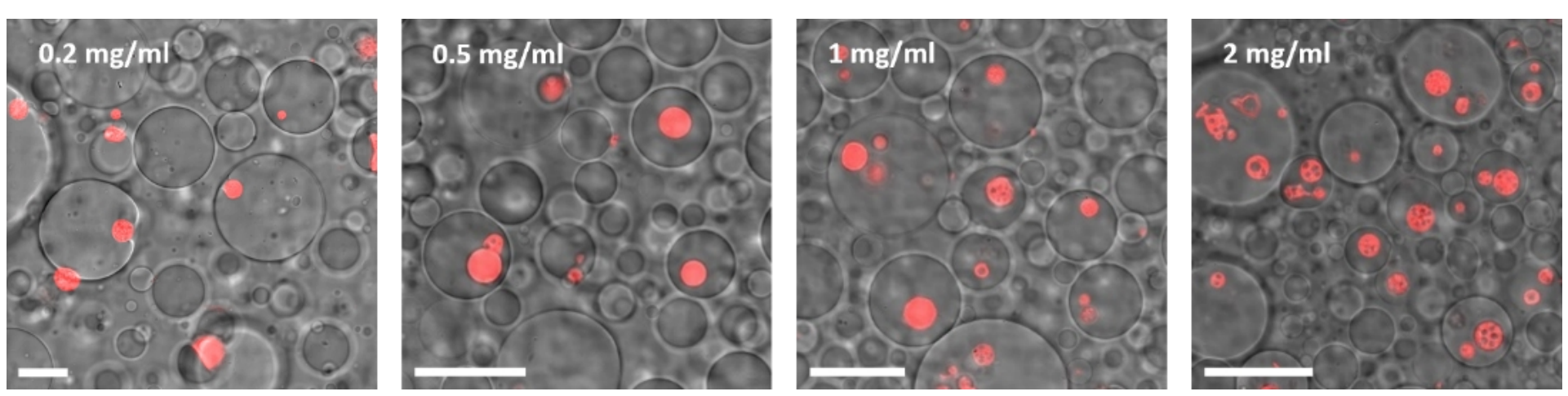

Supplementary Figure 20. CLSM images of ATPS suspensions containing RITC-labelled GOx/HRP-loaded Psome-PEG protocells after in-situ crosslinking of Alg-TyrAM at different concentrations (0.2, 0.5, 1, 2 mg/ml). At 0.2 mg/ml Alg-TyrAM, the dextran affinity is ca. 64.0 ± 30.1%, whereas at 0.5-2 mg/ml, the dextran affinities are all > 95%. Glucose, ca. 10 mM, in all cases. Reaction time, ca. 5 h. Scale bar 50 µm in length.

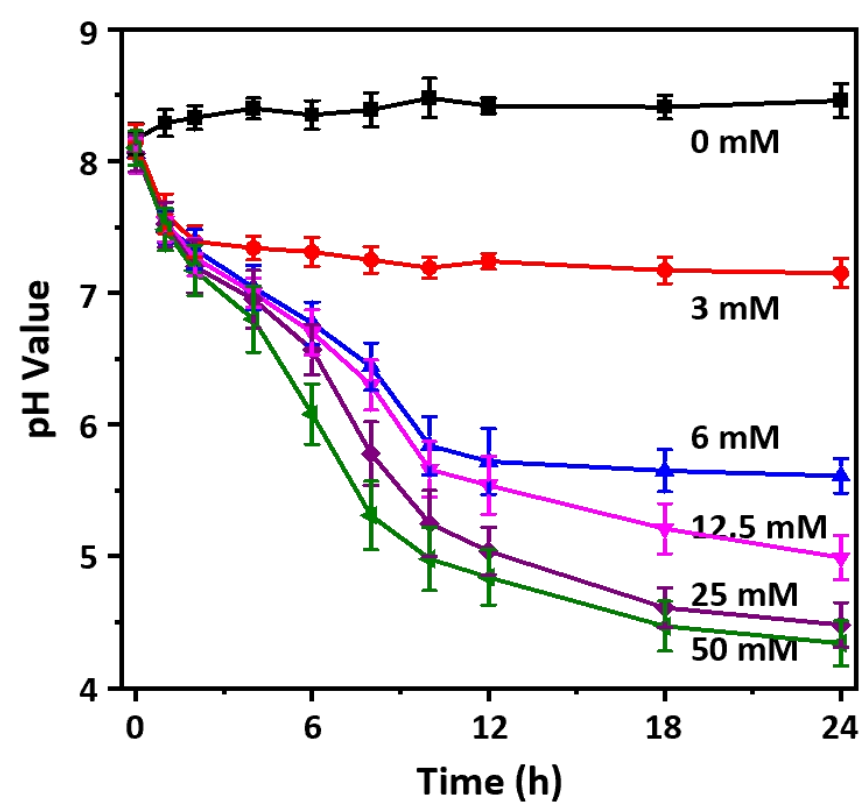

Supplementary Figure 21 Time-dependent plots showing changes in pH values of a dextran-in-PEG ATPS containing GOx/HRP-loaded Psome-PEG protocells, dissolved Alg-TyrAm and glucose at various concentrations (0–50 mM). Psome-PEG density: 1.8 × 10^7/ml. Error bars: n = 3 (repeated groups of samples), mean values ± SD.

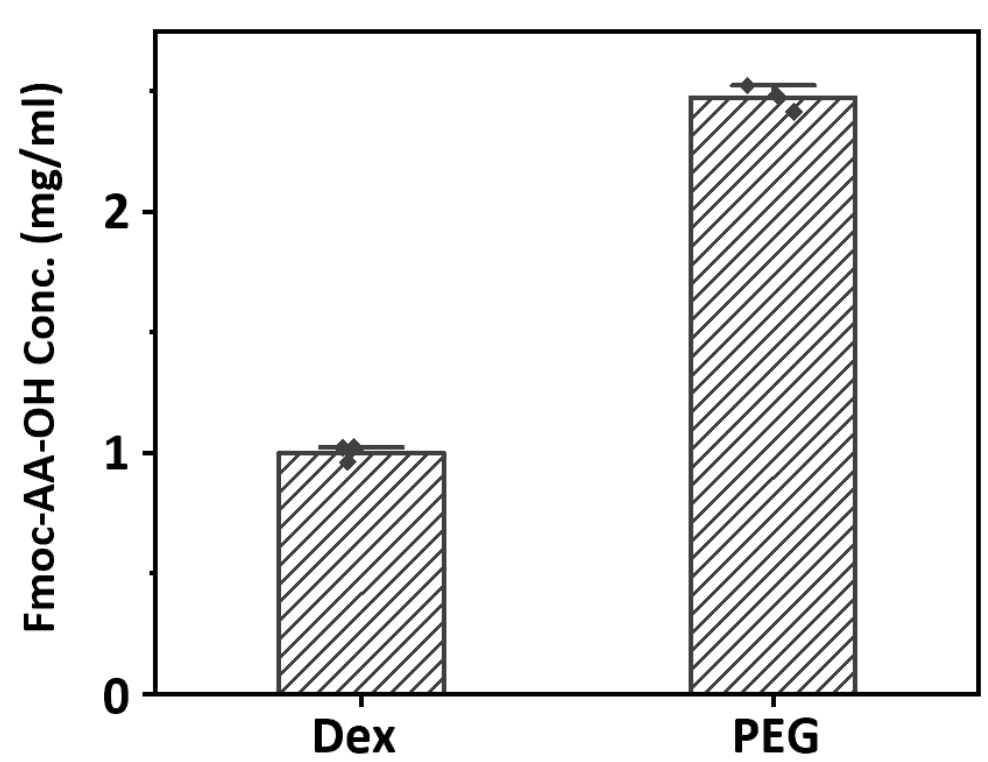

Supplementary Figure 22. Bar chart showing distribution of dissolved Fmoc-AA-OH monomers (pH 9) in a Dex-in-PEG ATPS. Corresponding Dex : PEG partition constant is approximately 0.4. Bulk concentration of Fmoc-AA-OH monomers is *ca.* 2.3 mg/ml. Error bars: n = 3 (repeated groups of samples), mean values ± SD.

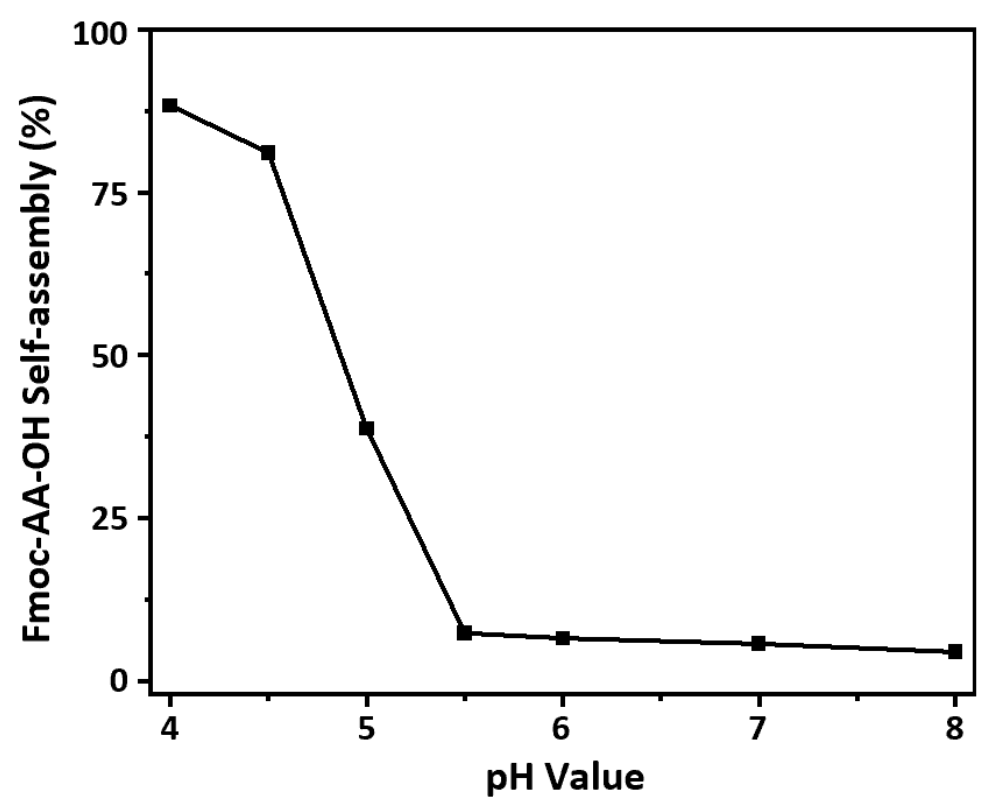

Supplementary Figure 23. Graph showing the pH-responsive self-assembly of Fmoc-AA-OH (*ca*. 3 mg/ml) in water. The percentage of self-assembled peptide fibrils was determined by collecting the supernatant from Fmoc-AA-OH solutions at different pH values after centrifugation (10000 rpm, 5min) and recording the absorption at 299 nm using a UV/VIS spectrometer.

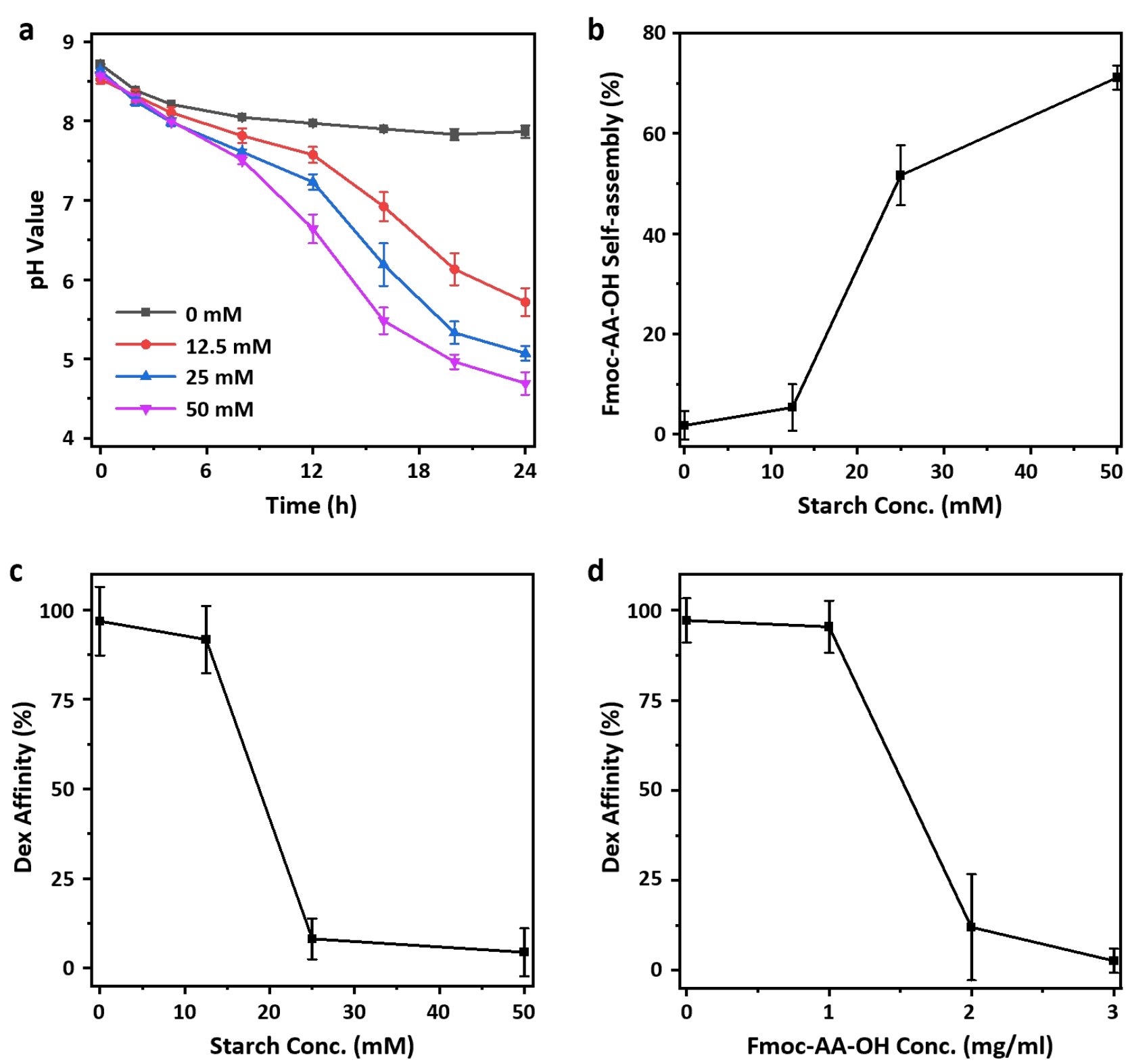

Supplementary Figure 24. (**a**) Time-dependent plots showing pH changes in a dextran-in-PEG ATPS containing SH/GOx-loaded Psome-Dex protocells, Fmoc-AA-OH (*ca.* 2 mg/ml), and different starch concentrations (0-50 mM). (**b**) Corresponding plots showing the percentage level of Fmoc-AA-OH peptide self-assembly in the ATPS at 24 h after addition of different starch concentrations. (**c**) Plot showing the percentage of peptide fibre-modified Psome-

Dex protocells located within dextran-rich droplets (dextran affinity) in ATPS samples containing different starch concentrations (0-50 mM). Fmoc-AA-OH concentration: ca. 2 mg/ml. (**d**) Plot showing the number of peptide-fibre-modified Psome-Dex protocells located within dextran-rich droplets (dextran affinity) in ATPS samples containing different Fmoc-AA-OH concentrations (0-3 mg/ml) at a starch concentration: ca. 50 mM. For (**c**) and (**d**), reaction mixtures were incubated under vortex agitation for ca. 24 h, and the number of transferred proteinosomes was quantified from CLSM images. Psome-Dex density: 0.6×10^7 /ml. Error bars: n = 3 (repeated groups of samples), mean values ± SD.

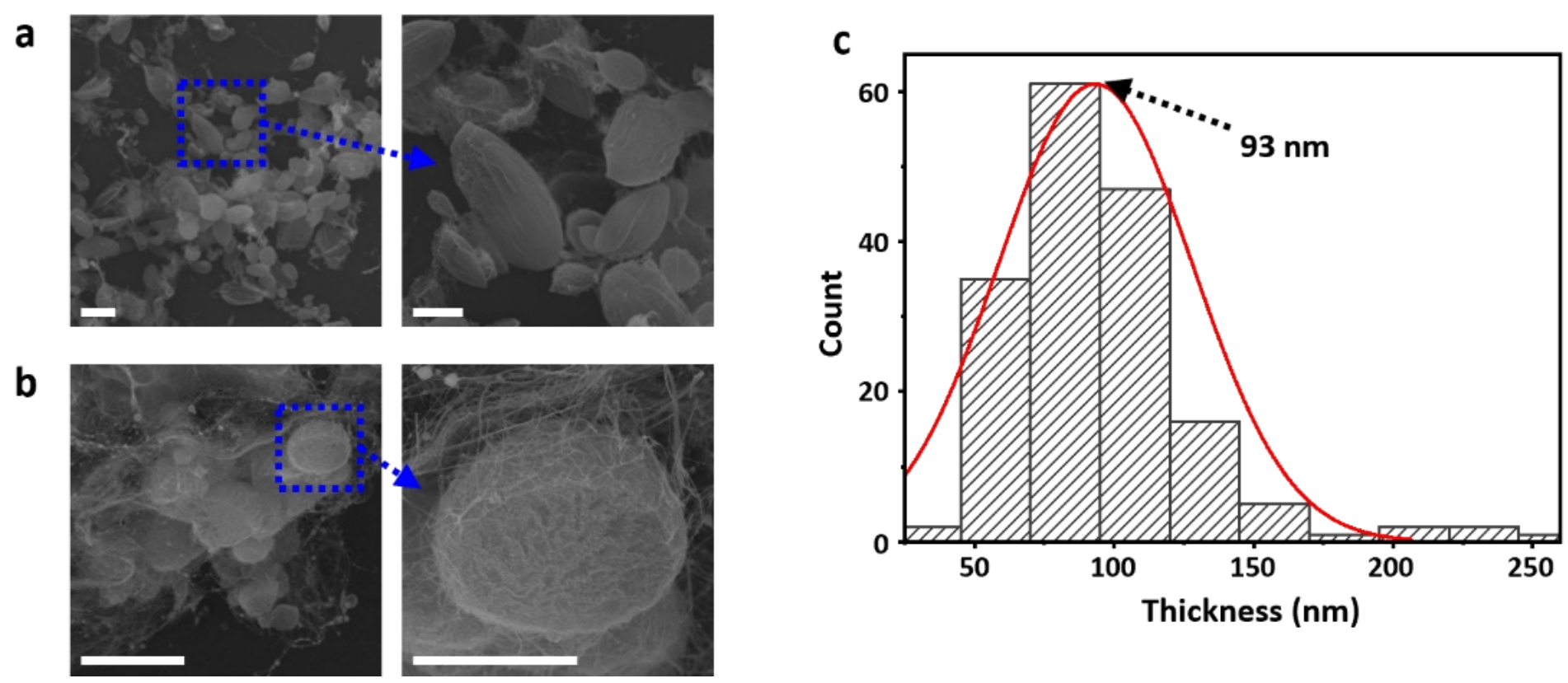

Supplementary Figure 25. SEM images of a population of SH/GOx/Psome-Dex protocells in the absence (**a**) and presence (**b**) of membrane-attached self-assembled Fmoc-peptide fibrils on the surface. (**c**) Distribution of fiber thicknesses measured on the surface of membrane-modified Psome-Dex protocells. n = 172, mean value = 93.3 nm.

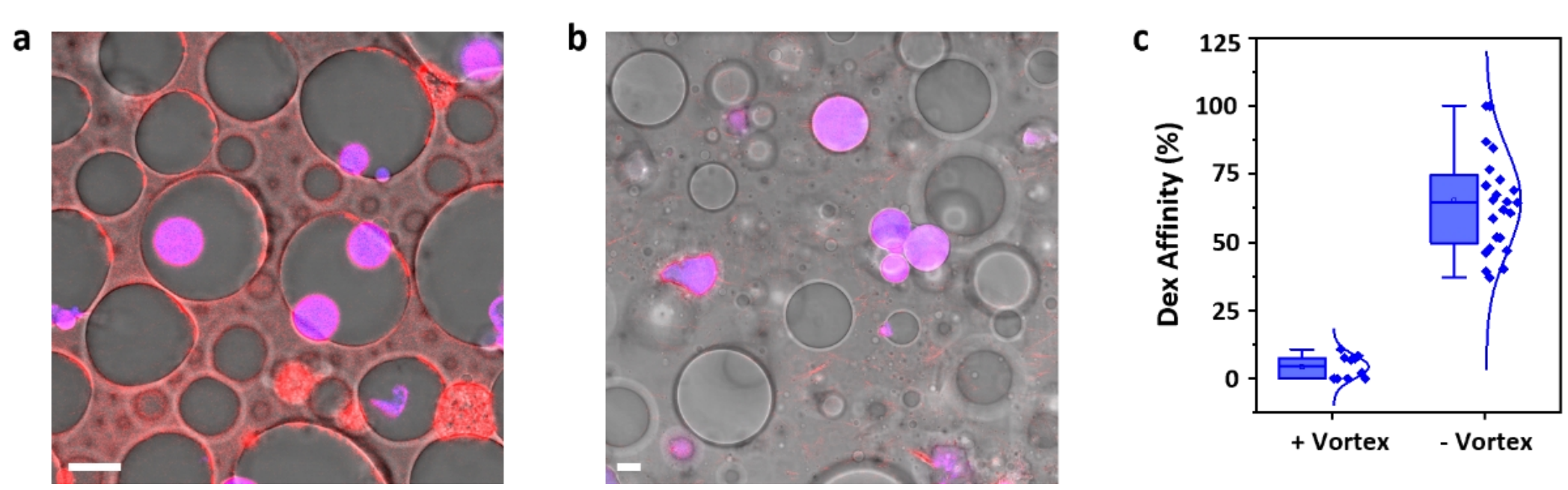

Supplementary Figure 26. (**a,b**), CLSM images of an ATPS containing a population of peptide fibril-coated Psome-Dex protocells recorded 24 h after addition of glucose (10 mM); no vortex (**a**) and after vortexing (**b**) showing that mechanical agitation is required to transfer the modified protocells from the dextran droplets to the continuous PEG phase. (**c**) Corresponding statistical analysis of dextran affinity of the Psome-Dex constructs shown in (**a**) and (**b**). Box plots show the median (centre line), the interquartile range (box, 25%–75%), and the minimum and maximum values (whiskers). Error bars: n = 10, SD = 4.2 % (+ vortex); n = 24, SD = 18.9 % (- vortex). Vortexing was often required to transfer the membrane-modified protocells due to a general increase in viscosity throughout the

ATPS associated with adventitious formation of the dipeptide network in the continuous phase. Fmoc-AA-OH concentration, *ca.* 2.5 mg/ml. Number density of Psome-Dex, *ca.* 0.6 x 10^7/ml. Scale bar, 20 µm.

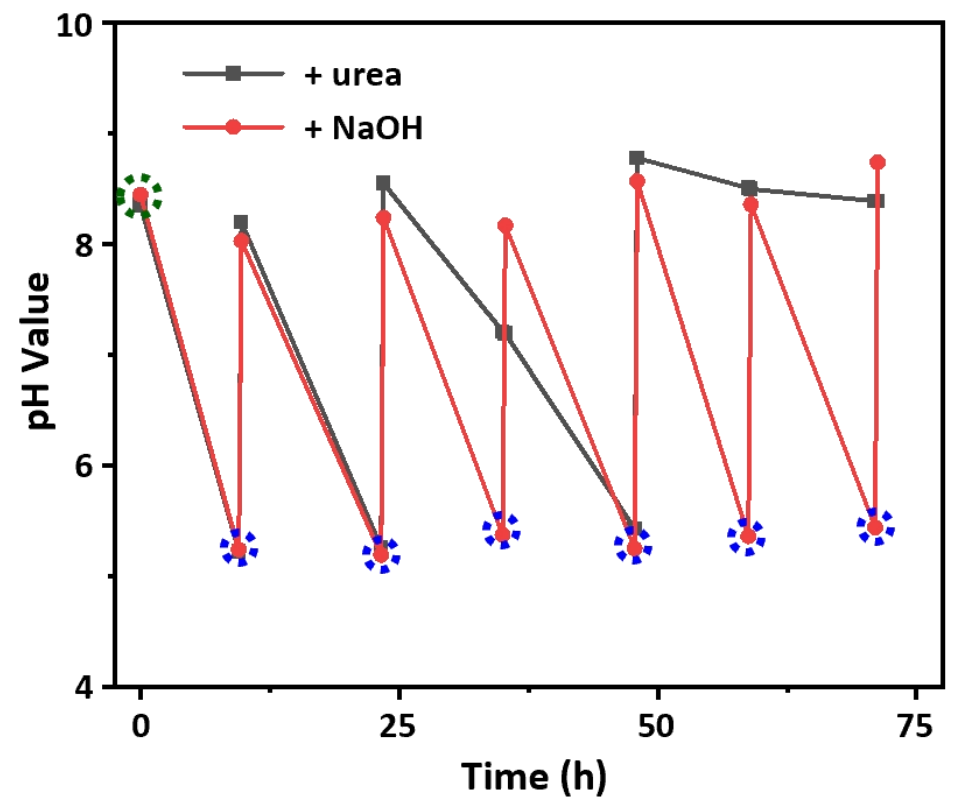

Supplementary Figure 27. Time-dependent variation of pH within an ATPS containing SH/GOx-loaded Psome-Dex protocells, free urease and starch. Urease concentration, ca. 2 unit /ml; starch concentration, 75 mM; Psome-Dex protocell density, 0.6 × 10^7 /ml. Following starch addition (green dashed circle), the bulk pH decreased and was restored by sequential addition of urea (ca. 5 mM, green) or NaOH (ca. 2.5 mM, red) whenever the pH dropped below ca. 5.3 (blue dashed circles).

.

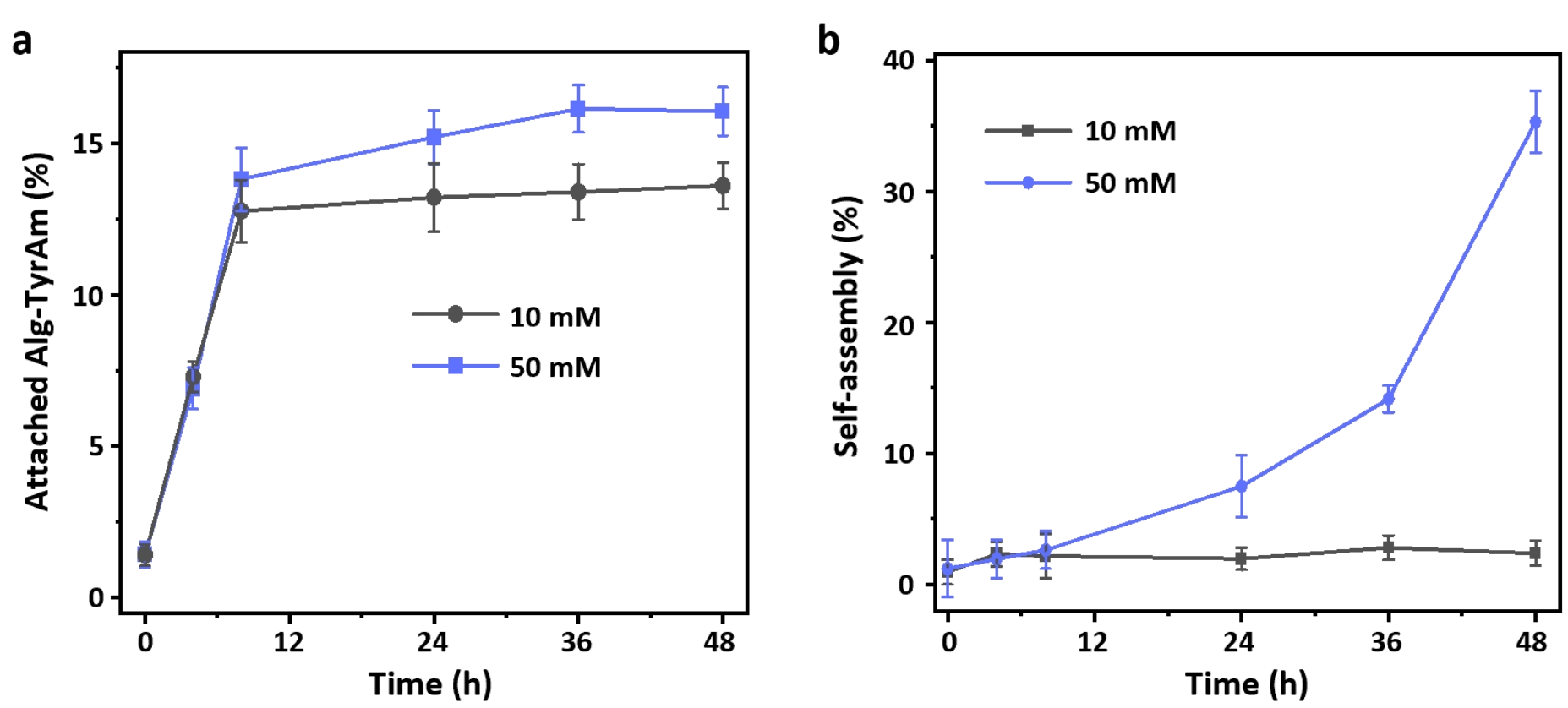

Supplementary Fig. 28. (**a**) Time-dependent plots of the percentage of Alg-TyrAm associated with the membrane of HRP/Psome-PEG responder protocells on receipt of a H_2O_2 diffusive signal from SH/GOx/Psome-Dex recruiter protocells after addition of 10 or 50 mM starch solution. The responder protocells are subsequently recruited into the dextran droplets of the ATPS. (**b**) Time-dependent plots of the percentage of Fmoc-AA-OH peptide fibres associated with SH/GOx/Psome-Dex recruiter protocells after the single addition of 10 or 50 mM starch to an ATPS. The recruiter protocells are initially located within the dextran droplets and subsequently co-located with recruited Alg-TyrAm-modified Psome-PEG responder protocells. Peptide fibre-modification occurs only at a starch concentration of 50 mM. Under these conditions the modified recruiter protocells are dispatched into the PEG continuous phase of the ATPS. Error bars: n = 3 (repeated groups of samples), mean values ± SD.

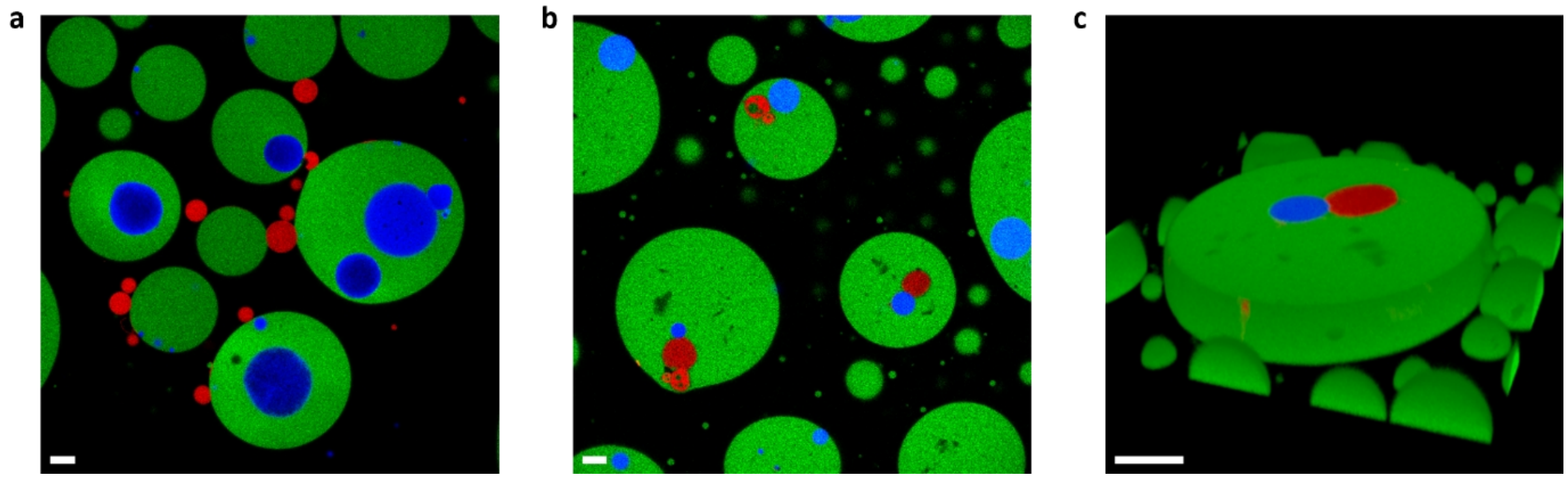

Supplementary Figure 29. (**a**) and (**b**) CLSM images of a dextran-in-PEG ATPS containing segregated populations of Psome-Dex (starch hydrolase/GOx-loaded, DyLight 405-labelled, blue) and Psome-PEG (HRP-loaded, RITC-labelled, red), along with dissolved tyramine-derivatized alginate (ca. 1 mg/mL) and starch (ca. 10 mM). Images were acquired at 0 (**a**) and 24 h (**b**) after starch addition. (**c**) 3D-stacked CLSM image of a dextran-rich droplet in the ATPS, showing co-localized Psome-Dex and Psome-PEG protocells. Dextran is FITC-labelled. Proteinosome number densities: Psome-Dex, 0.6 x 10^7/mL; Psome-PEG, 1.2 x 10^7/mL. Scale bars, 20 µm.

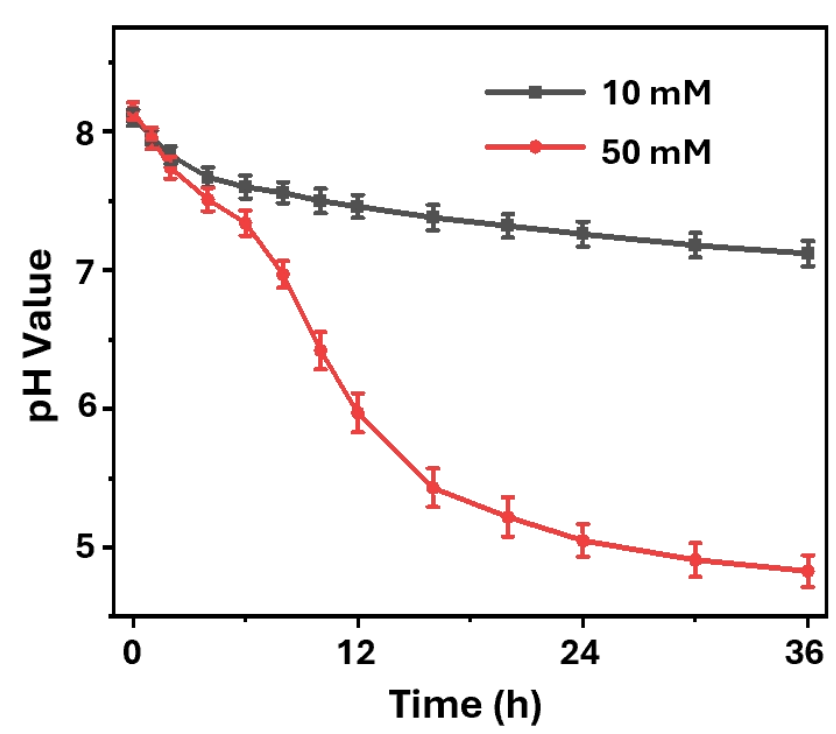

Supplementary Fig. 30. Plots of pH against time in an ATPS containing SH/GOx/Psome-Dex recruiter protocells and HRP/Psome-PEG responder protocells after addition of a single input of starch at concentrations of 10 or 50 mM. Error bars: n = 3 (repeated groups of samples), mean values ± SD.

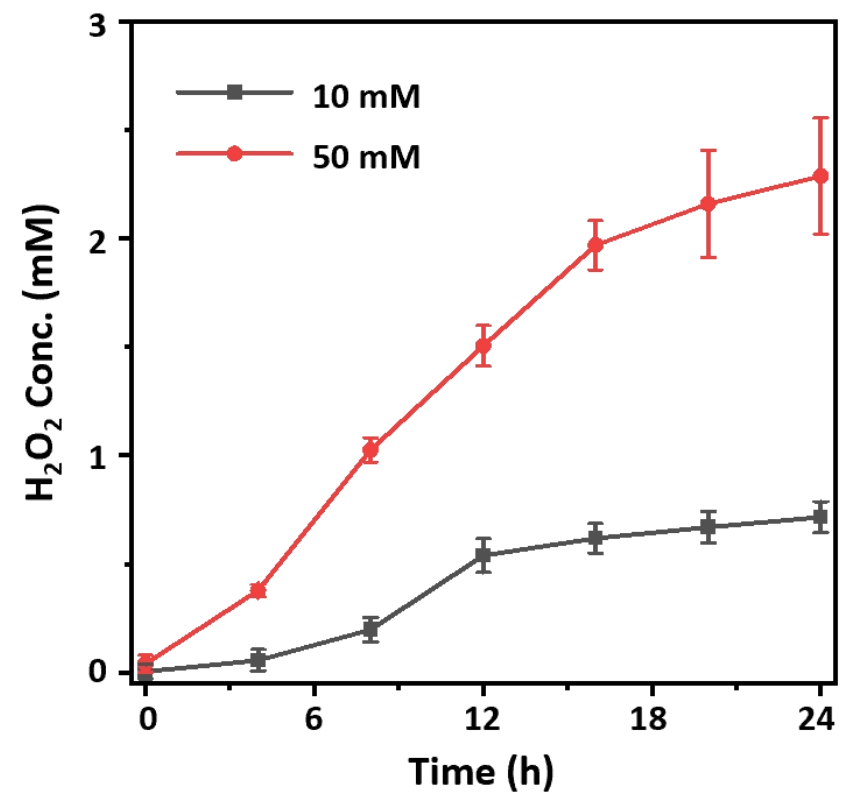

Supplementary Figure 31. Time-dependent plots showing the generation of H_2O_2 within an ATPS containing SH /GOx-loaded Psome-Dex and HRP-loaded Psome-PEG protocells after starch addition (10 and 50 mM). Protocell density: Psome-Dex, 0.6 x 10^7/ml; Psome-PEG, 1.2 x 10^7/ml. The H_2O_2 concentrations were determined by collection of sample aliquots at designated time points, followed by reaction with ABTS (20 mM) in the presence of HRP (ca. 20 unit/ml) and measurement of the UV absorbance at 420 nm. Alg-TyrAm was absent in all experiments to prevent consumption of H_2O_2 during the concentration measurements. Given that complete transfer of the Psome-PEG constructs occurs within 9 h in the presence of Alg-TyrAm at 10 mM starch, a signal threshold level of < 0.35 mM was estimated from the above data (black plot). Error bars: n = 3 (repeated groups of samples), mean values ± SD.

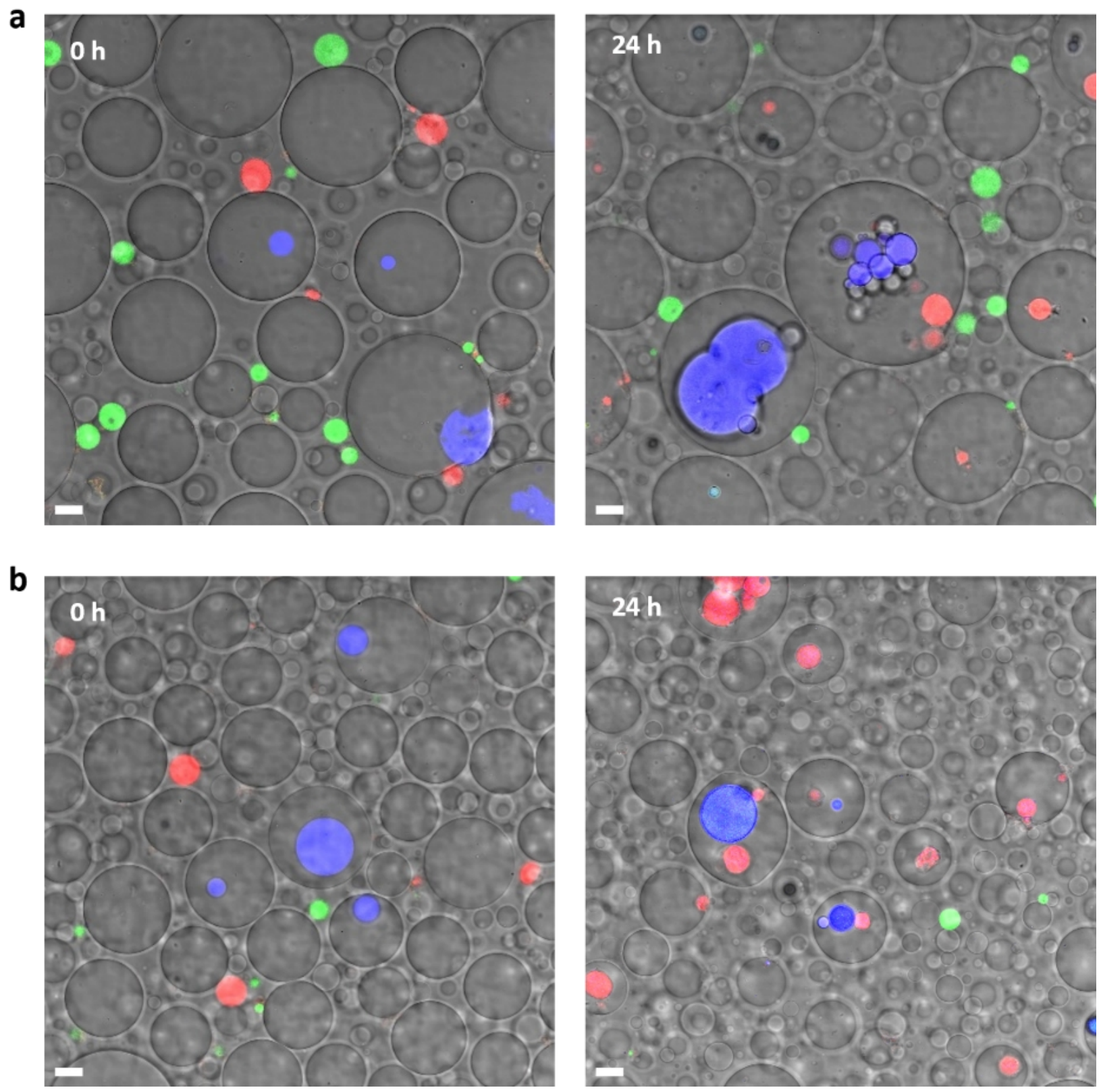

Supplementary Figure 32. (**a**) CLSM images showing indirect recruitment in ternary populations of protocells within a dextran-in-PEG ATPS. Recruiter protocells: starch hydrolase (α-amylase/amyloglucosidase)-loaded Psome-Dex (DyLight 405-labelled, blue; 0.45 × 10^7 /ml). Messenger protocells: GOx-loaded Psome-PEG (FITC-labelled, green; 0.6 × 10^7 /ml). Responder protocells: HRP-loaded Psome-PEG (RITC-labelled, red; 0.6 × 10^7 /ml). Upon starch addition (10 mM), responder protocells were recruited into dextran-rich droplets as a result of H_2O_2 generation by the messenger protocells. (**b**) CLSM images showing selective recruitment in ternary populations of protocells within a dextran-in-PEG ATPS. Recruiter protocells: starch hydrolase (α-amylase/amyloglucosidase)-loaded Psome-Dex (DyLight 405-labelled, blue; 0.45 × 10^7 /ml). Non-responder protocells: Psome-PEG (FITC-labelled, green; 0.6 × 10^7 /ml). Responder protocells: GOx/HRP-loaded Psome-PEG (RITC-labelled, red; 0.6 × 10^7 /ml). Upon starch addition (10 mM), responder protocells were recruited into dextran-rich droplets, whereas non-responders remained at their original positions. Images were acquired at 0 and 24 h after starch addition. Scale bars, 20 µm.

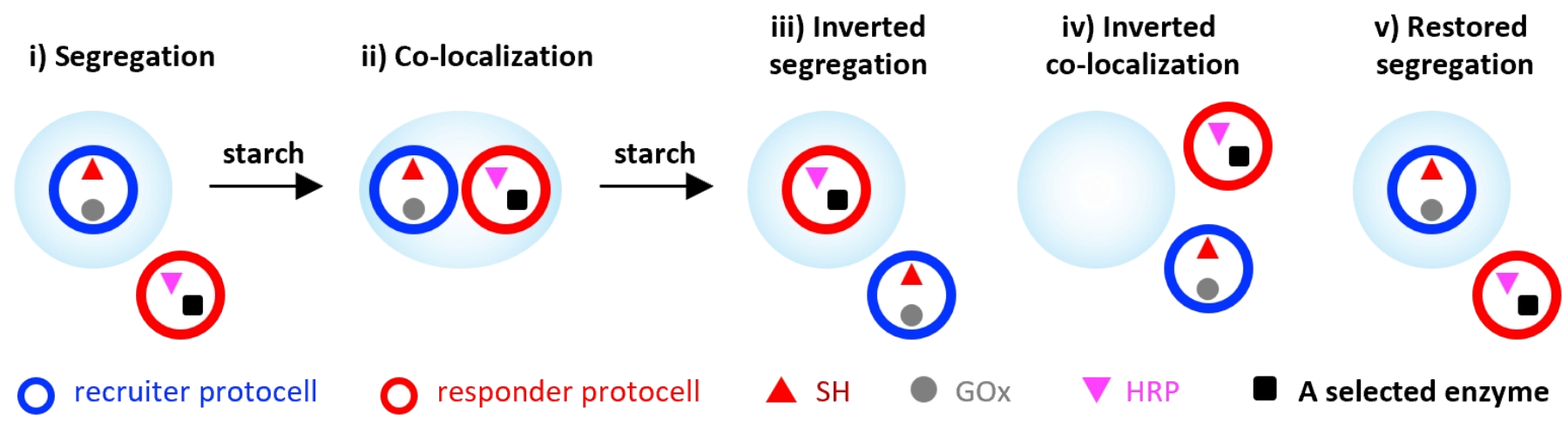

Supplementary Figure 33. Signal-induced recruitment and targeted dispatchment in phase-separated protocell networks. a, Schematic of signal-induced protocell recruitment/co-localization ((i) and (ii)) and subsequent modes of signal-mediated targeted protocell dispatchment ((iii-v)) in spatially organized binary populations of enzyme-containing proteinosomes. SH- and GOx-containing recruiter protocells (blue, Psome-Dex), initially located within the dextran droplets of an ATPS (light blue filled circles), emit glucose, gluconic acid (H^+) and H_2O_2 diffusive signals in response to a starch input. Responder protocells (red, Psome-PEG) loaded with HRP (mode (iii)), HRP/GOx (mode (iv)), or HRP/AP (mode (v))) are located initially in an Alg-TyrAm-containing PEG continuous phase and undergo HRP-mediated surface modification on delivery of a H_2O_2 signal. Consequently, Alg-TyrAm-coated Psome-PEG constructs transfer into the dextran droplets due to changes in wettability. Subsequent dispatchment of the recruiter protocells from the dextran droplets to invert the initial spatial organization (mode (iii)) is achieved in the presence of Fmoc-AA-OH by the pH (gluconic acid)-induced coating of the GOx-containing Psome-Dex sub-population with Fmoc-dipeptide fibers. An inter-protocellular signal-induced change in wettability is used to expel both the recruiter and responder protocells from the dextran droplets by supplementing the captured responder protocells with GOx (mode (iv)). Specific dispatchment of the recruited protocells to restore the initial segregation (mode (v)) is achieved by supplementing the HRP/Psome-PEG constructs with AP and exploiting the proton efflux from the recruiter protocells to initiate Fmoc-TyrP dephosphorylation and PEG-compatible peptide fibre assembly.

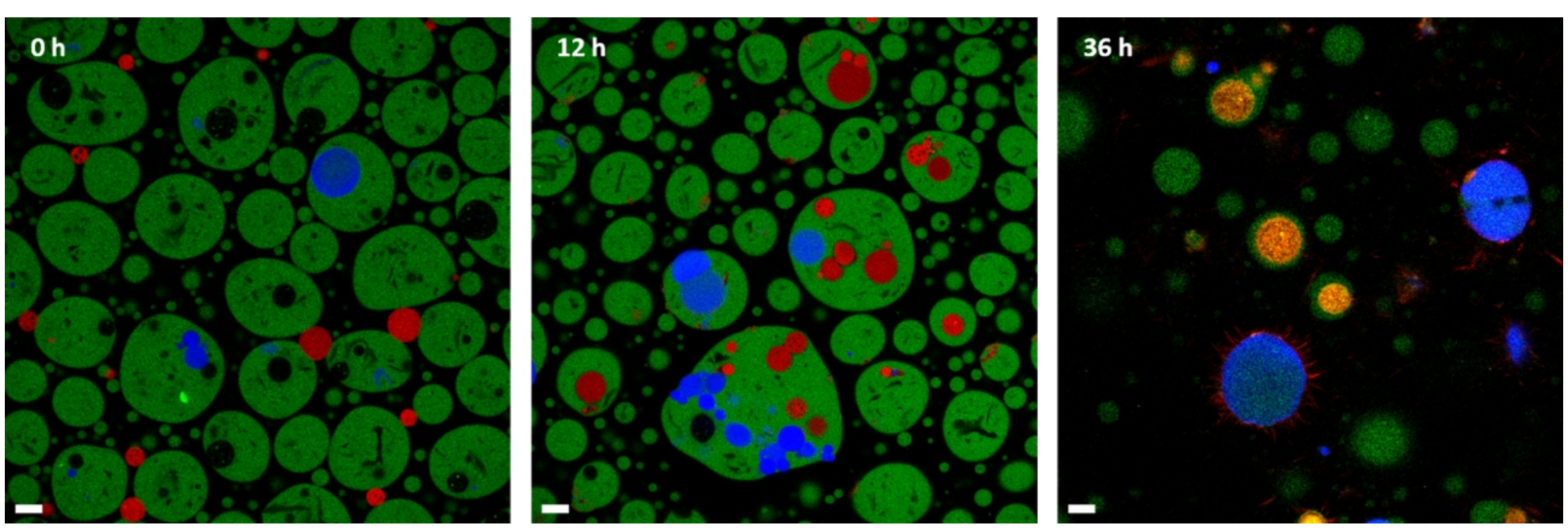

Supplementary Figure 34. Time-sequence of CLSM images showing inverted segregation of a binary population of protocells within a dextran-in-PEG ATPS. Recruiter protocells: starch hydrolase/GOx-loaded Psome-Dex (DyLight 405-labelled, blue, 0.6 × 10^7 /ml); Responder protocells: HRP-loaded Psome-PEG (RITC-labelled, red, 1.2 × 10^7 /ml). Tyramine-derivatized alginate (ca. 1 mg/ml), Fmoc-AA-OH (ca. 2 mg ml^{-1}), and starch (ca. 50 mM) are present in the system. Dextran is FITC-labelled. Scale bars, 20 µm.

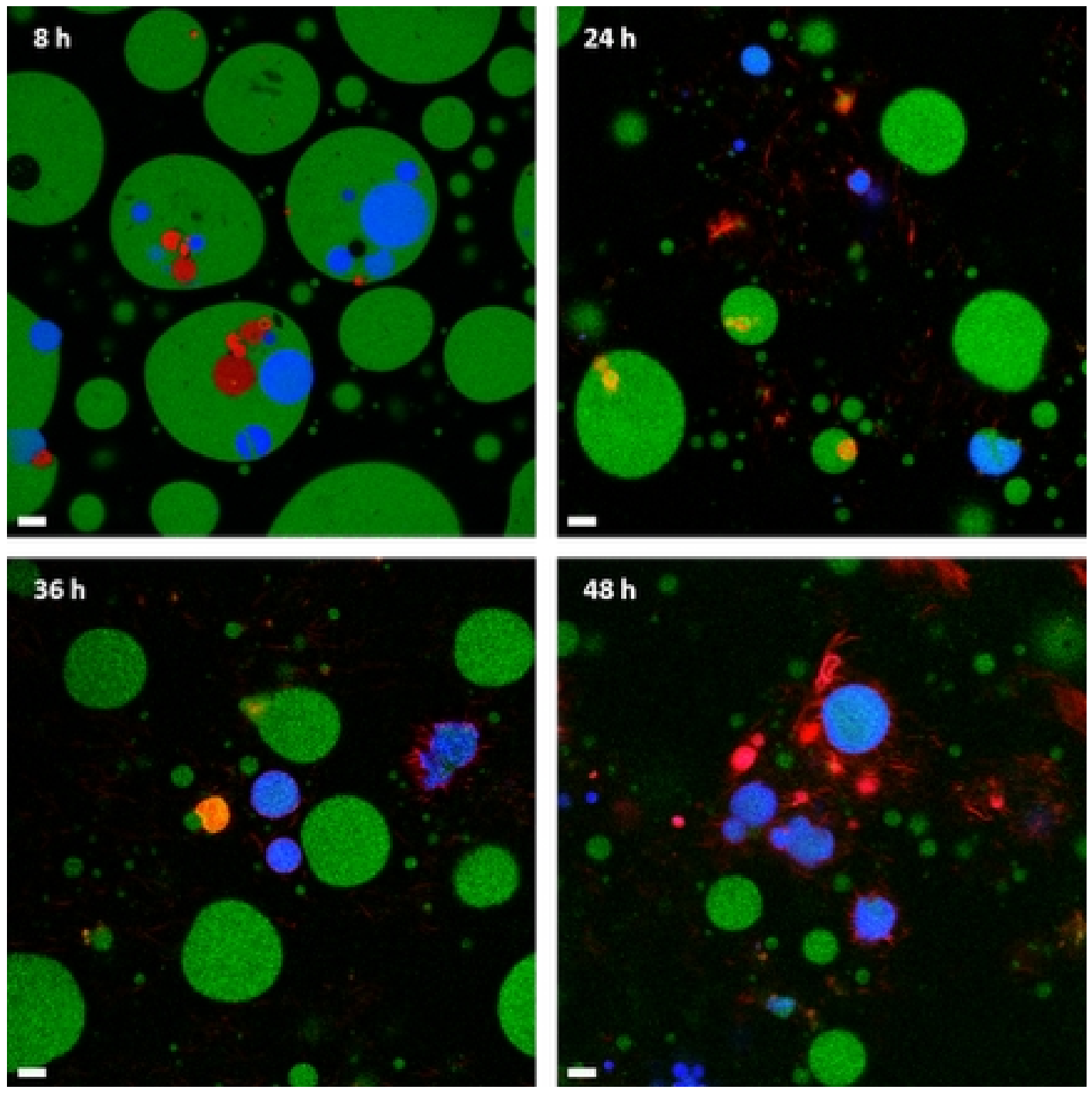

Supplementary Figure 35. Time-sequence of CLSM images showing the co-expulsion of recruiter and responder protocells into the continuous PEG-rich phase within a dextran-in-PEG ATPS. Recruiter protocells: starch hydrolase/GOx-loaded Psome-Dex (DyLight 405-labelled,blue, 0.6 × 10^7 /ml); responder protocells: GOx/HRP-loaded Psome-PEG (RITC-labelled, red, 1.2 × 10^7 /ml). Tyramine-derivatized alginate (ca. 1 mg ml^{-1}), Fmoc-AA-OH (ca. 2 mg ml^{-1}), and starch (ca. 50 mM) were present in the system. Dextran is FITC-labelled. Scale bars, 20 µm.

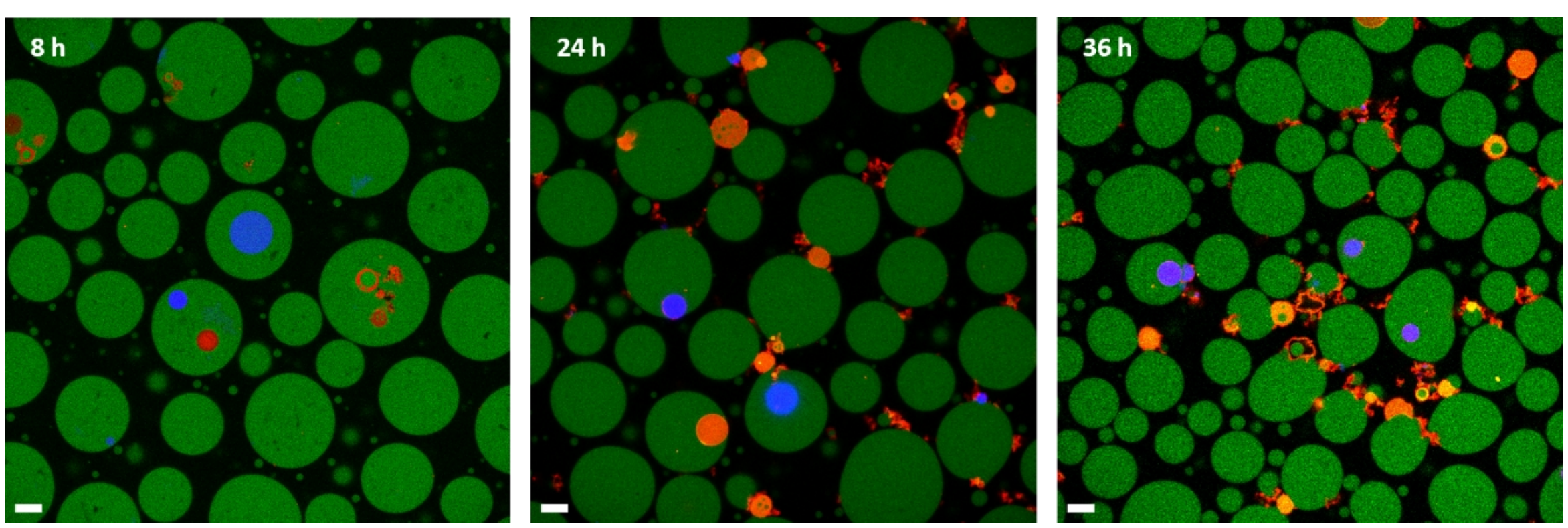

Supplementary Figure 36. Time-sequenced CLSM images showing the expulsion of responder protocells from a binary population of protocells co-localized within dextran-rich droplets in a dextran-in-PEG ATPS. Recruiter protocells: starch hydrolase/GOx-loaded Psome-Dex (DyLight 405-labelled, 0.6 × 10^7 /ml); Responder protocells: HRP/acid phosphatase-loaded Psome-PEG (RITC-labelled, 1.2 × 10^7 /ml). Tyramine-derivatized alginate (ca. 1 mg ml^{-1}), Fmoc-TyrP (ca. 2 mg ml^{-1}), and starch (ca. 50 mM) were present in the system. Dextran was FITC-labelled. Scale bars, 20 µm.

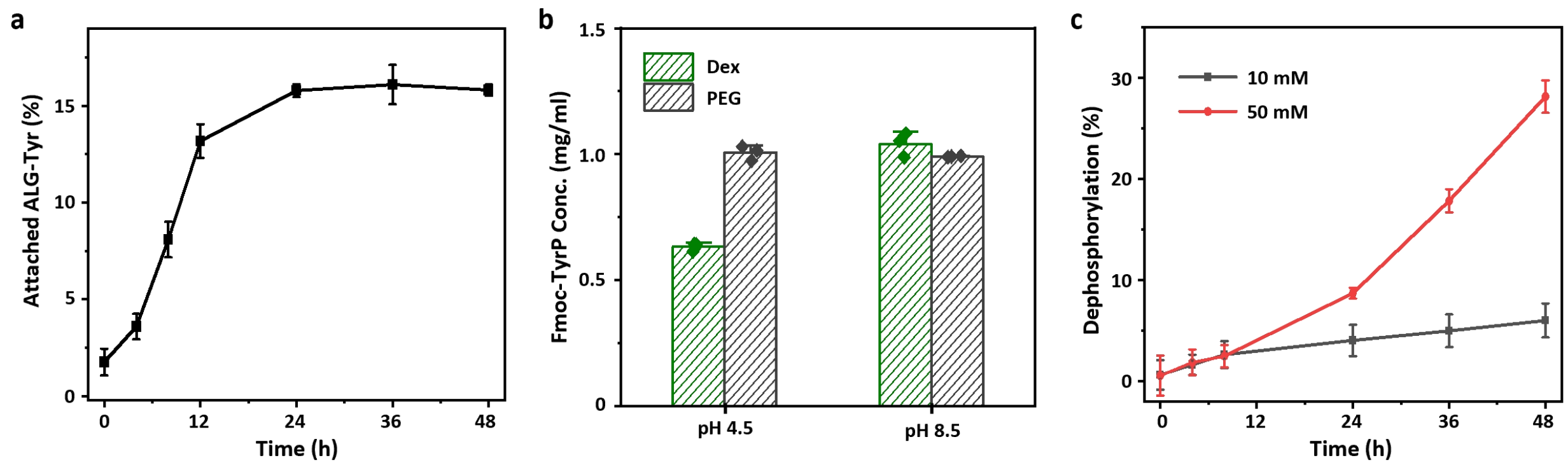

Supplementary Fig. 37. (a) Time-dependent plot of the percentage of Alg-TyrAm associated with the membrane of HRP/AP/Psome-PEG responder protocells on receipt of a H_2O_2 diffusive signal from SH/GOx/Psome-Dex recruiter protocells after addition of a single input of 50 mM starch solution. The ATPS also contains Fmoc-TyrP. The responder protocells are subsequently recruited into the dextran droplets of the ATPS. (**b**) Bar chart showing distribution of dissolved Fmoc-TyrP at pH 4.5 and 8.5 in a Dex-in-PEG ATPS. (**c**) Plots of percentage Fmoc-TyrP dephosphorylation against time in ATPS containing different concentrations of starch (10 and 50 mM). At 50 mM, the recruited Alg-TryAm-modified responder protocells are subsequently dispatched back into the continuous PEG phase due an outer coating of PEG-compatible peptide fibres. Error bars: n = 3 (repeated groups of samples), mean values ± SD.

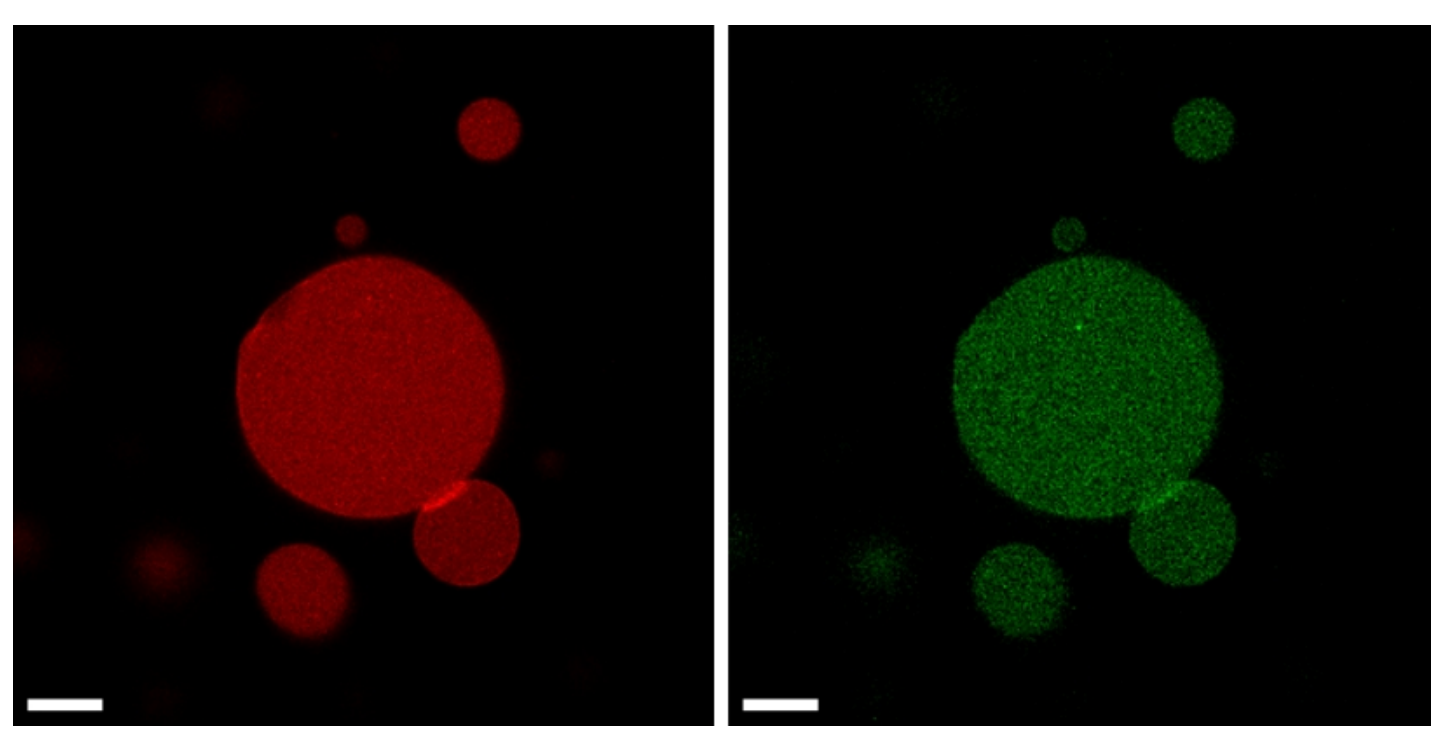

Supplementary Figure 38 CLSM images of RITC-labelled (red filtered left image) Psome-PEG protocells containing FITC-labelled (green filtered image, right) DNase I (right). Scale bars, 20 µm.

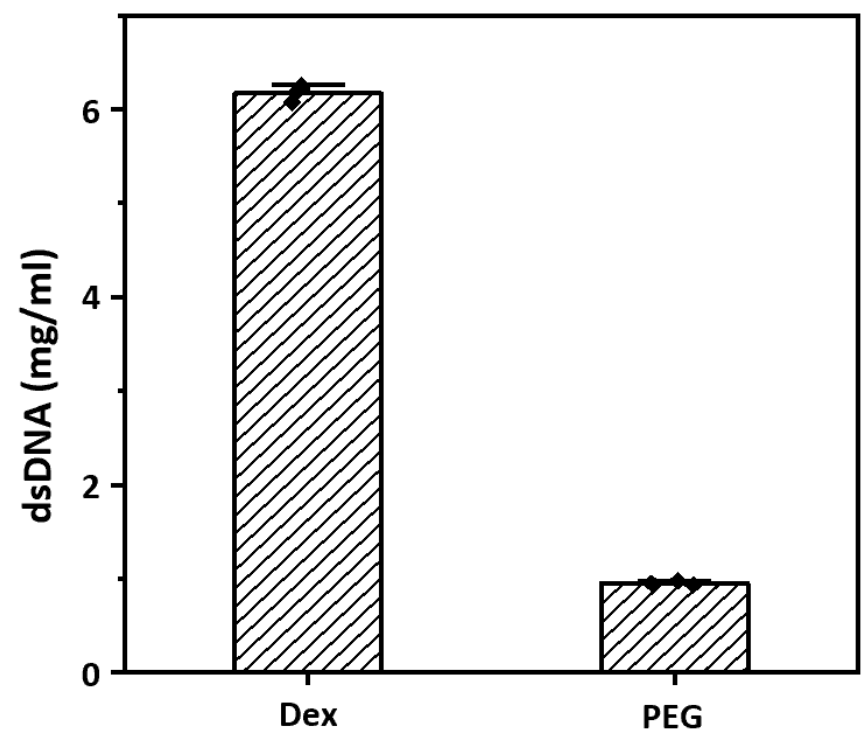

Supplementary Fig. 39. Bar chart showing distribution of dsDNA in a Dex-in-PEG ATPS in the presence $MgCl_2$ (6 mM). Corresponding Dex : PEG partition constant is approximately 6.5. Bulk concentration of Fmoc-AA-OH monomers is ca. 2 mg/ml. Error bars: n = 3 (repeated groups of samples), mean values ± SD.

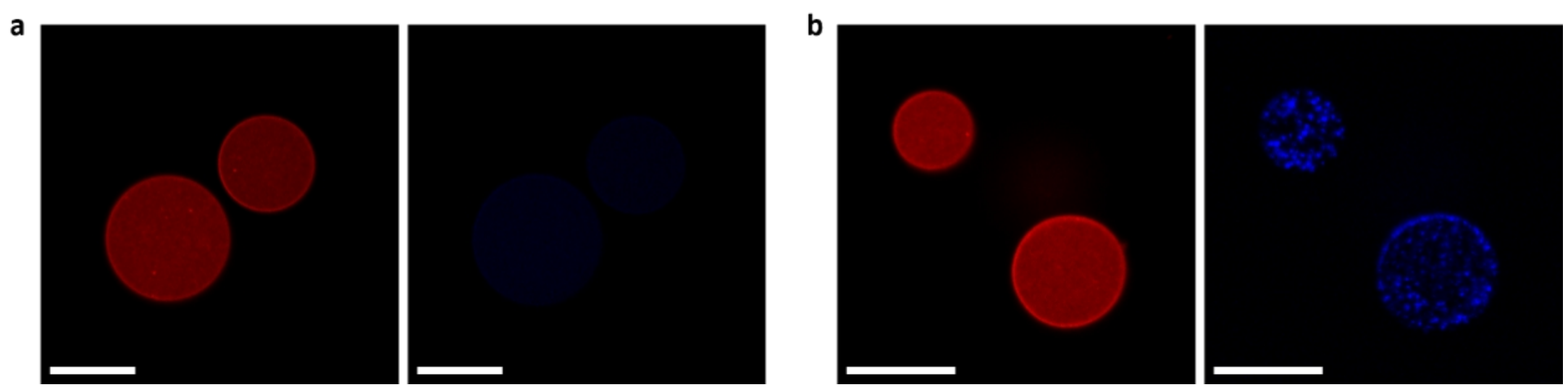

Supplementary Figure 40. CLSM images of RITC-labelled PDDA-loaded Psome-PEG protocells dispersed in aqueous solutions containing DAPI (dsDNA dye, blue fluorescence, 0.02 mg/ml) in the absence (**a**) or presence of dsDNA (M_W, 66-132 kDa; 0 2 mg/ml (**b**). DAPI-stained dsDNA/PDDA-based condensates formed within the Psome-PEG protocells by electrostatic complexation after the addition of dsDNA in the exterior aqueous media, confirming dsDNA uptake through the crosslinked proteinosome membrane. PDDA Mw: 100-200 kD. Scale bars, 20 µm.

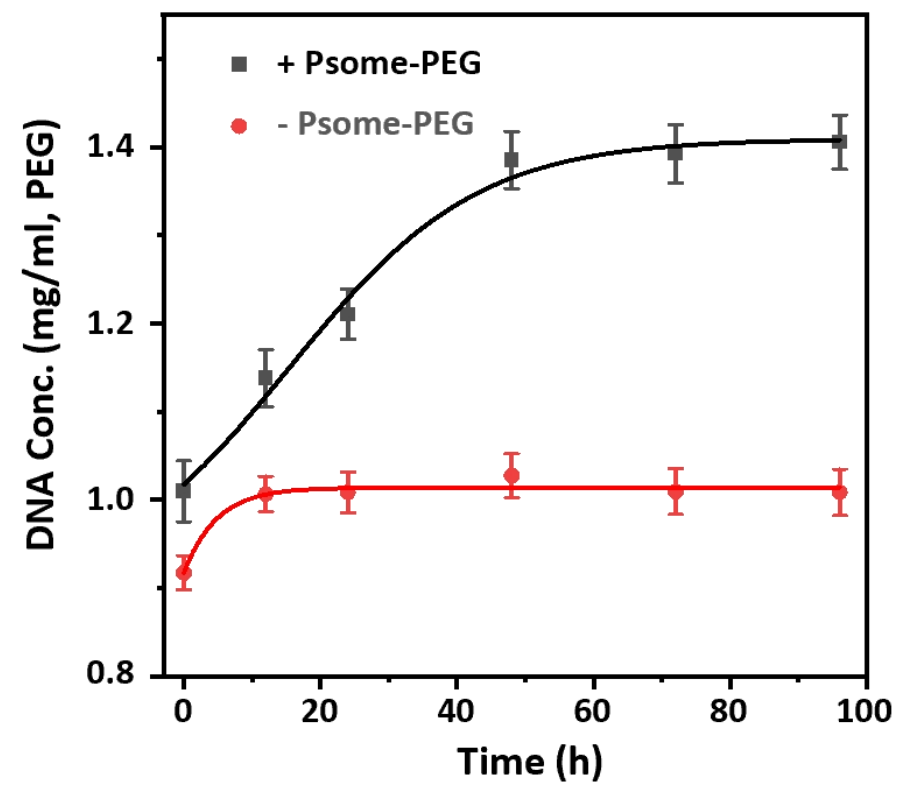

Supplementary Figure 41. Time-dependent plots showing degradation of dsDNA induced by Alg-TyrAm-modified GOx/HRP/DNase I-loaded Psome-PEG protocells dispersed within an ATPS. The degradation process is monitored by measuring the concentration of dsDNA cleavage products in the continuous PEG-rich phase. dsDNA concentration: ca. 2 mg/ml. Reaction pH value: ca. 6.5. Psome-PEG density: 0 or 0.6 × 10^7/ml. Error bars: n = 3 (repeated groups of samples), mean values ± SD.

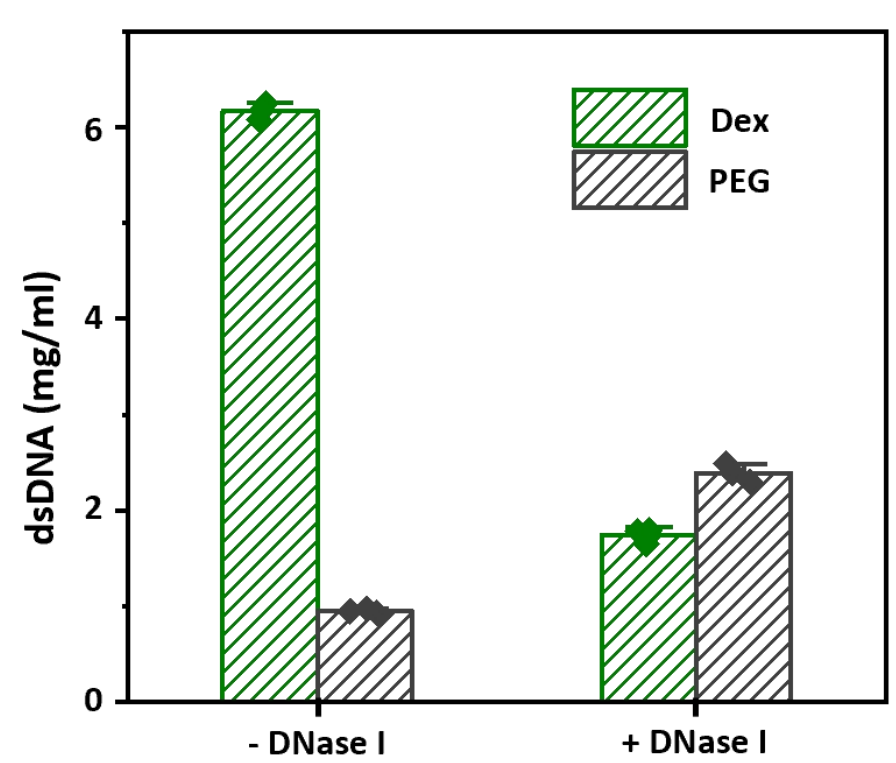

Supplementary Fig. 42. Bar charts showing the distribution of dsDNA in a Dex/PEG ATPS containing $MgCl_2$ (6 mM) and in the presence (100 IU/mL) or absence of DNase I after incubation at room temperature for 24 h with Dex : PEG partition constants of ca. 6.5 (- DNase I) and 1.4 (+ DNase I). The decreased concentration of DNA in the dextran droplets after DNase-induced degradation is due to the increased solubility of the cleavage products in the PEG phase. Monitoring the changes in DNA concentration in the PEG phase over time was used as a proxy for DNase activity in the dextran droplets. Error bars: n = 3 (repeated groups of samples), mean values ± SD.
